# Longevity of companion dog breeds: those at risk from early death

**DOI:** 10.1038/s41598-023-50458-w

**Published:** 2024-02-01

**Authors:** Kirsten M. McMillan, Jon Bielby, Carys L. Williams, Melissa M. Upjohn, Rachel A. Casey, Robert M. Christley

**Affiliations:** 1 Dogs Trust, London, UK; 2 Liverpool John Moores University, Liverpool, UK

**Keywords:** Phylogenetics, Biological anthropology, Population dynamics

## Abstract

The companion dog is one of the most phenotypically diverse species. Variability between breeds extends not only to morphology and aspects of behaviour, but also to longevity. Despite this fact, little research has been devoted to assessing variation in life expectancy between breeds or evaluating the potential for phylogenetic characterisation of longevity. Using a dataset of 584,734 unique dogs located within the UK, including 284,734 deceased, we present variation in longevity estimates within the following: parental lineage (purebred = 1 breed, crossbred ≥ 2 breeds), breed (n = 155), body size (large, medium, small), sex (male, female) and cephalic index (brachycephalic, mesocephalic, dolichocephalic). Survival estimates were then partitioned amongst phylogenetic clades: providing evidence that canine evolutionary history (via domestication and associated artificial selection) is associated with breed lifespan. This information provides evidence to inform discussions regarding pedigree health, whilst helping current/prospective owners, breeders, policy makers, funding bodies and welfare organisations improve decision making regarding canine welfare.

## Introduction

The modern domesticated dog (*Canis lupus familiaris*) includes over 400 genetically distinct breeds, representing considerable variation in morphological, behavioural, and physiological phenotypes^1,2^. However, variability between breeds also extends to life expectancy^3–5^. In spite of this, little research has been devoted to assessing variation in longevity between canine sub-populations or evaluating the potential for phylogenetic characterisation of longevity. Providing these estimates would help veterinarians and researchers highlight health and welfare challenges within the field, whilst also managing stakeholder expectations (including current and prospective owners), with regards to future responsibilities and duration of dog-owner relationship.

Though it is widely accepted that dogs were domesticated from an ancient wolf ancestor^6,7^, findings diverge on the specific timing, location and number of domestication sites^8–18^. However, a date of around 16,000 cal BP (calibrated years before the present) is generally accepted as the timing of domestication, based on robust archaeological and genomic evidence^6,15,17–19^. Despite this ~ 16,000 year timeline, the spectacular phenotypic diversity observed amongst breeds is thought to have originated much more recently, largely through intense artificial selection. This has led to the development of closed breeding populations with limited genetic heterogeneity^20^. The loss of genetic diversity can be attributed to a significant population bottleneck event: the generation of pedigree breeds^21–23^. To ensure the persistence of pedigree diversity, strict breeding practices have been emplaced to restrict gene flow between breeds^24^. These practices include: the repeated use of popular sires, breeding to perpetuate desired physical or behavioural characteristics, promotion of the breed barrier rule (i.e., a dog can only become a registered member of a breed, if both parents are registered members), and population maintenance via inbreeding within closed familial lines^25–28^. Additionally, selection pressure towards phenotypic exaggeration to achieve breed standards and/or obtain a competitive advantage when judged at show standards, has been associated with hereditary pathology and conformation disorders^29,30^. Breeding practices focussed on physical appearance solely, often result in reduced attention to canine health, welfare, functionality, and behaviour^31,32^.

Longevity estimates for an average domestic dog varies between 10.0 and 13.7 years of age, with variation depending on populations analysed e.g., country and/or breed specific^3–5,33–38^. However, significant variation in longevity has been reported both within and between purebreds. For example, median estimates for West Highland White Terriers have been reported at 12.7 and 13.5 years of age, while estimates for Rottweilers have been substantially lower at 8.0 and 8.4 years of age^3,4^. Furthermore, variation in longevity has been reported between pure (parental lineage; PL = 1 breed) and crossbred (PL ≥ 2 breeds) dogs, with most research reporting longer life expectancies within crossbreeds^34,36,39,40^ (however see ^41^). Within a subset of UK and Japanese canine populations, a crossbred survival advantage of 1.2 and 1.3 years was reported in comparison with their purebred counterparts, respectively^3,36^. Consequently, it has been hypothesised that the longevity advantage presented in crossbred dogs may be due to the reduction in homozygous deleterious genes, along with non-genetic differences between and within pure and crossbred populations e.g., management styles^31^. The former would suggest the presence of ‘hybrid vigor’ within canine populations^42^. Hybrid vigor, or heterosis, is the increase in stature, biomass, and fertility that characterizes the progeny of crosses between diverse parents, such that the progeny is superior to the better of the two parents^43^. Inbreeding depression describes the converse effect, i.e., the decline in quantitative measures of fitness upon homozygosis of alleles (i.e., inbreeding)^43^. At present, there is little evidence to support the existence of either evolutionary process (i.e., hybrid vigor or inbreeding depression) within domestic dogs^3,28,42^. However, nearly 700 inherited disorders and traits have been described in the domestic dog^44^, including hip dysplasia, brachycephalic obstructive airway syndrome, cardiomyopathies, endocrine dysfunctions, and blood disorders^11,29,45,46^. As a result, the burden of disease within most purebred populations has become one of the most important issues in canine welfare. It is imperative that future discussions are open and multidisciplinary, as they have significant implications on the ethics of breeding practices, along with the quality of life and longevity of canine breeds.

In addition to breed status, body size, sex, and cephalic index (the ratio between the width and length of skull), have previously been associated with canine longevity^33,47,48^. Mammalian lifespan varies strongly with body size, such that large species tend to outlive smaller species^49,50^. However, within species, small body size is generally associated with greater longevity^51^. The domesticated dog aligns with this latter trend and body size is generally considered to be the greatest predictor of their lifespan^28,35,47,48,52–59^. Despite this fact, substantial survival differences are frequently reported between breeds of similar size^56,60–62^, with larger dogs reported to have the highest morbidity^3,33,52,63^. Female survival advantage is also well documented in mammalian species. A recent study compiling demographic data from 134 mammal populations (encompassing 101 species), reported an average 18.6% longer median lifespan in females, in comparison with conspecific males^64^. In spite of this, previous studies have suggested that sex bears little influence on canine longevity^38,65^. Popularity of brachycephalic (flat-faced) breeds has been increasing internationally, despite increasing scientific evidence highlighting the significant health and welfare challenges associated with this conformation, in comparison with mesocephalic (medium proportions) or dolichocephalic (long-faced) breeds^66–68^. By identifying and comparing differences in longevity between canine populations e.g., parental lineage (PL), breed, size, sex, and cephalic index, future studies can start to disentangle mechanisms linked to risk of early death. However, very little research attention has been dedicated to: (1) assessing variation in life expectancy between subsets of the canine populations, and (2) the phylogenetic characterisation of canine longevity. This knowledge gap is due, in part, to a lack of informative, comparable, and accessible datasets regarding canine mortality.

To address this shortcoming, we developed a research project involving 18 participants, including rehoming and welfare organisations, breed registries, pet insurance companies, veterinary corporations, and university based veterinary archives. This wealth of data allowed us to partition heterogeneity of survival estimates between populations, to compare estimated longevities, and identify those most at risk from early death. Improving our knowledge of canine survivorship provides an important welfare opportunity for the estimated 12 million companion dogs in the UK^69^. This information will assist current/prospective owners, breeders, policy makers, funding bodies and welfare organisations in decision-making to improve the welfare of companion dogs, while also contributing evidence to the canine pedigree health debate.

## Results

**Figure 1:**
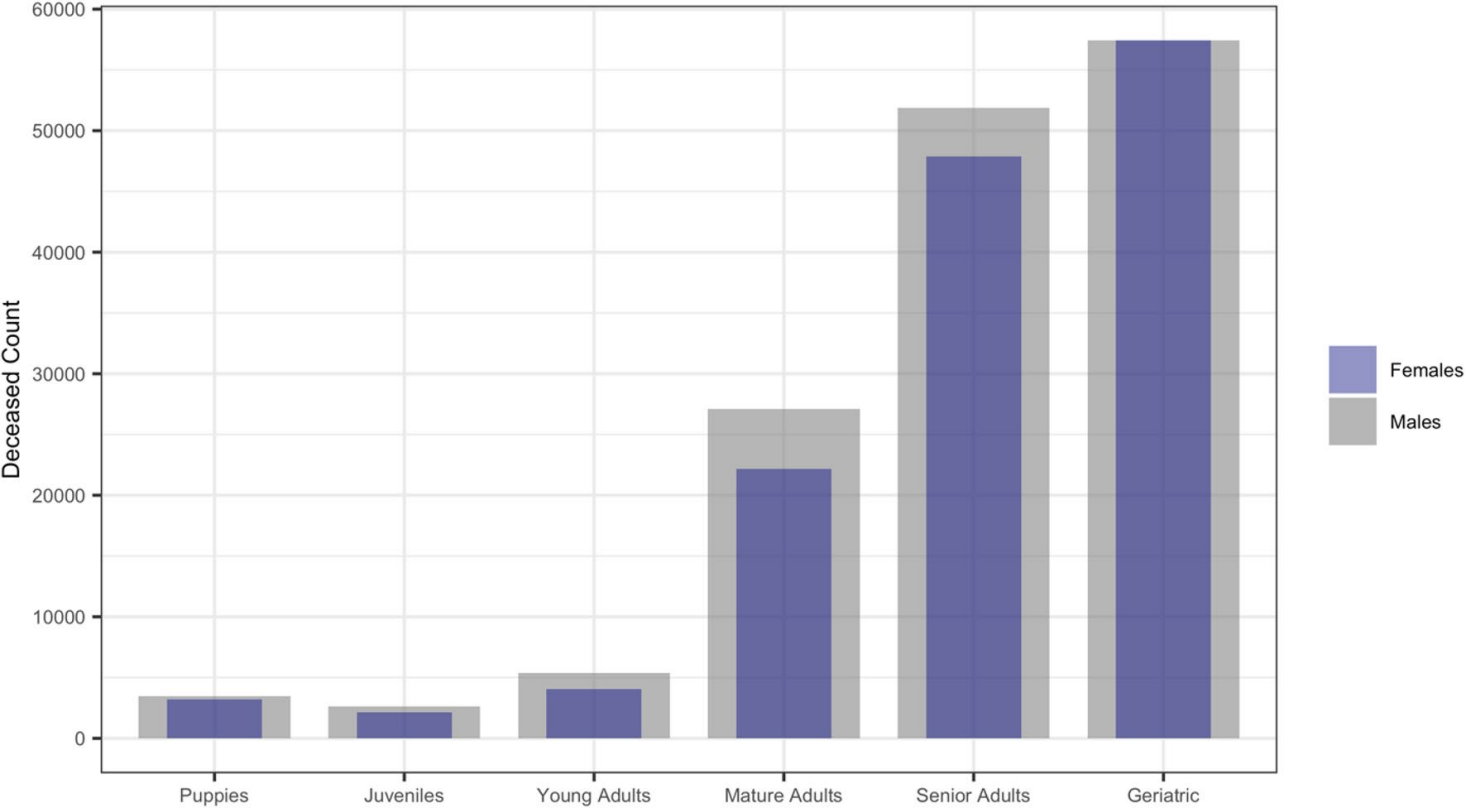
Distribution of deaths from the raw data, within age group, per sex (superimposed). Note higher density of deaths within ‘Puppies’ in comparison with ‘Juveniles’, and male skewed ratios for all age groupings excluding ‘Geriatric’, where ratio is 1:1.

Merged, cleaned and deduplicated data included 584,734 individual dogs located within the United Kingdom (UK), including 284,734 deceased, from 18 sources (see ‘Methods: *Data sources, cleaning and subsetting’* for further details). Individuals within data ranged from 0–24 years of age (x̃ = 12.2, μ = 8.56), consisting of the following demographics: 1.6% Puppies ($n$ = 9298), 2.3% Juveniles ($n$ = 13,226), 6.2% Young Adults ($n$ = 36,269), 29.2% Mature Adults ($n$ = 170,522), 34.0% Senior Adults ($n$ = 198,963) and 26.7% Geriatric ($n$ = 156,456). Distribution of deaths within age group, per sex, are shown in Fig. 1.

There was a 1.1:1 ratio of male (n_♂_ = 300,380) to female dogs (n_♀_= 284,354), and a 4.3:1 ratio of pure (${n}_{E}$ = 473,423) to cross breeds (${n}_{X}$ = 111,311). Within pure breeds, 20.9% were categorised as brachycephalic (n_♂_:n_♀_ 1.1:1), 63.4% were mesocephalic (n_♂_:n_♀_ 1.1:1) and 15.6% were dolichocephalic (n_♂_:n_♀_ 1.1:1). Over 50% of pure breeds were reported as one of the following twelve breeds: Labrador Retriever (9.14%), Staffordshire Bull Terrier (7.73%), English Cocker Spaniel (5.57%), German Shepherd Dog (4.21%), Jack Russel Terrier (4.06%), English Springer Spaniel (3.75%), Yorkshire Terrier (3.05%), Cavalier King Charles Spaniel (2.92%), Border Collie (2.75%), Shih Tzu (2.57%), West Highland White Terrier (2.52%) and French Bulldog (2.44%). Frequency of all purebreds in dataset are listed in Fig. S1.

Small dogs contributed 53.1% of the purebred dataset (n_♂_:n_♀_ 1:1, ${n}_{breeds}$ = 62), whilst medium and large sized dogs made up the remaining 17.6% (n_♂_:n_♀_ 1.1:1, ${n}_{breeds}$ = 44) and 29.3% (n_♂_:n_♀_ 1.1:1, ${n}_{breeds}$ = 49), respectively. Within small breeds, 27.4% were brachycephalic ($n$ = 60,723, ${n}_{breeds}$ = 11), 63.7% were mesocephalic ($n$ = 141,024, ${n}_{breeds}$= 38) and 8.9% were dolichocephalic ($n$ = 19,605, ${n}_{breeds}$ = 13). Within medium breeds, 13.3% were brachycephalic ($n$ = 9709, ${n}_{breeds}$ = 1), 68.7% were mesocephalic ($n$ = 50,030, ${n}_{breeds}$ = 30) and 18.0% were dolichocephalic ($n$ = 13,072, ${n}_{breeds}$ = 13). Within large breeds, 14.4% were brachycephalic ($n$ = 17,671, ${n}_{breeds}$ = 7), 62.6% were mesocephalic ($n$ = 76,699, ${n}_{breeds}$ = 32) and 23.0% were dolichocephalic ($n$ = 28,114, ${n}_{breeds}$ = 10).

### Survival analysis

Percentage of total population expiring within each age grouping, $\theta \left\{{X}_{i}\right\}$, are listed within Table 1: highlighting that survival probability is greatest for Juveniles, similar amongst Puppies and Young Adults, and least for Geriatrics.

**Table 1:** Percentage of total population expiring, θ, per age group ${{\text{X}}}_{{\text{i}}}$ where ${\text{i}}$ represents age grouping ${\text{i}}.{\text{e}}., {{\text{X}}}_{{\text{P}}}, {{\text{X}}}_{{\text{J}}}, {{\text{X}}}_{{\text{Y}}}\dots$ calculated as: $\uptheta \left\{{{\text{X}}}_{{\text{i}}}\right\}=\left(\frac{{{\text{N}}}_{{\text{D}}}\left\{{{\text{X}}}_{{\text{i}}}\right\}}{{\text{N}}}\right)*100$. Includes the following statistics: ${\mathbf{N}}_{\mathbf{i}}$ i.e., total sub-population size; ${\mathbf{N}}_{\mathbf{D}\mathbf{i}}$ i.e., total number of deaths per sub-population; ${\varvec{\uptheta}}\left\{{\mathbf{X}}_{\mathbf{i}}\right\}$ i.e., % total population expiring per age group. ${{\text{N}}}_{{\text{D}}}$ (= 284,734) does not equate to N (= 584,734) as the dataset includes subjects alive at point of completion. Thus, $\uptheta \left\{{{\text{X}}}_{{\text{G}}}\right\}$ calculation incorporates remaining subjects.

Age grouping	${{\text{N}}}_{{\text{i}}}$ (sub-population)	${{\text{N}}}_{{\text{Di}}}$ (total no. of deaths, per sub-population)	$\uptheta \left\{{{\text{X}}}_{{\text{i}}}\right\}$ (% total population expiring per age group)
Puppies $\left\{{\mathbf{X}}_{\mathbf{P}}\right\}$	9285	6675	1.14
Juveniles $\left\{{\mathbf{X}}_{\mathbf{J}}\right\}$	13,316	4,770	0.82
Young adults $\left\{{\mathbf{X}}_{\mathbf{Y}}\right\}$	36,433	9,426	1.61
Mature adults $\left\{{\mathbf{X}}_{\mathbf{M}}\right\}$	170,427	49,289	8.43
Senior adults $\left\{{\mathbf{X}}_{\mathbf{S}}\right\}$	198,853	99,729	17.06
Geriatric $\left\{{\mathbf{X}}_{\mathbf{G}}\right\}$	156,420	114,845	70.95
Total	$\mathbf{N}$ = 584,734	${\mathbf{N}}_{\mathbf{D}}$ = 284,734	–

Averaging across all individuals, i.e., pure ($E$) and crossbred ($X$), median survival was estimated at 12.5 years (12.5–12.6, $n$ = 584,734, events = 284,734). However, variation was evident between pure and crossbred individuals (p < 0.001). Median survival for pure breeds was 12.7 years (12.6–12.7, $n$ = 473,681, events = 212,289), whilst median survival for crossbreds was 12.0 years (12.0–12.1, $n$ = 111,053, events = 72,445, HR 1.1, 1.1–1.1, p < 0.001). Probability of purebred and crossbred survival per decimal year are presented in Supplementary Table 1. This table highlights 95% of purebred and crossbred individuals are deceased by 18.3 and 18.2 years, respectively.

**Figure 2:**
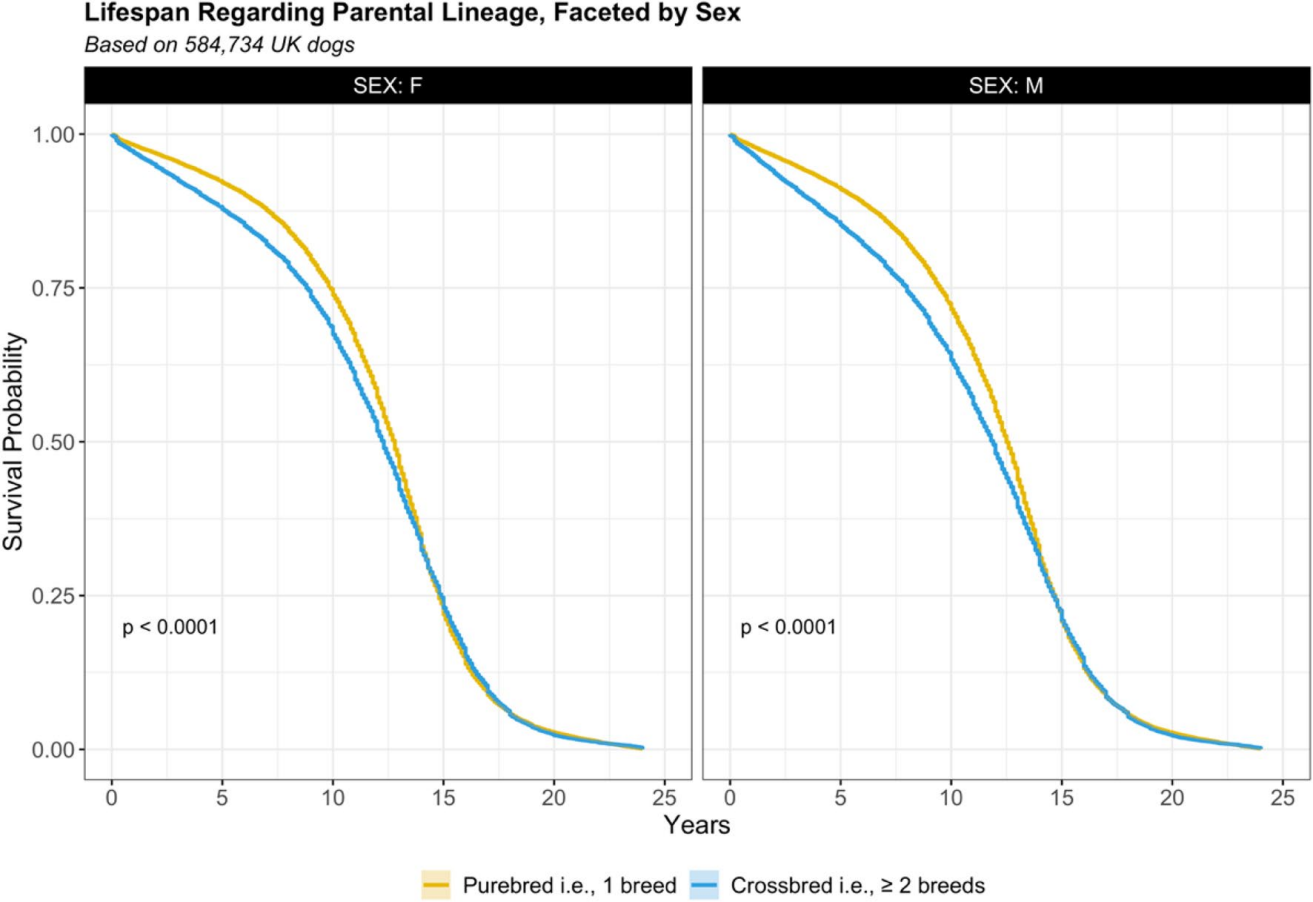
Survival curves of pure (yellow) and cross (blue) bred individuals, faceted by sex. Survival functions based on Kaplan–Meier estimates by log rank test (p-value).

Median survival for any (i.e., $E$ and $X$) female (♀) was 12.7 years (12.6–12.7, $n$ = 283,984, events = 136,910), whilst median survival for any male (♂) was 12.4 years (12.4–12.4, $n$ = 300,750, events = 147,824, HR 1.06, 1.06–1.07, p < 0.001). Probability of female and male survival per decimal year are presented in Supplementary Table 2, which highlight that 95% of both sexes are deceased by 18.3 years of age. Female survival advantage was apparent within both pure and cross bred individuals (x̃_*Ε*_ = 12.8, 12.7–12.8, x̃_*Χ*_ = 12.3, 12.2–12.3), in comparison with their male counterparts (x̃_*Ε*_ = 12.5, 12.5–12.6, x̃_*Χ*_ = 11.9, 11.8–12.0, p < 0.001; Fig. 2).

Longevity estimates for 155 recognised purebreds versus the crossbred group (x̃ = 12.0) are presented in in Supplementary Table 3. In comparison with the crossbred group, 47.1% (${n}_{breeds}$ = 73) of purebreds presented longer median estimates, 25.8% (${n}_{breeds}$ = 40) presented shorter median estimates, and the remaining 27.1% (${n}_{breeds}$ = 42) did not vary significantly from the crossbred group.

**Figure 3:**
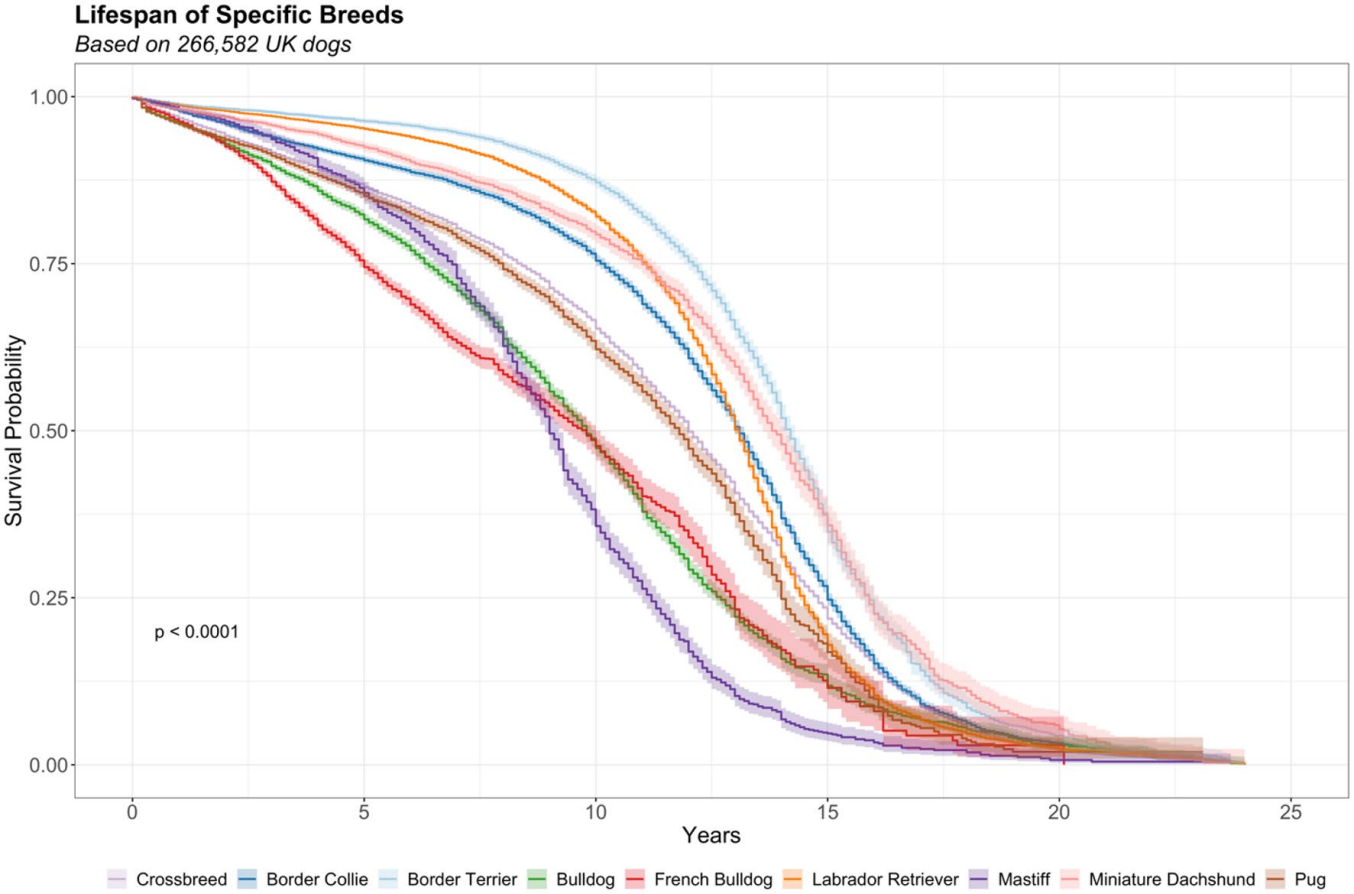
Survival curves for 8 purebreds: Border Collie (dark blue, x̃ = 13.1), Border Terrier (light blue, x̃ = 14.2), Bulldog (green, x̃ = 9.8), French Bulldog (red, x̃ = 9.8), Labrador Retriever (orange, x̃ = 13.1), Mastiff (purple, x̃ = 9.0), Miniature Dachshund (pink, x̃ = 14.0) and Pug (brown, x̃ = 11.6). All purebreds vary significantly from crossbreds (light purple, x̃ = 12.0, p < 0.001). Survival functions based on Kaplan–Meier estimates by log rank test (p-value). Longevity estimates for 155 recognised purebreds versus the crossbred group are presented in in Supplementary Table 3.

Longevity estimates for a subset of purebreds are visualised in Fig. 3. Significant variation in longevity was evident between breeds (p < 0.001): those most at risk from early death included Caucasian Shepherd Dog (x̃ = 5.4, HR 2.4, 1.6–3.7), Presa Canario (x̃ = 7.7, HR 3.0, 2.5–3.7), Cane Corso (x̃ = 8.1, HR 2.4, 2.0–3.0), Mastiff (x̃ = 9.0, HR 2.3, 2.2–2.4), St Bernard (x̃ = 9.3, HR 1.7, 1.5–1.8), Bloodhound (x̃ = 9.3, HR 1.7, 1.3–2.1), Affenpinscher (x̃ = 9.3, HR 1.7, 1.5–1.9), Neapolitan Mastiff (x̃ = 9.3, HR 1.8, 1.6–2.0), Bulldog (x̃ = 9.8, HR 1.8, 1.7–1.8) and French Bulldog (x̃ = 9.8, HR 2.2, 2.1–2.3). Those least at risk from early death included Lancashire Heeler (x̃ = 15.4, HR 0.5, 0.4–0.6), Tibetan Spaniel (x̃ = 15.2, HR 0.5, 0.4–0.5), Shiba Inu (x̃ = 14.6, HR 0.5, 0.4–0.6), Papillon (x̃ = 14.5, HR 0.6, 0.5–0.6), Lakeland Terrier (x̃ = 14.2, HR 0.7, 0.6–0.7), Schipperke (x̃ = 14.2, HR 0.6, 0.4–0.9), Border Terrier (x̃ = 14.2, HR 0.5, 0.5–0.6), Italian Greyhound (x̃ = 14.0, HR 0.7, 0.6–0.9) and Miniature Dachshund (x̃ = 14.0, HR 0.7, 0.6–0.7).

Longevity differed between purebreds of varying size (p < 0.001): median survival for small and medium sized breeds were 12.7 (12.7–12.8, $n$ = 223,222, events = 112,063, HR 0.83, 0.82–0.84) and 12.5 years (12.4–12.6, $n$ = 73,532, events = 35,787, HR 0.88, 0.87–0.89) respectively, with an accelerated time to death for large sized breeds at 11.9 years (11.9–11.9, $n$ = 123,072, events = 61,976). Probability of survival per decimal year for small, medium, and large individuals are presented in Supplementary Table 4: highlighting that 95% of are deceased by 18.1, 18.3 and 17.9 respectively.

Lower survival estimates within large purebreds were apparent within both male and female individuals (x̃_♂_ = 11.8, 11.7–11.8; x̃_♀_ = 12.0, 12.0–12.1), in comparison with their small (x̃_♂_ = 12.7, 12.6–12.7; x̃_♀_ = 12.8, 12.7–12.8) and medium sized counterparts (x̃_♂_ = 12.3, 12.3–12.4; x̃_♀_ = 12.7, 12.6–12.8, p < 0.001). Large males and females presented a 1.28 (1.26–1.29) and 1.17 (1.15–1.18) fold faster time to death, respectively, than the longest living small sized females (p < 0.001). Variation in longevity between the three sizes are more apparent within males (Fig. S2).

**Figure 4:**
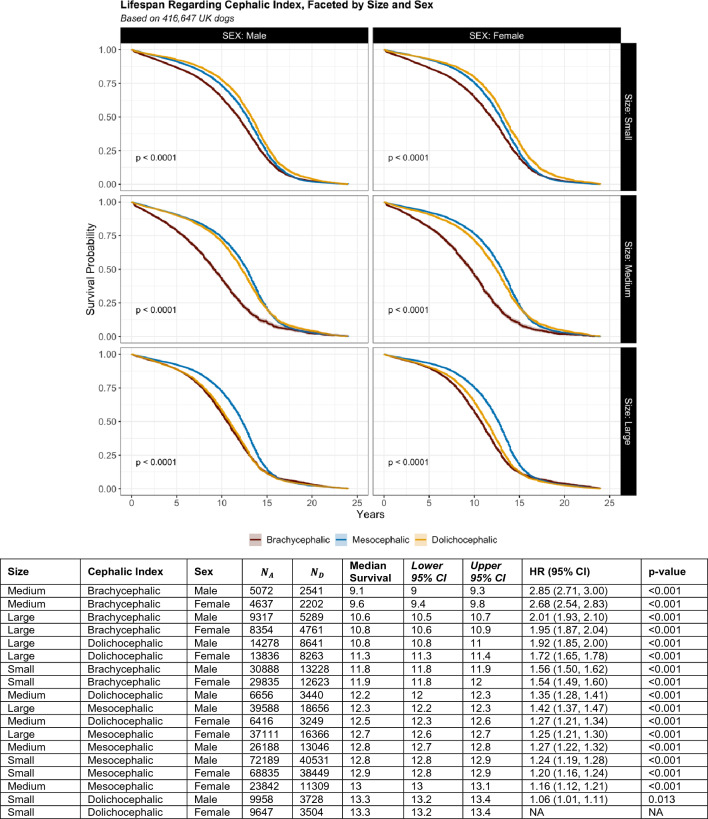
Survival curves of brachycephalic (red), mesocephalic (blue) and dolichocephalic (yellow) purebred individuals, faceted by size and sex, along with associated table. Survival functions based on Kaplan–Meier estimates by log rank test (p-value). Table reports Kaplan–Meier survival estimates and cox proportional hazards regression model outputs for size, by sex. All groupings are compared with small-dolichocephalic-female individuals, as these represent the longest living group. Includes the following statistics: ${\mathbf{N}}_{\mathbf{A}}$ i.e., total number of individuals still alive; ${\mathbf{N}}_{\mathbf{D}}$ i.e., total number of deaths; Median Survival i.e., median age of death; Lower 95% Confidence Interval (CI) and Upper 95% CI; Hazards Ratio (Lower 95% CI and Upper 95% CI) and p-value.

Median survival for mesocephalic purebreds was 12.8 years (12.7–12.8, $n$ = 267,753, events = 138,357), with an accelerated time to death for brachycephalic and dolichocephalic purebreds at 11.2 years (11.2–11.3, $n$ = 88,103, events = 40,644, HR 1.4, 1.4–1.4, p < 0.001) and 12.1 years (12.0–12.1, $n$ = 66,369, events = 33,224, HR 1.1, 1.1–1.1, p < 0.001), respectively. An interaction was evident between cephalic index and size. In comparison with longest living small-dolichocephalic breeds (x̃ = 13.3, 13.2–13.4), brachycephalic-medium and brachycephalic-large sized breeds presented a 2.69 (2.59–2.79, x̃ = 9.4, 9.3–9.5, p < 0.001) and 1.92 (1.87–1.98, x̃ = 10.7, 10.6–10.8, p < 0.001) fold faster time to death, respectively (Fig. S3). However, a further interaction was noted, between cephalic index, size, and sex (Fig. 4). Similar to previous findings, in comparison with longest living small-dolichocephalic-female breeds (x̃ = 13.3, 13.2–13.4), medium-brachycephalic male and female breeds presented a 2.85 (2.71–2.3, x̃ = 9.1, 9.0–9.3, p < 0.001) and 2.69 (2.54–2.83, x̃ = 9.6, 9.4–9.8, p < 0.001) fold faster time to death, respectively. Furthermore, variation in longevity between the three cephalic indices were most apparent within medium sized dogs.

### Phylogenetic analyses

**Figure 5:**
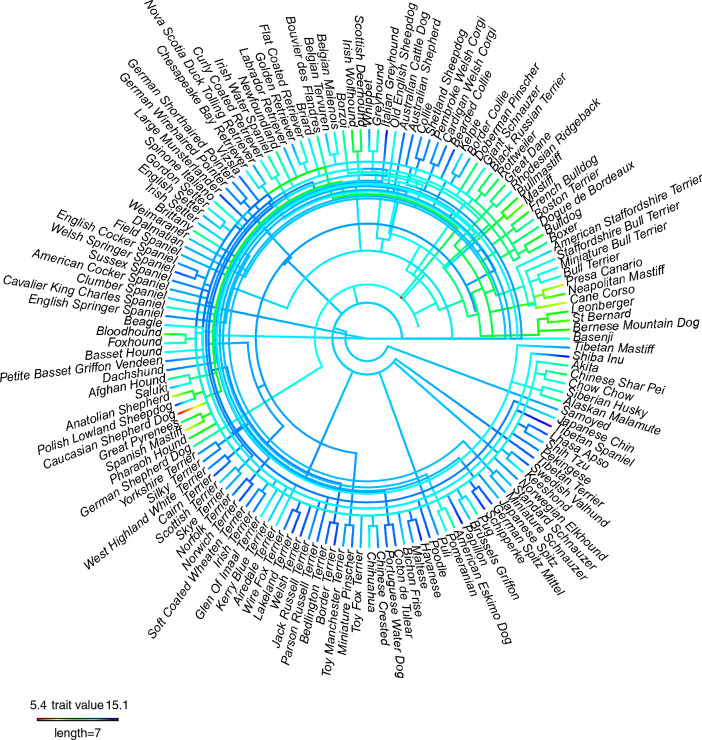
Circular phylogeny representing ancestor‐to‐descendant breed relationship, along with median lifespan (see Fig. S4 for flat phylogeny). Median lifespan for 148 purebreds, of which we had lifespan data, and could be assigned to tips of existing phylogenies^113,114^. Hotter colours represent lower median lifespans. The phylogenetic signal across the entire breed tree was very strong (Pagel’s Lambda = 0.808), suggesting that median lifespan was strongly affected by the evolutionary history of dog breeds.

Pagel’s lambda value of 0.808 was obtained, suggesting that median lifespan exhibited a strong phylogenetic signal across the dog breeds included in these analyses. Distribution of median lifespan was strongly related to evolutionary history: with breeds within clades exhibiting closer lifespans than would be expected by chance (Fig. 5, see Fig. S4 for flat phylogeny).

**Figure 6:**
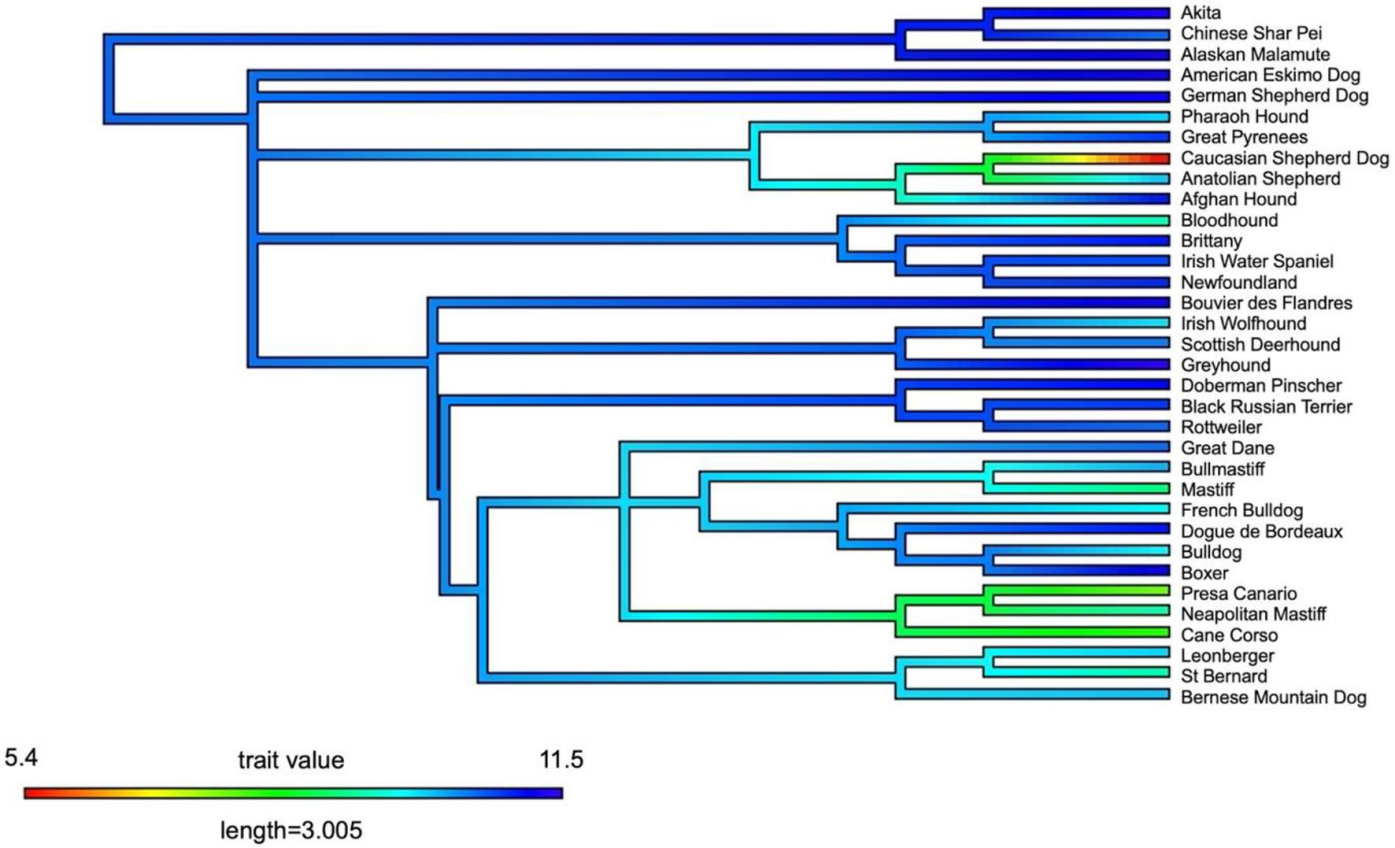
Median lifespan of the lowest quartile of breeds plotted onto the breed phylogeny. Hotter colours represent lower median lifespans.

Figure 6 presents the median lifespans of the lowest quartile of breeds plotted onto the breed phylogeny. Clusters with lower median lifespans are localised within three clades: the first including breeds such as the Caucasian Shepherd Dog, the second containing the Mastiffs and Bulldogs, and the third including the Presa Canario, Neapolitan Mastiff and Cane Corso.

## Discussion

Our study investigated variation in longevity estimates for dogs located within the UK, based on parental lineage, breed, body size, sex, cephalic index, and phylogeny. Our results partition heterogeneity of survival estimates across these populations: identifying groups most at risk from early death. More specifically, we provide longevity estimates for: (1) pure (PL = 1 breed) versus crossbred (PL ≥ 2 breeds) groupings, providing evidence to inform discussions regarding pedigree health; (2) 155 purebreds, representing a large range of morphological, behavioural, and physiological phenotypes; (3) varying body size, providing support for a negative correlation between size and longevity^3,35,47,52,59^; (4) sex, reinforcing documented female survival advantage within mammalian species^64^; and (5) cephalic index, expanding upon the brachycephalic health and welfare debate, regarding problems associated with conformation^66,67^. Furthermore, this is one of the first studies to map longevity estimates against evolutionary history (via domestication and associated artificial selection), providing evidence that canine ancestral lineage is associated with breed lifespan (however, see ^41,59,70^).

Breed specific estimates of survival are not only informative to veterinarians and researchers, but also to current and prospective owners, looking to fully understand their future responsibilities, and potential duration of dog-owner relationship. Whilst this study provides evidence to inform discussions around canine pedigree health, it is important to note that unbiased lifespan data necessarily requires the inclusion of live dogs whose cause of death is yet unknown, and furthermore, for those deceased—reason for death is not included within our dataset. Death may be due to euthanasia (based on physiological or behavioural concerns), trauma, disease, or natural causes. Thus, we cannot identify direct risk factors for early death. Instead, by comparing survivorship across parental lineage, breed, body size, sex, cephalic index, and phylogeny, we can identify groupings and/or lineages that demand further consideration.

Previous studies have reported significantly lower life expectancies for purebreds, in comparison with their crossbred counterparts^34,36,40,71^, leading to widespread belief that crossbred dogs are substantially healthier than purebreds^72,73^. We found that 47.1% of purebreds present a greater median survival estimate than crossbreds, only 25.8% present a shorter life expectancy than crossbreds, and 27.1% did not vary significantly. Consequently, our results are not in agreement with previous findings. However, due to methodological limitations, we do not reject the existence of hybrid vigour within the population. Within our data, purebreds and crossbreds are evaluated as a binary variable. This may be problematic as crossbreds are heterogenous: presenting differing degrees of between-breed genetic diversity. For example, the crossbred group ranges from individuals produced from a complex line of unknown hybrids to purebred hybrids, often labelled as ‘designer crossbreeds’ e.g., ‘Labradoodle’, Labrador Retriever x Poodle or ‘Jack-Chi’, Jack Russell Terrier x Chihuahua. Within purebred hybrids, while the first filial (F1) generation may benefit from hybrid vigor^43^ and display fewer inbreeding depression effects than their ancestral purebreds^63,74,75^, subsequent generations i.e., *F2, F3…Fx*, are likely to return a higher inbreeding co-efficient. Thus, they lose the health benefits from the initial purebred hybrid cross^42^. As we do not have access to detailed information regarding individual lineage, we cannot assess the presence of hybrid vigor, or inbreeding depression, within a quantitative context. However, given the growing popularity of specific 'designer cross’ breeding programs^76,77^, along with the proposed strategy to abandon or ‘outcross’ purebred populations significantly affected by inherited disorders and/or extreme characteristics^20,28,78,79^, it is important that future studies partition heterogeneity of survival estimates amongst mixed (PL > 2 breeds or unknown), known crossbred (PL = 2 breeds e.g., 'designer cross’) and ancestral purebred lineages. Furthermore, as mixed breeds have been reported to live 1.2 years longer than size-matched purebred dogs, and individual breeding level has been negatively associated with juvenile survival and adult lifespan^28^, future studies should consider incorporating individual-level phenotype i.e., lifespan, body size and PL, from genotyped dogs, to disentangle the effect of breed and body size upon longevity. Such research would help establishing the true extent of any suggested hybrid health risks and/or benefits by improving power for detecting subtler effects of inbreeding, as well as specific genetic variants that may affect lifespan.

This study evaluated 584,734 pure and crossbred dogs (including 284,734 deceased), from 18 organisations, including rehoming and welfare organisations, breed registries, pet insurance companies, veterinary corporations, and university based veterinary archives. The greater effective sample size of the aggregated data, has the potential to improve model estimates^80^, by providing a more comprehensive and representative picture of the study population. However, it is not without its shortcomings. Specific sources may introduce representation bias, providing incomplete information which impacts estimates, or reporting termination/death related statistics that differ from the general canine population. Pet insurance data have previously been used for assessing longevity estimates for pet populations^37,81,82^. Despite this fact, their value has been affected by the following biases: older animals are often uninsured; some health conditions are excluded (especially where repeated); presence of financial thresholds on claims; and some policies providing age-limited life cover. Additionally, not all pets will be represented in these data since not all owners are able, or choose, to insure their pet. This can lead to some breeds being under-represented within insurance data: especially those of high disease risk, such as brachycephalic breeds, due to the elevated cost of insurance^83^. Previous studies have also reported owners of purebreds are more likely to financially invest in their dogs, showing a greater willingness to pursue more extensive veterinary treatment at referral hospitals, than owners of mixed breed dogs^84–86^. This would result in a greater representation of purebred dogs within veterinary datasets (as seen in the present study). Furthermore, the growing popularity of certain breeds may result in an over overrepresentation of younger individuals within the data, which increases the risk of underestimating their lifespan^87^. Finally, due to the nature of breed registries, it is likely that these data are biased towards pedigree or purebred dogs—despite crossbreds being recorded within ‘separate’/alternate registries^88^. With the aim of mitigating some aforementioned biases, we incorporated rehoming and welfare charity datasets, where crossbred dogs have been reported to represent 44–87.8% of the UK rehoming population^68,89^. However, we recommend future researchers address biases by checking for breed survival differences between sources (not available here due to restrictions stipulated within data sharing agreements with project participants), and addressing missing data via imputation or other statistical techniques^90,91^.

Despite previous research reporting a survival advantage for neutered animals^3,65,92,93^ due to data limitations, we had to omit neuter status from this analysis. Neuter status is a time-dependent variable likely to have a non-linear relationship with age, both with regards to probability of the procedure having been carried out, and the probability of attaining the updated status within multiple data sources^94,95^. As such, the available data was deemed unreliable and excluded from further analysis.

Whilst these findings provide valuable insight into breed longevity, it is important to acknowledge that purebred dog populations and, thus, their population management are influenced by environment and location. Consequently, these results are representative of the UK canine population only, and should not be used to make assessments within other regions/countries. For example, within certain countries, breed registries have adopted independent policies regarding the importation, breeding, and registration of foreign dogs. Some registries effectively disallow breeding of imported dogs and prevent recognition of the resulting puppies—whilst others allow for the controlled importation and breeding of ‘foreign’ animals. The latter policy introduces genetic diversity: potentially impacting the overall longevity of their canine population^96^. Furthermore, variation in public attitude towards dogs (owned or free roaming) and responsible dog ownership practices, will alter population management approaches^97^. Consequently, further research is needed to explore the impact of regional dog population management practices and breed registry policies, upon the lifespan of local purebred dogs.

Variations in popularity of dog breeds are often evident as large and impulsive fluctuations, that are usually considered the hallmark of fashions and fads^98,99^. An acute increase in breed demand and impulse buying^100,101^ have resulted in puppies becoming lucrative commodities in an industry driven by profitability, often at the expense of canine welfare^102,103^. Recent studies have reported an increase in rates of dogs suffering from physiological and psychological issues caused by inappropriate breeding practices, pre-purchase handling, early life environment and husbandry^102,104–107^. Much of this supply may originate from large-scale, industrial-style operations, in which breeders may attempt to lower overheads through poor husbandry and deficient hygiene standards: operations that are colloquially dubbed ‘puppy farms’^102,104^. However, puppy welfare issues extend to some regulated and small-scale breeders due to limited regulation and/or the practice of inbreeding to ‘fix’ characteristics associated with breed standards. This can lead to increased risk of hereditary pathology and exaggerated physical characteristics^20^ including orthopaedic disorders^29,108^, skin disease^29,109^, aural disease^29,46,110^, ocular disease^111,112^, and breathing difficulties^105,106^. Gaps in regulation and the inability to reliably quantify supply sources have significant implications on the ‘dog market’, including the ethics of breeding practices. It is therefore imperative that future discussions regarding optimal canine breeding practices are open and multidisciplinary. As such, we hope these findings empower stakeholders, providing evidence required to vocalise opinions and collectively consider the impact of (1) biology e.g., genotype^113^, phenotype^28^, conformation^66,114^, or physiology^115^, and (2) non-biological factors e.g., owner demographics^116,117^, management styles^31,118^, or breed function^5^, upon risk of early death within the UK canine population.

## Methods

### Data sources, cleaning and subsetting

Dogs Trust data were combined with datasets sourced from 17 external project participants. Data sources included breed registries (45.0%), veterinary corporations (26.5%), pet insurance companies (17.1%), animal welfare charities (5.9%), and academic institutions (5.5%). Project participants who provided data to this project include: Battersea Dogs and Cats Home; Blue Cross; SSPCA; Raystede; Wood Green, The Animals Charity; Edinburgh Dog and Cat Home; PDSA; Mayhew; The Insurance Emporium (The Equine and Livestock Insurance Company Limited); NCI Insurance; Cardif Pinnacle; Agria Pet Insurance Ltd; Direct Line; Medivet; Vets4Pets; Savsnet (Small Animal Veterinary Surveillance Network, University of Liverpool); and The Kennel Club (UK).

Data requested included: breed (free text), crossbred (Y/N/unknown), sex (M/F/unknown), date of birth (DOB; MM/YYYY), postcode area (i.e., first one or two characters), first three characters of dog name (common, not pedigree name), last six characters of microchip number, last six characters of additional microchip number (if more than one known), status (alive/dead) and termination date (death or end of policy due to death; DD/MM/YY). Not all canine-centric variables were sent by project participants and no data were collected regarding owner unique identifiers. Longevity (age of dog in decimal years) reflects the period between DOB and termination date (if deceased), or date data were received (if assumed alive).

Data cleaning included the removal of non-canids, classifying all individuals into (1) The UK Kennel Club (KC)^119^ and/or Fédération Cynologique Internationale (FCI)^120^ recognised ‘purebred’ breeds (PL = 1 breed), or (2) ‘crossbred’ breeds (PL ≥ 2 breeds). Breeds within the dataset may be present in one or both KC and FCI lists. Those that are not included in either but appear in the data were used as outgroups for phylogenetic analyses e.g., American Hairless Terrier, Apennine Wolf, Pastore della Lessinia e del Lagorai, Pastor della Sila and Wolf & Golden Jackal (Supplementary Table S5). Forty-four KC and/or FCI breeds were reclassified to match nodes, i.e., breeds, within existing canine phylogenies^121,122^. For example, ‘American Akita’ and ‘Japanese Akita Inu’ were collapsed into ‘Akita’, which is present in both KC^119^ and FCI^120^ breed lists. These changes (listed in Supplementary Table S5; under ‘*Phylogeny Reclassification’*), were necessary due to breed inconsistency across the data sources and were instituted via majority agreement by canine behaviour and research experts at Dogs Trust.

Body size classifications: small, medium, and large, were obtained from KC^119^ and FCI^120^ grey literature. Breed average ratio between the width and length of skull i.e., cephalic index: brachycephalic (flat-faced), mesocephalic (medium proportions) or dolichocephalic (long-faced), were sourced from O’Neill et al., 2020^123^. Breeds present within our data, but omitted from the above lists, were assigned body size and cephalic index classification via majority agreement by a group of canine behaviour and research experts at Dogs Trust (Supplementary Table S5). Body size and cephalic index data are only available for purebred individuals, due to phenotypic variation within crossbreds.

Month and year of birth were routinely recorded by rehoming and welfare centres, breed registries, veterinary corporations, and pet insurance companies. Unfortunately, the specific date was less commonly reported. Consequently, all age estimates were based on ‘MM/YYYY’ data. Individuals were grouped into one of six age categories: ‘Puppies’ $\left({X}_{P}\right)$ aged 0 to < 6 months, ‘Juveniles’ $\left({X}_{J}\right)$ aged 6 to < 12 months, ‘Young Adults’ $\left({X}_{Y}\right)$ aged 12 to < 24 months, ‘Mature Adults’ $\left({X}_{M}\right)$ aged 2 to < 7 years, ‘Senior’ $\left({X}_{S}\right)$ aged 7 to < 12 years and ‘Geriatric’ $\left({X}_{G}\right)$ aged ≥ 12. These categories were developed to capture age-related developmental trajectories for most dog breeds^124^.

As data were obtained from multiple sources, duplication of individuals was probable. Deduplication consisted of a four-phase process outlined in Supplementary Note S1. For the purposes of this study, deduplicated data were then subset to rows where the following variables were complete: crossbred (Y/N); status (alive/dead), breed (n = 155), sex (M/F) and age (decimal years). To remove improbably aged dogs, DOB was restricted to ≤ 24.0 years, based on previously reported maximum canine longevity^3^. Each group (e.g., Affenpinscher or Yorkshire Terrier) within an independent variable (e.g., breed), were only included in analysis, if an adequate number of individuals (and events) were available i.e., > 20 alive and > 20 deceased individuals (Clark et al. 2003). Resulting alive $\left({N}_{A}\right)$, deceased $\left({N}_{D}\right)$ and total $\left(N\right)$ sample sizes for each independent variable are listed in Table 2, and associated samples sizes per group within each independent variable, are listed in Supplementary Tables S6–S11.

**Table 2:** Samples sizes regarding alive $\left({\mathbf{N}}_{\mathbf{A}}\right)$, deceased $\left({\mathbf{N}}_{\mathbf{D}}\right)$ and total $\left(\mathbf{N}\right)$ individuals, per independent variable (post censoring).

Independent variable	Sample sizes		
	${{\text{N}}}_{{\text{A}}}$	${{\text{N}}}_{{\text{D}}}$	${\text{N}}$
Breed Parental lineage Sex Age group	300,000	284,734	584,734
Body size	210,000	209,826	419,826
Cephalic index	210,000	212,225	422,225

### Statistical analyses

All analyses were conducted using the statistical programming software R version 4.0.4 (2021-02-15)^125^. As the dataset included a large number of subjects alive at point of completion i.e., not all subjects died during the study, this produces censored values. Having a large number of censored values decreases the equivalent number of subjects exposed (at risk) at later times, making survival estimates less reliable than they would be for the same number of subjects with less censoring^126^. Ignoring censored values, or simply equating their observed survival time with the unobserved total survival time (i.e., assuming all censored survival times occur immediately after their censoring times), would bias the results^127^. Fortunately, censoring is common within these data, such that specific statistical methods have been developed for appropriate analysis. Maximum Likelihood Estimation is the most notable method for analysing censored data. This method adjusts for whether the subjects observed were censored or not. It also utilizes all the information available. The statistical approaches that fall within the likelihood method include those used within this study^128^.

Kaplan–Meier survival curves^129,130^ were estimated per group, within each independent variable i.e., parental lineage, breed, size, sex, and cephalic index, using the *surviminer* package^131^. Median survival estimates are presented in place of mean values, as extreme values from non-normally distributed longevity distributions provide unreliable estimates^87^. Upper and lower 95% confidence intervals immediately follow median survival estimates or hazard ratios, unless otherwise stated. To test for treatment differences between survival curves, pairwise log-rank tests were conducted. Differences in proportional risks of mortality between categories were tested using a Cox proportional hazards model.

Percentage of total population expiring (Table 1), θ, per age group ${X}_{i}$ where $i$ represents age grouping $i.e., {X}_{P}, {X}_{J}, {X}_{Y}\dots$ were calculated as:

$$\theta \left\{{X}_{i}\right\}=\left(\frac{{N}_{D}\left\{{X}_{i}\right\}}{N}\right)*100$$

As dataset includes subjects alive at point of completion (${N}_{A}$), total number of deaths (${N}_{D}$) does not equate with total population ($N$). This produces ‘censored values’ (discussed above)^126^. Thus, $\theta \left\{{X}_{G}\right\}$ calculation incorporates remaining subjects, as ${N}_{D}$ would eventually equate to $N$, as measurements continued:

$$\theta \left\{{X}_{G}\right\}=N-\left(\sum {N}_{D} \left\{{X}_{P}{,X}_{J},{X}_{Y},{{X}_{M},X}_{S}\right\}\right)*100$$

Pagel’s Lambda^132^ was calculated using the *phylosig* function from the *phytools*^133^ package, with the aim of determining whether the median lifespan of the purebreds included in the analysis held a phylogenetic signal. The presence of a phylogenetic signal for a given trait suggests clustering of that trait on the phylogeny. Lambda is a metric that quantifies the level to which the similarity of a continuous trait is due to evolutionary history and can take any value within the range 0 to 1. A signal strength of 0 means that there is no phylogenetic signal, i.e., correlations between values of the trait do not reflect the relatedness of the tips of the phylogenetic tree. A signal strength of 1 suggests that the values of the tips are highly dependent upon evolutionary history. The phylogeny of domestic dogs was based on that of Parker et al., (2017)^121^ and supplemented with extra breeds from Talenti et al., (2018)^122^. In total 148 purebreds were included, for which we had lifespan data. All branch lengths were set to equal 1 before analysis, as the original tree(s) did not include branch length information. To visualise the variation in median lifespan across the phylogeny of purebreds, specifically those clades in which reduction in median lifespan clustered, we used the *contMap* function within *phytools*^133^. For visualisation we restricted this to purebreds within the lower quartile of median lifespans, i.e., the 34 purebreds with the lowest median lifespan.

### Ethics approval and consent to participate

Ethical approval for this study was granted by Dogs Trust Ethical Review Board (Reference Number: ERB038). All methods were performed in accordance with the relevant guidelines and regulations.

### Supplementary Information

Supplementary Figure 1.

Supplementary Figure 2.

Supplementary Figure 3.

Supplementary Figure 4.

Supplementary Tables 6-11.

Supplementary Table 1.

Supplementary Table 2.

Supplementary Table 3.

Supplementary Table 4.

Supplementary Table 5.

Supplementary Note S1.

## Data Availability

Datasets generated and analysed during the current study are not publicly available due to restrictions stipulated within data sharing agreements with project participants. However, the corresponding author will consider facilitating collaborative discussions with all parties involved, on reasonable request.
