# Author Correction: Longevity of companion dog breeds: those at risk from early death

**DOI:** 10.1038/s41598-024-59331-w

**Published:** 2024-04-16

**Authors:** Kirsten M. McMillan, Jon Bielby, Carys L. Williams, Melissa M. Upjohn, Rachel A. Casey, Robert M. Christley

**Affiliations:** 1 Dogs Trust, London, UK; 2 Liverpool John Moores University, Liverpool, UK

Correction to: *Scientific Reports* 10.1038/s41598-023-50458-w, published online 01 February 2024

The original version of this Article contained an error in the legend of Figure 3.

“**Figure 3**. Survival curves for 8 purebreds: Border Collie (dark blue, x̃ = 13.1), Border Terrier (light blue, x̃ = 14.2), Bulldog (green, x̃ = 9.8), French Bulldog (red, x̃ = 9.8), Labrador Retriever (orange, x̃ = 13.1), Mastiff (purple, x̃ = 9.0), Miniature Dachshund (pink, x̃ = 12.2) and Pug (brown, x̃ = 11.6).”

now reads:

“**Figure 3**. Survival curves for 8 purebreds: Border Collie (dark blue, x̃ = 13.1), Border Terrier (light blue, x̃ = 14.2), Bulldog (green, x̃ = 9.8), French Bulldog (red, x̃ = 9.8), Labrador Retriever (orange, x̃ = 13.1), Mastiff (purple, x̃ = 9.0), Miniature Dachshund (pink, x̃ = 14.0) and Pug (brown, x̃ = 11.6).”

The original Article has been corrected.

