# Supplementary figures and images for: Longevity of companion dog breeds: those at risk from early death
Source: Sci Rep. 2024 Feb 1;14:531. doi: 10.1038/s41598-023-50458-w (PMC10834484; doi:10.1038/s41598-023-50458-w)

***Figure S1:*** *Frequency of 155 breeds* within purebred dataset *( = 473,423; % representation listed beside bar).*

*
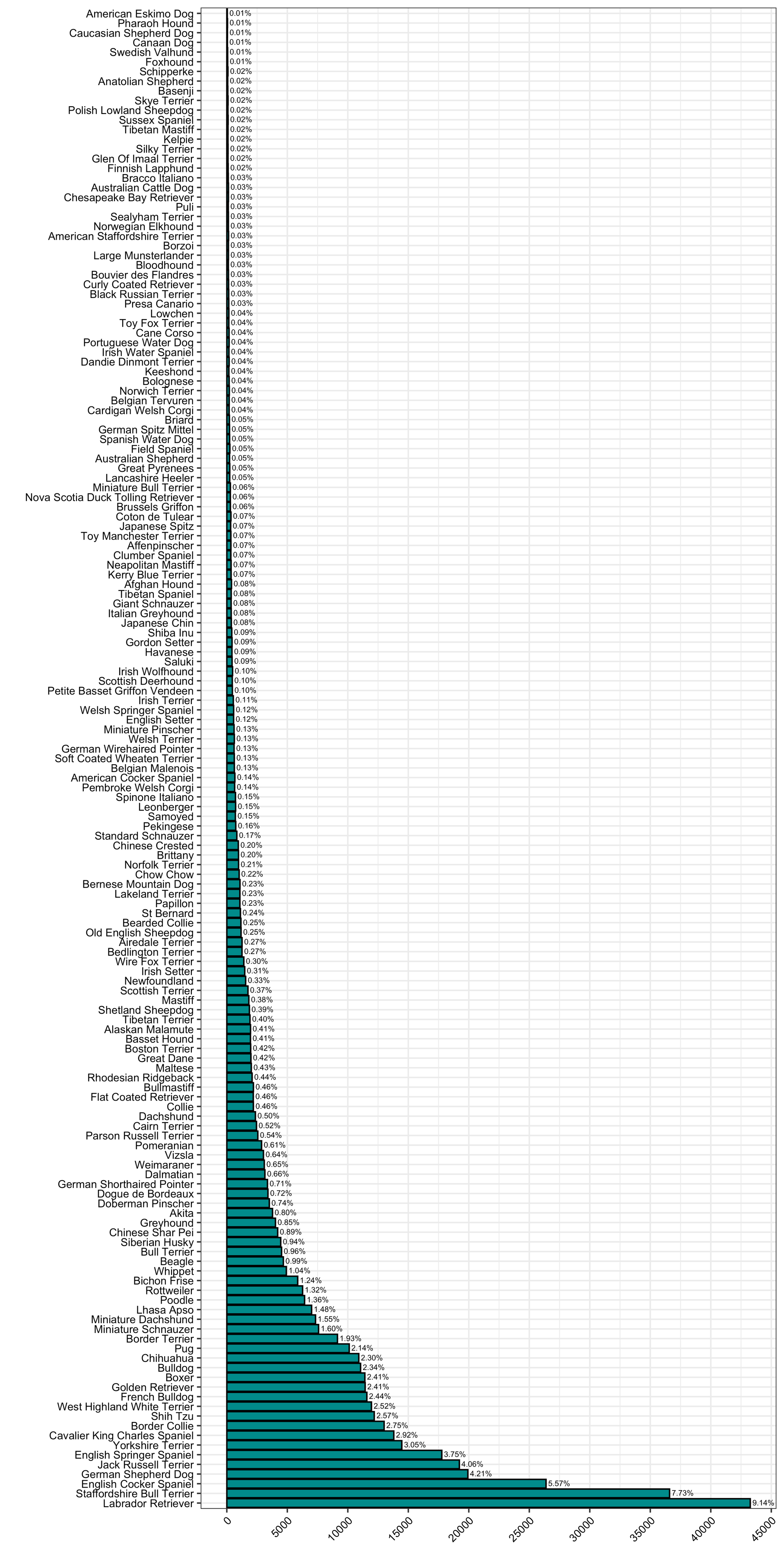
*

Supplement: Supplementary file 1 — Supplementary Figure 1. [file 41598_2023_50458_MOESM1_ESM.docx]
