# Supplementary material for: Longevity of companion dog breeds: those at risk from early death
Source: Sci Rep. 2024 Feb 1;14:531. doi: 10.1038/s41598-023-50458-w (PMC10834484; doi:10.1038/s41598-023-50458-w)
Supplement: Supplementary file 2 — Supplementary Figure 2. [file 41598_2023_50458_MOESM2_ESM.docx]

**Figure S2:** Survival curves of large (purple), medium (pink) and small (green) purebred individuals, faceted by sex, along with associated table. Survival functions based on Kaplan-Meier estimates by log rank test (p-value). Variation in longevity between the three body sizes are more apparent within males. Table reports Kaplan-Meier survival estimates and cox proportional hazards regression model outputs for body size, by sex. All groupings are compared with Small-Female individuals, as these represent the longest living group. Includes the following statistics: $\boldsymbol{N}_{\boldsymbol{A}}$ i.e., total number of individuals still alive; $\boldsymbol{N}_{\boldsymbol{D}}$ i.e., total number of deaths; **Median Survival** i.e., median age of death; **Lower 95% Confidence Interval (CI)** and **Upper 95% CI**; **Hazards Ratio** (Lower 95% CI and Upper 95% CI) and **p-value**.

*
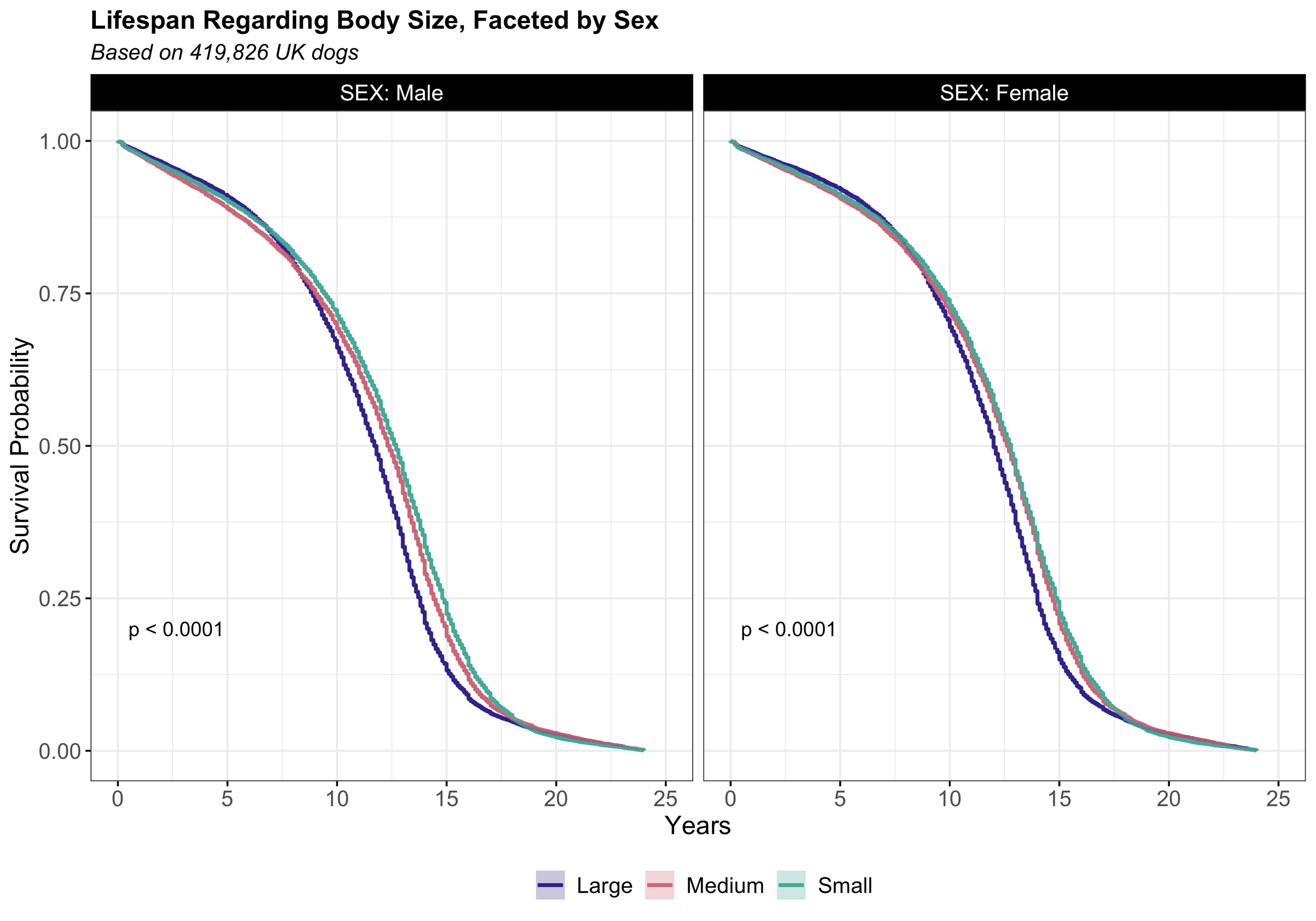
*

| **Body Size** | **Sex** | $\boldsymbol{N}_{\boldsymbol{A}}$ | $\boldsymbol{N}_{\boldsymbol{D}}$ | **Median Survival** | ***Lower 95% CI*** | ***Upper 95% CI*** | **HR (95% CI)** | **p-value** |
| --- | --- | --- | --- | --- | --- | --- | --- | --- |
| Large | Male | 63647 | 32586 | 11.8 | 11.7 | 11.8 | 1.28 (1.26, 1.29) | <0.001 |
| Large | Female | 59531 | 29390 | 12.0 | 12.0 | 12.1 | 1.17 (1.15, 1.18) | <0.001 |
| Medium | Male | 38618 | 19027 | 12.3 | 12.3 | 12.4 | 1.11 (1.09, 1.13) | <0.001 |
| Medium | Female | 35290 | 16760 | 12.7 | 12.6 | 12.8 | 1.02 (1.00, 1.04) | 0.037 |
| Small | Male | 113560 | 57487 | 12.7 | 12.6 | 12.7 | 1.03 (1.02, 1.04) | <0.001 |
| Small | Female | 109180 | 54576 | 12.8 | 12.7 | 12.8 | NA | NA |
