# Supplementary material for: Longevity of companion dog breeds: those at risk from early death
Source: Sci Rep. 2024 Feb 1;14:531. doi: 10.1038/s41598-023-50458-w (PMC10834484; doi:10.1038/s41598-023-50458-w)
Supplement: Supplementary file 3 — Supplementary Figure 3. [file 41598_2023_50458_MOESM3_ESM.pdf]

Figure S3: *Survival curves of brachycephalic (red), mesocephalic (blue) and dolichocephalic (yellow) purebred individuals, faceted by body size, along with associated table. Survival functions based on Kaplan-Meier estimates by log rank test (p-value). Variation in longevity between the three cephalic indices are more apparent within medium sized dogs. Table reports Kaplan-Meier survival estimates and cox proportional hazards regression model outputs for body size, by sex. All groupings are compared with Small-Dolichocephalic individuals, as these represent the longest living group. Includes the following statistics:* N_A *i.e., total number of individuals still alive;* N_D *i.e., total number of deaths;* ***Median Survival*** *i.e., median age of death;* ***Lower 95% Confidence Interval (CI)*** *and* ***Upper 95% CI***; ***Hazards Ratio*** *(Lower 95% CI and Upper 95% CI) and* ***p-value***.

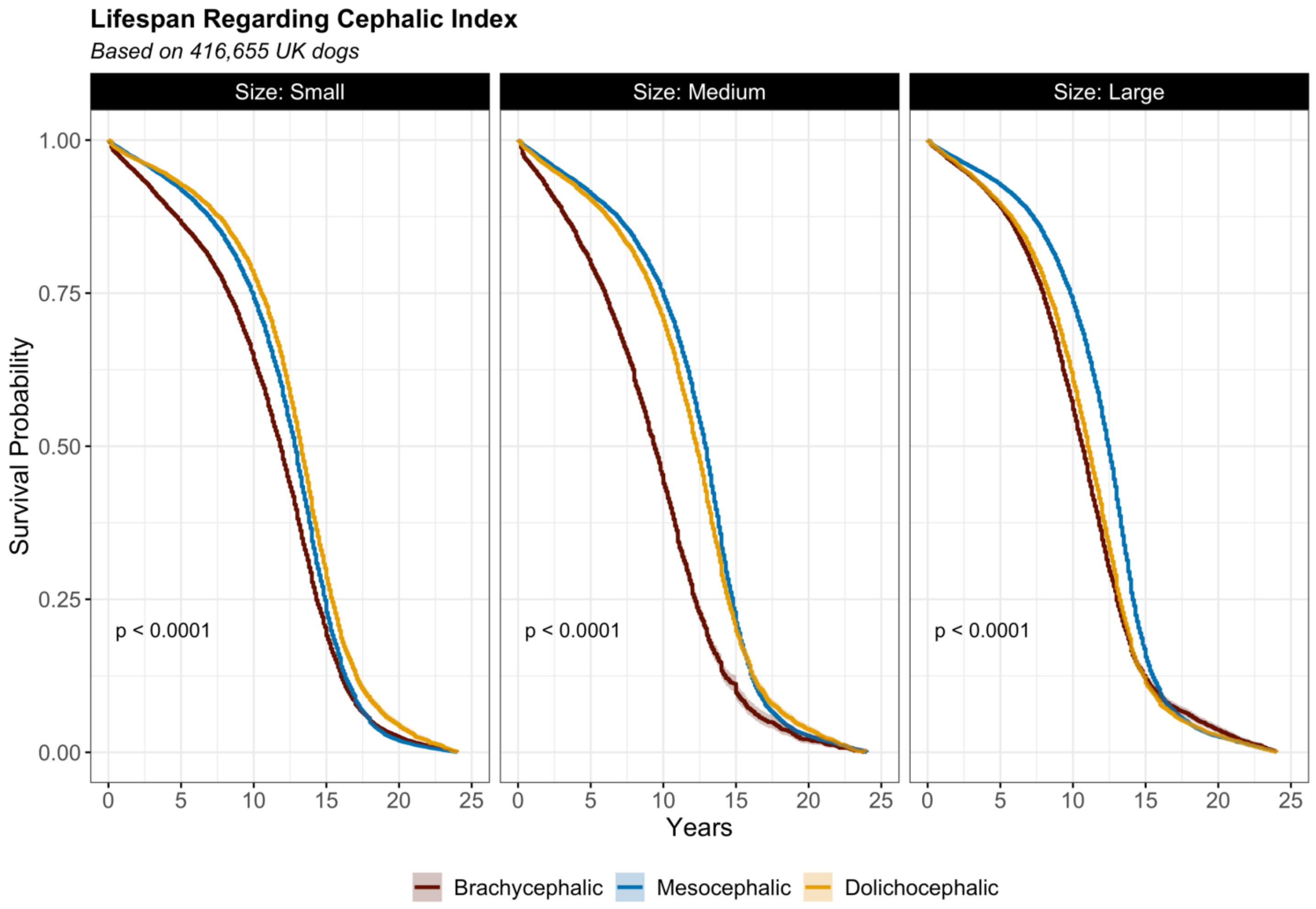

Size	Cephalic Index	N_A	N_D	Median Survival	*Lower 95% CI*	*Upper 95% CI*	HR (95% CI)	p-value
Medium	Brachycephalic	9709	4743	9.4	9.3	9.5	2.69 (2.59, 2.79)	<0.001
Large	Brachycephalic	17671	10050	10.7	10.6	10.8	1.92 (1.87, 1.98)	<0.001
Large	Dolichocephalic	28114	16904	11.1	11.0	11.2	1.76 (1.71, 1.81)	<0.001
Small	Brachycephalic	60723	25851	11.8	11.8	11.9	1.51 (1.47, 1.55)	<0.001
Medium	Dolichocephalic	13072	6689	12.3	12.2	12.4	1.27 (1.23, 1.31)	<0.001
Large	Mesocephalic	76699	35022	12.5	12.4	12.5	1.30 (1.27, 1.33)	<0.001
Small	Mesocephalic	141024	78980	12.8	12.8	12.9	1.18 (1.16, 1.21)	<0.001
Medium	Mesocephalic	50030	24355	12.9	12.8	13.0	1.18 (1.15, 1.21)	<0.001
Small	Dolichocephalic	19605	7232	13.3	13.2	13.4	NA	NA
