# Supplementary material for: Longevity of companion dog breeds: those at risk from early death
Source: Sci Rep. 2024 Feb 1;14:531. doi: 10.1038/s41598-023-50458-w (PMC10834484; doi:10.1038/s41598-023-50458-w)

***Figure S4:*** *Flat* *phylogeny representing ancestor‐to‐descendant breed relationship, along with median lifespan (see Fig 5 for circular representation). Median lifespan for 148 purebreds, of which we had lifespan data, and could be assigned to tips of existing phylogenies^85,86^. Hotter colours represent lower median lifespans. The phylogenetic signal across the entire breed tree was very strong (Pagel’s Lambda = 0.808), suggesting that median lifespan was strongly affected by the evolutionary history of dog breeds.*


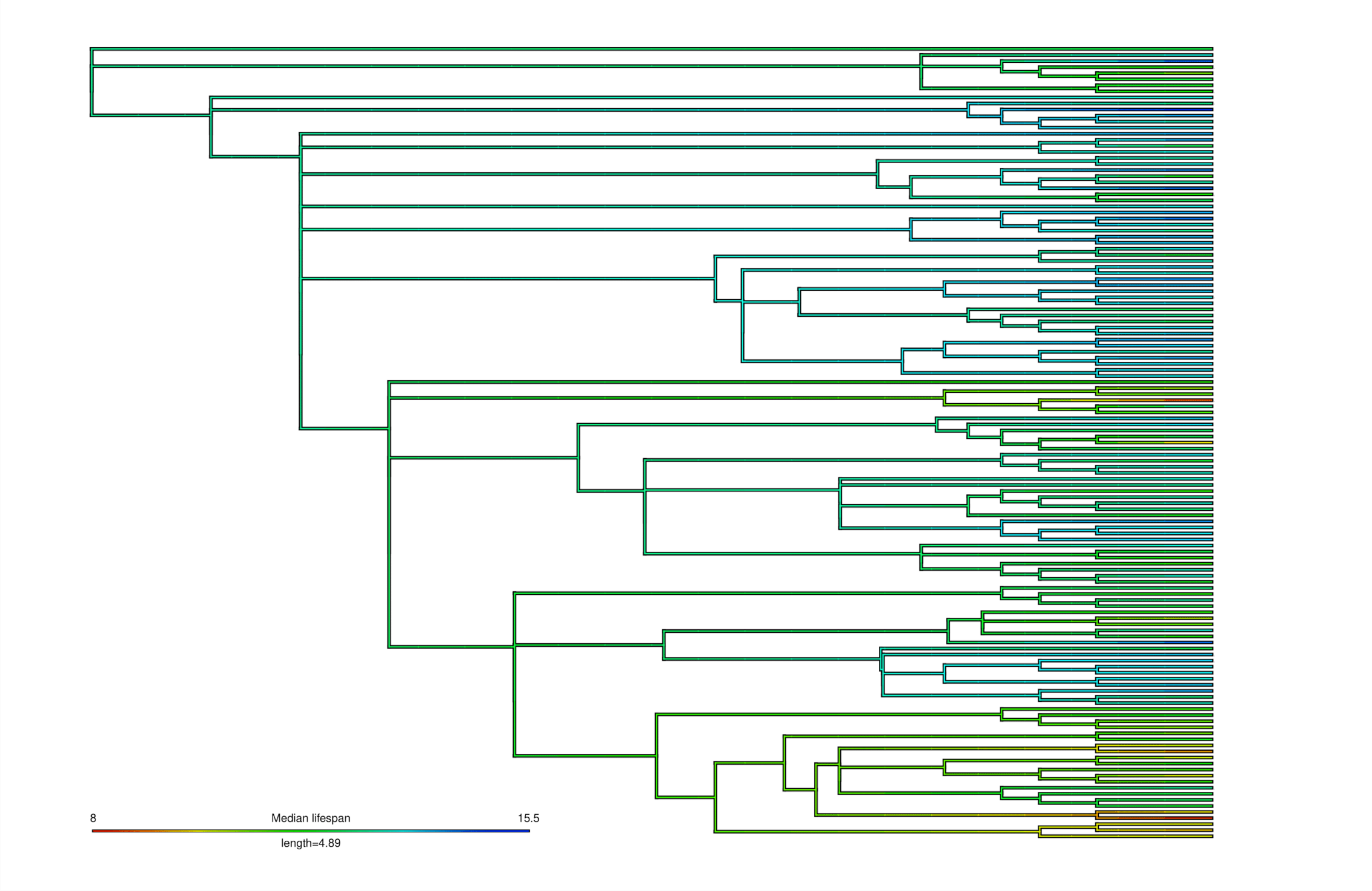

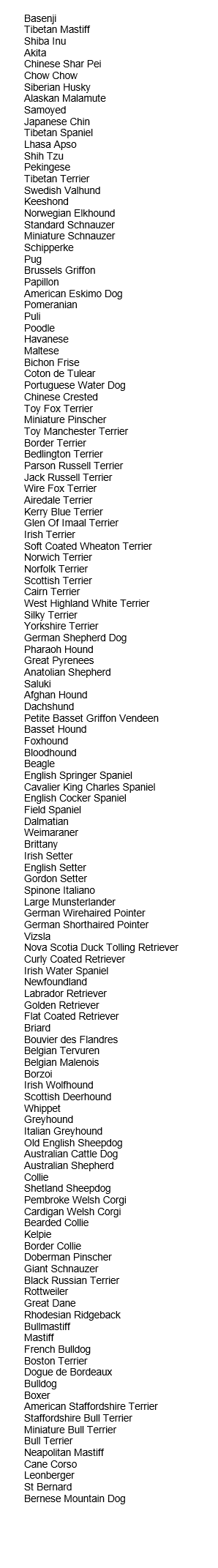

Supplement: Supplementary file 4 — Supplementary Figure 4. [file 41598_2023_50458_MOESM4_ESM.docx]
