# Supplementary material for: Longevity of companion dog breeds: those at risk from early death
Source: Sci Rep. 2024 Feb 1;14:531. doi: 10.1038/s41598-023-50458-w (PMC10834484; doi:10.1038/s41598-023-50458-w)
Supplement: Supplementary file 5 — Supplementary Tables 6-11. [file 41598_2023_50458_MOESM5_ESM.docx]

***Table S6:*** *Samples sizes regarding alive* $\left( \boldsymbol{N}_{\boldsymbol{A}} \right)$*, deceased* $\left( \boldsymbol{N}_{\boldsymbol{D}} \right)$ *and total* $\left( \boldsymbol{N} \right)$ *individuals, per* ***Breed*** *included in survival analysis. Breed list includes Purebred i.e., 1 breed (n = 155) and Crossbred i.e., ≥* *2 breeds (n = 1).*

| ***Breed*** | ***Sample Sizes*** | | |
| --- | --- | --- | --- |
|  | $\boldsymbol{N}_{\boldsymbol{A}}$ | $\boldsymbol{N}_{\boldsymbol{D}}$ | $\boldsymbol{N}$ |
| ***Crossbreed*** | 38764 | 72445 | 111209 |
| ***Affenpinscher*** | 133 | 204 | 337 |
| ***Afghan Hound*** | 136 | 238 | 374 |
| ***Airedale Terrier*** | 681 | 556 | 1237 |
| ***Akita*** | 1458 | 2358 | 3816 |
| ***Alaskan Malamute*** | 1105 | 842 | 1947 |
| ***American Cocker Spaniel*** | 339 | 293 | 632 |
| ***American Eskimo Dog*** | 16 | 22 | 38 |
| ***American Staffordshire Terrier*** | 40 | 94 | 134 |
| ***Anatolian Shepherd*** | 34 | 40 | 74 |
| ***Australian Cattle Dog*** | 75 | 50 | 125 |
| ***Australian Shepherd*** | 183 | 62 | 245 |
| ***Basenji*** | 47 | 30 | 77 |
| ***Basset Hound*** | 1114 | 883 | 1997 |
| ***Beagle*** | 3112 | 1552 | 4664 |
| ***Bearded Collie*** | 574 | 570 | 1144 |
| ***Bedlington Terrier*** | 720 | 549 | 1269 |
| ***Belgian Malenois*** | 284 | 337 | 621 |
| ***Belgian Tervuren*** | 154 | 55 | 209 |
| ***Bernese Mountain Dog*** | 622 | 449 | 1071 |
| ***Bichon Frise*** | 2987 | 2900 | 5887 |
| ***Black Russian Terrier*** | 95 | 71 | 166 |
| ***Bloodhound*** | 73 | 71 | 144 |
| ***Bolognese*** | 162 | 32 | 194 |
| ***Border Collie*** | 4974 | 7969 | 12943 |
| ***Border Terrier*** | 6825 | 2427 | 9252 |
| ***Borzoi*** | 94 | 43 | 137 |
| ***Boston Terrier*** | 1486 | 443 | 1929 |
| ***Bouvier des Flandres*** | 72 | 79 | 151 |
| ***Boxer*** | 5262 | 5925 | 11187 |
| ***Bracco Italiano*** | 95 | 22 | 117 |
| ***Briard*** | 119 | 76 | 195 |
| ***Brittany*** | 231 | 734 | 965 |
| ***Brussels Griffon*** | 219 | 102 | 321 |
| ***Bull Terrier*** | 2338 | 2151 | 4489 |
| ***Bulldog*** | 6400 | 4743 | 11143 |
| ***Bullmastiff*** | 967 | 1169 | 2136 |
| ***Cairn Terrier*** | 1421 | 1139 | 2560 |
| ***Canaan Dog*** | 18 | 28 | 46 |
| ***Cane Corso*** | 91 | 101 | 192 |
| ***Cardigan Welsh Corgi*** | 142 | 69 | 211 |
| ***Caucasian Shepherd Dog*** | 31 | 22 | 53 |
| ***Cavalier King Charles Spaniel*** | 6987 | 6879 | 13866 |
| ***Chesapeake Bay Retriever*** | 87 | 42 | 129 |
| ***Chihuahua*** | 6989 | 3848 | 10837 |
| ***Chinese Crested*** | 494 | 394 | 888 |
| ***Chinese Shar Pei*** | 2233 | 2028 | 4261 |
| ***Chow Chow*** | 604 | 395 | 999 |
| ***Clumber Spaniel*** | 253 | 85 | 338 |
| ***Collie*** | 1101 | 1104 | 2205 |
| ***Coton de Tulear*** | 284 | 44 | 328 |
| ***Curly Coated Retriever*** | 87 | 66 | 153 |
| ***Dachshund*** | 1399 | 926 | 2325 |
| ***Dalmatian*** | 1750 | 1443 | 3193 |
| ***Dandie Dinmont Terrier*** | 129 | 66 | 195 |
| ***Doberman Pinscher*** | 1908 | 1623 | 3531 |
| ***Dogue de Bordeaux*** | 2311 | 1204 | 3515 |
| ***English Cocker Spaniel*** | 19007 | 7584 | 26591 |
| ***English Setter*** | 335 | 238 | 573 |
| ***English Springer Spaniel*** | 11514 | 6221 | 17735 |
| ***Field Spaniel*** | 105 | 136 | 241 |
| ***Finnish Lapphund*** | 97 | 23 | 120 |
| ***Flat Coated Retriever*** | 1362 | 784 | 2146 |
| ***Foxhound*** | 53 | 31 | 84 |
| ***French Bulldog*** | 9358 | 2495 | 11853 |
| ***German Shepherd Dog*** | 9074 | 10773 | 19847 |
| ***German Shorthaired Pointer*** | 2336 | 947 | 3283 |
| ***German Spitz Mittel*** | 135 | 96 | 231 |
| ***German Wirehaired Pointer*** | 490 | 138 | 628 |
| ***Giant Schnauzer*** | 250 | 126 | 376 |
| ***Glen Of Imaal Terrier*** | 64 | 40 | 104 |
| ***Golden Retriever*** | 7252 | 4252 | 11504 |
| ***Gordon Setter*** | 272 | 138 | 410 |
| ***Great Dane*** | 1117 | 864 | 1981 |
| ***Great Pyrenees*** | 144 | 128 | 272 |
| ***Greyhound*** | 1007 | 3025 | 4032 |
| ***Havanese*** | 333 | 56 | 389 |
| ***Irish Setter*** | 952 | 547 | 1499 |
| ***Irish Terrier*** | 375 | 146 | 521 |
| ***Irish Water Spaniel*** | 115 | 68 | 183 |
| ***Irish Wolfhound*** | 259 | 210 | 469 |
| ***Italian Greyhound*** | 323 | 70 | 393 |
| ***Jack Russell Terrier*** | 5680 | 13497 | 19177 |
| ***Japanese Chin*** | 223 | 142 | 365 |
| ***Japanese Spitz*** | 244 | 117 | 361 |
| ***Keeshond*** | 113 | 55 | 168 |
| ***Kelpie*** | 58 | 49 | 107 |
| ***Kerry Blue Terrier*** | 222 | 168 | 390 |
| ***Labrador Retriever*** | 27027 | 16155 | 43182 |
| ***Lakeland Terrier*** | 466 | 601 | 1067 |
| ***Lancashire Heeler*** | 168 | 110 | 278 |
| ***Large Munsterlander*** | 111 | 26 | 137 |
| ***Leonberger*** | 378 | 321 | 699 |
| ***Lhasa Apso*** | 4329 | 2723 | 7052 |
| ***Lowchen*** | 110 | 50 | 160 |
| ***Maltese*** | 1398 | 666 | 2064 |
| ***Mastiff*** | 430 | 1419 | 1849 |
| ***Miniature Bull Terrier*** | 200 | 94 | 294 |
| ***Miniature Dachshund*** | 5616 | 1655 | 7271 |
| ***Miniature Pinscher*** | 422 | 167 | 589 |
| ***Miniature Schnauzer*** | 5469 | 1977 | 7446 |
| ***Neapolitan Mastiff*** | 150 | 210 | 360 |
| ***Newfoundland*** | 923 | 633 | 1556 |
| ***Norfolk Terrier*** | 673 | 297 | 970 |
| ***Norwegian Elkhound*** | 65 | 64 | 129 |
| ***Norwich Terrier*** | 160 | 45 | 205 |
| ***Nova Scotia Duck Tolling Retriever*** | 201 | 47 | 248 |
| ***Old English Sheepdog*** | 560 | 604 | 1164 |
| ***Papillon*** | 687 | 427 | 1114 |
| ***Parson Russell Terrier*** | 1323 | 1176 | 2499 |
| ***Pekingese*** | 401 | 339 | 740 |
| ***Pembroke Welsh Corgi*** | 434 | 221 | 655 |
| ***Petite Basset Griffon Vendeen*** | 328 | 148 | 476 |
| ***Pharaoh Hound*** | 22 | 23 | 45 |
| ***Polish Lowland Sheepdog*** | 59 | 24 | 83 |
| ***Pomeranian*** | 1737 | 1170 | 2907 |
| ***Poodle*** | 3969 | 2399 | 6368 |
| ***Portuguese Water Dog*** | 153 | 36 | 189 |
| ***Presa Canario*** | 65 | 98 | 163 |
| ***Pug*** | 7257 | 2782 | 10039 |
| ***Puli*** | 86 | 39 | 125 |
| ***Rhodesian Ridgeback*** | 1214 | 883 | 2097 |
| ***Rottweiler*** | 2875 | 3442 | 6317 |
| ***Saluki*** | 281 | 169 | 450 |
| ***Samoyed*** | 379 | 336 | 715 |
| ***Schipperke*** | 47 | 28 | 75 |
| ***Scottish Deerhound*** | 226 | 191 | 417 |
| ***Scottish Terrier*** | 867 | 869 | 1736 |
| ***Sealyham Terrier*** | 98 | 33 | 131 |
| ***Shetland Sheepdog*** | 1091 | 793 | 1884 |
| ***Shiba Inu*** | 305 | 91 | 396 |
| ***Shih Tzu*** | 6191 | 5894 | 12085 |
| ***Siberian Husky*** | 2609 | 1947 | 4556 |
| ***Silky Terrier*** | 54 | 45 | 99 |
| ***Skye Terrier*** | 43 | 39 | 82 |
| ***Soft Coated Wheaten Terrier*** | 440 | 173 | 613 |
| ***Spanish Water Dog*** | 201 | 37 | 238 |
| ***Spinone Italiano*** | 472 | 215 | 687 |
| ***St Bernard*** | 603 | 509 | 1112 |
| ***Staffordshire Bull Terrier*** | 11035 | 25332 | 36367 |
| ***Standard Schnauzer*** | 364 | 435 | 799 |
| ***Sussex Spaniel*** | 57 | 34 | 91 |
| ***Swedish Valhund*** | 34 | 22 | 56 |
| ***Tibetan Mastiff*** | 72 | 22 | 94 |
| ***Tibetan Spaniel*** | 193 | 137 | 330 |
| ***Tibetan Terrier*** | 1407 | 583 | 1990 |
| ***Toy Fox Terrier*** | 117 | 52 | 169 |
| ***Toy Manchester Terrier*** | 194 | 138 | 332 |
| ***Vizsla*** | 2562 | 503 | 3065 |
| ***Weimaraner*** | 1883 | 1229 | 3112 |
| ***Welsh Springer Spaniel*** | 375 | 162 | 537 |
| ***Welsh Terrier*** | 430 | 146 | 576 |
| ***West Highland White Terrier*** | 5365 | 6730 | 12095 |
| ***Whippet*** | 3486 | 1483 | 4969 |
| ***Wire Fox Terrier*** | 891 | 521 | 1412 |
| ***Yorkshire Terrier*** | 4984 | 9509 | 14493 |
| ***TOTAL*** | 300000 | 284734 | 584734 |

***Table S7:*** *Samples sizes regarding alive* $\left( \boldsymbol{N}_{\boldsymbol{A}} \right)$*, deceased* $\left( \boldsymbol{N}_{\boldsymbol{D}} \right)$ *and total* $\left( \boldsymbol{N} \right)$ *individuals, per* ***Parental Lineage*** *included in survival analysis.* ***Parental Lineage*** *includes Purebred i.e., 1 breed and Crossbred i.e.,* **≥** *2 breeds.*

| ***Parental Lineage*** | ***Sample Sizes*** | | |
| --- | --- | --- | --- |
|  | $\boldsymbol{N}_{\boldsymbol{A}}$ | $\boldsymbol{N}_{\boldsymbol{D}}$ | $\boldsymbol{N}$ |
| ***Purebred i.e., 1 breed*** | 261134 | 212289 | 473423 |
| ***Crossbred i.e.,* ≥ *2 breeds*** | 38866 | *72445* | *111311* |
| ***TOTAL*** | 300000 | 284734 | 584734 |

***Table S8:*** *Samples sizes regarding alive* $\left( \boldsymbol{N}_{\boldsymbol{A}} \right)$*, deceased* $\left( \boldsymbol{N}_{\boldsymbol{D}} \right)$ *and total* $\left( \boldsymbol{N} \right)$ *individuals, per* ***Sex*** *included in survival analysis.*

| ***Sex*** | ***Sample Sizes*** | | |
| --- | --- | --- | --- |
|  | $\boldsymbol{N}_{\boldsymbol{A}}$ | $\boldsymbol{N}_{\boldsymbol{D}}$ | $\boldsymbol{N}$ |
| ***Female*** | 147444 | 136910 | 284354 |
| ***Male*** | 152556 | 147824 | 300380 |
| ***TOTAL*** | 300000 | 284734 | 584734 |

***Table S9:*** *Samples sizes regarding alive* $\left( \boldsymbol{N}_{\boldsymbol{A}} \right)$*, deceased* $\left( \boldsymbol{N}_{\boldsymbol{D}} \right)$ *and total* $\left( \boldsymbol{N} \right)$ *individuals, per* ***Age Group*** *included in survival analysis.*

| ***Age Group*** | ***Sample Sizes*** | | |
| --- | --- | --- | --- |
|  | $\boldsymbol{N}_{\boldsymbol{A}}$ | $\boldsymbol{N}_{\boldsymbol{D}}$ | $\boldsymbol{N}$ |
| ***Puppies*** | *2623* | *6675* | *9298* |
| ***Juveniles*** | *8456* | *4770* | *13226* |
| ***Young Adult*** | 26843 | 9426 | 36269 |
| ***Mature Adult*** | 121233 | 49289 | 170522 |
| ***Senior Adult*** | 99234 | 99729 | 198963 |
| ***Geriatric*** | 41611 | 114845 | 156456 |
| ***TOTAL*** | 300000 | 284734 | 584734 |

***Table S10:*** *Samples sizes regarding alive* $\left( \boldsymbol{N}_{\boldsymbol{A}} \right)$*, deceased* $\left( \boldsymbol{N}_{\boldsymbol{D}} \right)$ *and total* $\left( \boldsymbol{N} \right)$ *individuals, per* ***Body Size*** *included in survival analysis.*

| ***Body Size*** | ***Sample Sizes*** | | |
| --- | --- | --- | --- |
|  | $\boldsymbol{N}_{\boldsymbol{A}}$ | $\boldsymbol{N}_{\boldsymbol{D}}$ | $\boldsymbol{N}$ |
| ***Large*** | 61202 | 61976 | 123178 |
| ***Medium*** | 38121 | 35787 | 73908 |
| ***Small*** | 110677 | 112063 | 222740 |
| ***TOTAL*** | 210000 | 209826 | 419826 |

***Table S11:*** *Samples sizes regarding alive* $\left( \boldsymbol{N}_{\boldsymbol{A}} \right)$*, deceased* $\left( \boldsymbol{N}_{\boldsymbol{D}} \right)$ *and total* $\left( \boldsymbol{N} \right)$ *individuals, per* ***Cephalic Index*** *included in survival analysis.*

| ***Cephalic Index*** | ***Sample Sizes*** | | |
| --- | --- | --- | --- |
|  | $\boldsymbol{N}_{\boldsymbol{A}}$ | $\boldsymbol{N}_{\boldsymbol{D}}$ | $\boldsymbol{N}$ |
| ***Brachycephalic*** | 47753 | 40644 | 88397 |
| ***Mesocephalic*** | 129313 | 138357 | 267760 |
| ***Dolicocephalic*** | 32934 | 33224 | 66158 |
| ***Total*** | 210000 | 212225 | 422225 |
