# Supplementary material for: Longevity of companion dog breeds: those at risk from early death
Source: Sci Rep. 2024 Feb 1;14:531. doi: 10.1038/s41598-023-50458-w (PMC10834484; doi:10.1038/s41598-023-50458-w)
Supplement: Supplementary file 6 — Supplementary Table 1. [file 41598_2023_50458_MOESM6_ESM.docx]

***Table S1:*** *Probability of* ***Purebred*** *(i.e., Parental Lineage = 1 breed) and* ***Crossbred*** *(i.e., Parental Lineage ≥ 2 breeds) survival, per* ***Decimal Year****. Includes the following statistics per decimal year:* $\boldsymbol{N}_{\boldsymbol{A}}$ *i.e., total number of individuals still alive;* $\boldsymbol{N}_{\boldsymbol{D}}$ *i.e., total number of deaths;* ***Survival Probability*** *(%);* ***Standard Error*** *(SE);* ***Lower 95% Confidence Interval*** *and* ***Upper 95% Confidence Interval****. Highlighted rows identify that 95% of purebred and crossbred individuals are deceased by the age of 18.3 and 18.2 years, respectively.*

|  | ***Purebred i.e., 1 breed*** | | | | | | ***Crossbred i.e.,* ≥ *2 breeds*** | | | | | |
| --- | --- | --- | --- | --- | --- | --- | --- | --- | --- | --- | --- | --- |
| ***Decimal Year*** | $\boldsymbol{N}_{\boldsymbol{A}}$ | $\boldsymbol{N}_{\boldsymbol{D}}$ | ***Survival Probability*** | ***SE*** | ***Lower 95% CI*** | ***Upper 95% CI*** | $\boldsymbol{N}_{\boldsymbol{A}}$ | $\boldsymbol{N}_{\boldsymbol{D}}$ | ***Survival Probability*** | ***SE*** | ***Lower 95% CI*** | ***Upper 95% CI*** |
| 0 | 473681 | 367 | 0.999 | 0.000 | 0.999 | 0.999 | 111053 | 258 | 0.998 | 0.000 | 0.997 | 0.998 |
| 0.1 | 472928 | 541 | 0.998 | 0.000 | 0.998 | 0.998 | 110678 | 252 | 0.995 | 0.000 | 0.995 | 0.996 |
| 0.2 | 472373 | 1809 | 0.994 | 0.000 | 0.994 | 0.994 | 110420 | 665 | 0.989 | 0.000 | 0.989 | 0.990 |
| 0.3 | 470368 | 1358 | 0.991 | 0.000 | 0.991 | 0.992 | 109649 | 482 | 0.985 | 0.000 | 0.984 | 0.986 |
| 0.4 | 468564 | 696 | 0.990 | 0.000 | 0.990 | 0.990 | 108877 | 247 | 0.983 | 0.000 | 0.982 | 0.984 |
| 0.5 | 467127 | 717 | 0.988 | 0.000 | 0.988 | 0.989 | 108322 | 266 | 0.980 | 0.000 | 0.980 | 0.981 |
| 0.6 | 465571 | 624 | 0.987 | 0.000 | 0.987 | 0.987 | 107753 | 239 | 0.978 | 0.000 | 0.977 | 0.979 |
| 0.7 | 464028 | 668 | 0.986 | 0.000 | 0.985 | 0.986 | 107179 | 226 | 0.976 | 0.000 | 0.975 | 0.977 |
| 0.8 | 462414 | 885 | 0.984 | 0.000 | 0.983 | 0.984 | 106648 | 338 | 0.973 | 0.000 | 0.972 | 0.974 |
| 0.9 | 459441 | 601 | 0.982 | 0.000 | 0.982 | 0.983 | 105858 | 206 | 0.971 | 0.001 | 0.970 | 0.972 |
| 1 | 456858 | 818 | 0.981 | 0.000 | 0.980 | 0.981 | 105275 | 353 | 0.968 | 0.001 | 0.967 | 0.969 |
| 1.1 | 453769 | 661 | 0.979 | 0.000 | 0.979 | 0.980 | 104471 | 258 | 0.966 | 0.001 | 0.964 | 0.967 |
| 1.2 | 450927 | 670 | 0.978 | 0.000 | 0.977 | 0.978 | 103865 | 274 | 0.963 | 0.001 | 0.962 | 0.964 |
| 1.3 | 448038 | 956 | 0.976 | 0.000 | 0.975 | 0.976 | 103203 | 387 | 0.959 | 0.001 | 0.958 | 0.961 |
| 1.4 | 444361 | 594 | 0.974 | 0.000 | 0.974 | 0.975 | 102284 | 240 | 0.957 | 0.001 | 0.956 | 0.958 |
| 1.5 | 441413 | 633 | 0.973 | 0.000 | 0.973 | 0.974 | 101659 | 268 | 0.955 | 0.001 | 0.953 | 0.956 |
| 1.6 | 438861 | 571 | 0.972 | 0.000 | 0.971 | 0.972 | 101015 | 221 | 0.953 | 0.001 | 0.951 | 0.954 |
| 1.7 | 436176 | 534 | 0.971 | 0.000 | 0.970 | 0.971 | 100405 | 201 | 0.951 | 0.001 | 0.949 | 0.952 |
| 1.8 | 433644 | 778 | 0.969 | 0.000 | 0.968 | 0.969 | 99769 | 314 | 0.948 | 0.001 | 0.946 | 0.949 |
| 1.9 | 429997 | 513 | 0.968 | 0.000 | 0.967 | 0.968 | 98951 | 182 | 0.946 | 0.001 | 0.945 | 0.947 |
| 2 | 427258 | 832 | 0.966 | 0.000 | 0.965 | 0.966 | 98442 | 442 | 0.942 | 0.001 | 0.940 | 0.943 |
| 2.1 | 423859 | 574 | 0.965 | 0.000 | 0.964 | 0.965 | 97603 | 239 | 0.939 | 0.001 | 0.938 | 0.941 |
| 2.2 | 421230 | 631 | 0.963 | 0.000 | 0.963 | 0.964 | 97024 | 241 | 0.937 | 0.001 | 0.936 | 0.938 |
| 2.3 | 418178 | 843 | 0.961 | 0.000 | 0.961 | 0.962 | 96470 | 332 | 0.934 | 0.001 | 0.932 | 0.935 |
| 2.4 | 414347 | 564 | 0.960 | 0.000 | 0.959 | 0.960 | 95682 | 223 | 0.932 | 0.001 | 0.930 | 0.933 |
| 2.5 | 411477 | 588 | 0.958 | 0.000 | 0.958 | 0.959 | 95103 | 263 | 0.929 | 0.001 | 0.927 | 0.931 |
| 2.6 | 408759 | 530 | 0.957 | 0.000 | 0.957 | 0.958 | 94542 | 212 | 0.927 | 0.001 | 0.925 | 0.928 |
| 2.7 | 406299 | 571 | 0.956 | 0.000 | 0.955 | 0.956 | 93975 | 194 | 0.925 | 0.001 | 0.923 | 0.927 |
| 2.8 | 403773 | 795 | 0.954 | 0.000 | 0.953 | 0.955 | 93455 | 288 | 0.922 | 0.001 | 0.921 | 0.924 |
| 2.9 | 400145 | 511 | 0.953 | 0.000 | 0.952 | 0.953 | 92731 | 170 | 0.920 | 0.001 | 0.919 | 0.922 |
| 3 | 397397 | 964 | 0.950 | 0.000 | 0.950 | 0.951 | 92242 | 422 | 0.916 | 0.001 | 0.915 | 0.918 |
| 3.1 | 393938 | 618 | 0.949 | 0.000 | 0.948 | 0.950 | 91392 | 256 | 0.914 | 0.001 | 0.912 | 0.915 |
| 3.2 | 391189 | 609 | 0.947 | 0.000 | 0.947 | 0.948 | 90817 | 221 | 0.911 | 0.001 | 0.910 | 0.913 |
| 3.3 | 388206 | 853 | 0.945 | 0.000 | 0.945 | 0.946 | 90260 | 314 | 0.908 | 0.001 | 0.907 | 0.910 |
| 3.4 | 384458 | 567 | 0.944 | 0.000 | 0.943 | 0.945 | 89489 | 191 | 0.906 | 0.001 | 0.905 | 0.908 |
| 3.5 | 381665 | 561 | 0.943 | 0.000 | 0.942 | 0.943 | 88924 | 223 | 0.904 | 0.001 | 0.902 | 0.906 |
| 3.6 | 379067 | 538 | 0.941 | 0.000 | 0.941 | 0.942 | 88331 | 186 | 0.902 | 0.001 | 0.900 | 0.904 |
| 3.7 | 376651 | 574 | 0.940 | 0.000 | 0.939 | 0.941 | 87793 | 176 | 0.900 | 0.001 | 0.899 | 0.902 |
| 3.8 | 374179 | 800 | 0.938 | 0.000 | 0.937 | 0.939 | 87332 | 328 | 0.897 | 0.001 | 0.895 | 0.899 |
| 3.9 | 370838 | 539 | 0.936 | 0.000 | 0.936 | 0.937 | 86576 | 185 | 0.895 | 0.001 | 0.893 | 0.897 |
| 4 | 368242 | 1006 | 0.934 | 0.000 | 0.933 | 0.935 | 86083 | 425 | 0.891 | 0.001 | 0.889 | 0.893 |
| 4.1 | 364810 | 610 | 0.932 | 0.000 | 0.932 | 0.933 | 85249 | 206 | 0.889 | 0.001 | 0.887 | 0.890 |
| 4.2 | 362285 | 556 | 0.931 | 0.000 | 0.930 | 0.932 | 84753 | 234 | 0.886 | 0.001 | 0.884 | 0.888 |
| 4.3 | 359639 | 876 | 0.929 | 0.000 | 0.928 | 0.929 | 84232 | 300 | 0.883 | 0.001 | 0.881 | 0.885 |
| 4.4 | 356374 | 578 | 0.927 | 0.000 | 0.926 | 0.928 | 83558 | 221 | 0.881 | 0.001 | 0.879 | 0.883 |
| 4.5 | 353895 | 650 | 0.925 | 0.000 | 0.925 | 0.926 | 83005 | 215 | 0.878 | 0.001 | 0.876 | 0.880 |
| 4.6 | 351494 | 570 | 0.924 | 0.000 | 0.923 | 0.925 | 82444 | 185 | 0.876 | 0.001 | 0.874 | 0.878 |
| 4.7 | 349234 | 557 | 0.922 | 0.000 | 0.922 | 0.923 | 81976 | 169 | 0.875 | 0.001 | 0.872 | 0.877 |
| 4.8 | 347016 | 840 | 0.920 | 0.000 | 0.919 | 0.921 | 81497 | 299 | 0.871 | 0.001 | 0.869 | 0.873 |
| 4.9 | 343869 | 609 | 0.919 | 0.000 | 0.918 | 0.919 | 80845 | 189 | 0.869 | 0.001 | 0.867 | 0.871 |
| 5 | 341343 | 1192 | 0.915 | 0.000 | 0.915 | 0.916 | 80323 | 451 | 0.864 | 0.001 | 0.862 | 0.866 |
| 5.1 | 337753 | 652 | 0.914 | 0.000 | 0.913 | 0.914 | 79470 | 258 | 0.862 | 0.001 | 0.859 | 0.864 |
| 5.2 | 335288 | 628 | 0.912 | 0.000 | 0.911 | 0.913 | 78969 | 222 | 0.859 | 0.001 | 0.857 | 0.861 |
| 5.3 | 332691 | 920 | 0.909 | 0.000 | 0.909 | 0.910 | 78474 | 341 | 0.855 | 0.001 | 0.853 | 0.858 |
| 5.4 | 329356 | 664 | 0.908 | 0.000 | 0.907 | 0.908 | 77800 | 196 | 0.853 | 0.001 | 0.851 | 0.855 |
| 5.5 | 326787 | 735 | 0.906 | 0.000 | 0.905 | 0.906 | 77318 | 211 | 0.851 | 0.001 | 0.849 | 0.853 |
| 5.6 | 324288 | 649 | 0.904 | 0.000 | 0.903 | 0.905 | 76802 | 225 | 0.848 | 0.001 | 0.846 | 0.851 |
| 5.7 | 321920 | 626 | 0.902 | 0.000 | 0.901 | 0.903 | 76290 | 211 | 0.846 | 0.001 | 0.844 | 0.848 |
| 5.8 | 319758 | 940 | 0.899 | 0.000 | 0.898 | 0.900 | 75822 | 300 | 0.843 | 0.001 | 0.840 | 0.845 |
| 5.9 | 316617 | 677 | 0.897 | 0.000 | 0.896 | 0.898 | 75186 | 204 | 0.840 | 0.001 | 0.838 | 0.843 |
| 6 | 314115 | 1136 | 0.894 | 0.000 | 0.893 | 0.895 | 74698 | 460 | 0.835 | 0.001 | 0.833 | 0.838 |
| 6.1 | 310711 | 794 | 0.892 | 0.000 | 0.891 | 0.893 | 73887 | 252 | 0.832 | 0.001 | 0.830 | 0.835 |
| 6.2 | 308091 | 763 | 0.890 | 0.000 | 0.889 | 0.891 | 73371 | 219 | 0.830 | 0.001 | 0.828 | 0.832 |
| 6.3 | 305340 | 1119 | 0.886 | 0.001 | 0.885 | 0.887 | 72932 | 342 | 0.826 | 0.001 | 0.824 | 0.828 |
| 6.4 | 301857 | 732 | 0.884 | 0.001 | 0.883 | 0.885 | 72291 | 245 | 0.823 | 0.001 | 0.821 | 0.826 |
| 6.5 | 299334 | 779 | 0.882 | 0.001 | 0.881 | 0.883 | 71774 | 235 | 0.821 | 0.001 | 0.818 | 0.823 |
| 6.6 | 296832 | 758 | 0.880 | 0.001 | 0.879 | 0.881 | 71293 | 201 | 0.818 | 0.001 | 0.816 | 0.821 |
| 6.7 | 294426 | 742 | 0.877 | 0.001 | 0.876 | 0.878 | 70845 | 246 | 0.815 | 0.001 | 0.813 | 0.818 |
| 6.8 | 292109 | 1174 | 0.874 | 0.001 | 0.873 | 0.875 | 70342 | 334 | 0.812 | 0.001 | 0.809 | 0.814 |
| 6.9 | 288770 | 785 | 0.872 | 0.001 | 0.871 | 0.873 | 69666 | 208 | 0.809 | 0.001 | 0.807 | 0.812 |
| 7 | 286113 | 1414 | 0.867 | 0.001 | 0.866 | 0.868 | 69160 | 577 | 0.802 | 0.001 | 0.800 | 0.805 |
| 7.1 | 282389 | 888 | 0.865 | 0.001 | 0.863 | 0.866 | 68262 | 283 | 0.799 | 0.001 | 0.797 | 0.802 |
| 7.2 | 279692 | 934 | 0.862 | 0.001 | 0.861 | 0.863 | 67745 | 229 | 0.796 | 0.001 | 0.794 | 0.799 |
| 7.3 | 276729 | 1350 | 0.857 | 0.001 | 0.856 | 0.859 | 67279 | 400 | 0.792 | 0.001 | 0.789 | 0.794 |
| 7.4 | 273068 | 870 | 0.855 | 0.001 | 0.854 | 0.856 | 66599 | 251 | 0.789 | 0.001 | 0.786 | 0.791 |
| 7.5 | 270184 | 1072 | 0.851 | 0.001 | 0.850 | 0.852 | 66137 | 280 | 0.785 | 0.001 | 0.783 | 0.788 |
| 7.6 | 267533 | 886 | 0.848 | 0.001 | 0.847 | 0.850 | 65637 | 247 | 0.782 | 0.001 | 0.780 | 0.785 |
| 7.7 | 265127 | 971 | 0.845 | 0.001 | 0.844 | 0.847 | 65173 | 229 | 0.780 | 0.001 | 0.777 | 0.782 |
| 7.8 | 262498 | 1431 | 0.841 | 0.001 | 0.840 | 0.842 | 64729 | 414 | 0.775 | 0.001 | 0.772 | 0.777 |
| 7.9 | 258904 | 981 | 0.838 | 0.001 | 0.836 | 0.839 | 64016 | 239 | 0.772 | 0.001 | 0.769 | 0.774 |
| 8 | 255944 | 1883 | 0.831 | 0.001 | 0.830 | 0.833 | 63533 | 696 | 0.763 | 0.001 | 0.761 | 0.766 |
| 8.1 | 251818 | 1141 | 0.828 | 0.001 | 0.826 | 0.829 | 62485 | 326 | 0.759 | 0.001 | 0.757 | 0.762 |
| 8.2 | 248995 | 1120 | 0.824 | 0.001 | 0.823 | 0.825 | 61961 | 284 | 0.756 | 0.001 | 0.753 | 0.759 |
| 8.3 | 245844 | 1609 | 0.819 | 0.001 | 0.817 | 0.820 | 61472 | 457 | 0.750 | 0.001 | 0.747 | 0.753 |
| 8.4 | 241960 | 1100 | 0.815 | 0.001 | 0.814 | 0.816 | 60741 | 302 | 0.746 | 0.001 | 0.744 | 0.749 |
| 8.5 | 238844 | 1250 | 0.811 | 0.001 | 0.809 | 0.812 | 60262 | 374 | 0.742 | 0.001 | 0.739 | 0.745 |
| 8.6 | 235919 | 1169 | 0.807 | 0.001 | 0.805 | 0.808 | 59655 | 319 | 0.738 | 0.001 | 0.735 | 0.741 |
| 8.7 | 233163 | 1072 | 0.803 | 0.001 | 0.802 | 0.804 | 59106 | 288 | 0.734 | 0.001 | 0.731 | 0.737 |
| 8.8 | 230430 | 1781 | 0.797 | 0.001 | 0.795 | 0.798 | 58604 | 530 | 0.728 | 0.001 | 0.725 | 0.731 |
| 8.9 | 226534 | 1164 | 0.793 | 0.001 | 0.791 | 0.794 | 57802 | 324 | 0.724 | 0.001 | 0.721 | 0.726 |
| 9 | 223645 | 2067 | 0.785 | 0.001 | 0.784 | 0.787 | 57273 | 813 | 0.713 | 0.002 | 0.710 | 0.716 |
| 9.1 | 219523 | 1328 | 0.780 | 0.001 | 0.779 | 0.782 | 56172 | 421 | 0.708 | 0.002 | 0.705 | 0.711 |
| 9.2 | 216453 | 1305 | 0.776 | 0.001 | 0.774 | 0.777 | 55555 | 354 | 0.703 | 0.002 | 0.700 | 0.706 |
| 9.3 | 213245 | 1977 | 0.769 | 0.001 | 0.767 | 0.770 | 55023 | 515 | 0.697 | 0.002 | 0.694 | 0.700 |
| 9.4 | 209067 | 1279 | 0.764 | 0.001 | 0.762 | 0.765 | 54262 | 337 | 0.692 | 0.002 | 0.689 | 0.696 |
| 9.5 | 205998 | 1455 | 0.758 | 0.001 | 0.757 | 0.760 | 53736 | 418 | 0.687 | 0.002 | 0.684 | 0.690 |
| 9.6 | 202953 | 1293 | 0.754 | 0.001 | 0.752 | 0.755 | 53130 | 373 | 0.682 | 0.002 | 0.679 | 0.685 |
| 9.7 | 200245 | 1323 | 0.749 | 0.001 | 0.747 | 0.750 | 52557 | 348 | 0.678 | 0.002 | 0.675 | 0.681 |
| 9.8 | 197424 | 1947 | 0.741 | 0.001 | 0.740 | 0.743 | 52035 | 565 | 0.670 | 0.002 | 0.667 | 0.674 |
| 9.9 | 193550 | 1302 | 0.736 | 0.001 | 0.735 | 0.738 | 51243 | 369 | 0.666 | 0.002 | 0.662 | 0.669 |
| 10 | 190678 | 2538 | 0.726 | 0.001 | 0.725 | 0.728 | 50663 | 980 | 0.653 | 0.002 | 0.650 | 0.656 |
| 10.1 | 186065 | 1554 | 0.720 | 0.001 | 0.719 | 0.722 | 49403 | 503 | 0.646 | 0.002 | 0.643 | 0.649 |
| 10.2 | 182809 | 1464 | 0.715 | 0.001 | 0.713 | 0.716 | 48757 | 422 | 0.640 | 0.002 | 0.637 | 0.644 |
| 10.3 | 179580 | 2292 | 0.706 | 0.001 | 0.704 | 0.707 | 48184 | 685 | 0.631 | 0.002 | 0.628 | 0.635 |
| 10.4 | 175383 | 1445 | 0.700 | 0.001 | 0.698 | 0.701 | 47303 | 441 | 0.625 | 0.002 | 0.622 | 0.629 |
| 10.5 | 172448 | 1768 | 0.693 | 0.001 | 0.691 | 0.694 | 46717 | 470 | 0.619 | 0.002 | 0.616 | 0.622 |
| 10.6 | 169219 | 1489 | 0.686 | 0.001 | 0.685 | 0.688 | 46056 | 441 | 0.613 | 0.002 | 0.610 | 0.617 |
| 10.7 | 166408 | 1506 | 0.680 | 0.001 | 0.679 | 0.682 | 45486 | 404 | 0.608 | 0.002 | 0.605 | 0.611 |
| 10.8 | 163550 | 2350 | 0.670 | 0.001 | 0.669 | 0.672 | 44922 | 660 | 0.599 | 0.002 | 0.596 | 0.602 |
| 10.9 | 159519 | 1545 | 0.664 | 0.001 | 0.662 | 0.666 | 44084 | 437 | 0.593 | 0.002 | 0.590 | 0.596 |
| 11 | 156330 | 2832 | 0.652 | 0.001 | 0.650 | 0.654 | 43512 | 959 | 0.580 | 0.002 | 0.577 | 0.583 |
| 11.1 | 151606 | 1717 | 0.645 | 0.001 | 0.643 | 0.646 | 42329 | 518 | 0.573 | 0.002 | 0.569 | 0.576 |
| 11.2 | 148303 | 1674 | 0.637 | 0.001 | 0.636 | 0.639 | 41675 | 453 | 0.567 | 0.002 | 0.563 | 0.570 |
| 11.3 | 144910 | 2476 | 0.626 | 0.001 | 0.625 | 0.628 | 41083 | 714 | 0.557 | 0.002 | 0.553 | 0.560 |
| 11.4 | 140474 | 1658 | 0.619 | 0.001 | 0.617 | 0.621 | 40213 | 449 | 0.550 | 0.002 | 0.547 | 0.554 |
| 11.5 | 137097 | 1882 | 0.610 | 0.001 | 0.609 | 0.612 | 39631 | 505 | 0.543 | 0.002 | 0.540 | 0.547 |
| 11.6 | 133726 | 1726 | 0.603 | 0.001 | 0.601 | 0.604 | 38948 | 523 | 0.536 | 0.002 | 0.533 | 0.540 |
| 11.7 | 130546 | 1585 | 0.595 | 0.001 | 0.593 | 0.597 | 38266 | 453 | 0.530 | 0.002 | 0.526 | 0.533 |
| 11.8 | 127665 | 2491 | 0.584 | 0.001 | 0.582 | 0.586 | 37706 | 691 | 0.520 | 0.002 | 0.517 | 0.524 |
| 11.9 | 123431 | 1663 | 0.576 | 0.001 | 0.574 | 0.578 | 36848 | 442 | 0.514 | 0.002 | 0.510 | 0.517 |
| 12 | 120144 | 3163 | 0.561 | 0.001 | 0.559 | 0.563 | 36276 | 1090 | 0.498 | 0.002 | 0.495 | 0.502 |
| 12.1 | 115293 | 1915 | 0.551 | 0.001 | 0.549 | 0.553 | 34997 | 566 | 0.490 | 0.002 | 0.487 | 0.494 |
| 12.2 | 111857 | 1799 | 0.542 | 0.001 | 0.541 | 0.544 | 34286 | 502 | 0.483 | 0.002 | 0.480 | 0.487 |
| 12.3 | 108280 | 2690 | 0.529 | 0.001 | 0.527 | 0.531 | 33656 | 777 | 0.472 | 0.002 | 0.469 | 0.476 |
| 12.4 | 103828 | 1757 | 0.520 | 0.001 | 0.518 | 0.522 | 32738 | 507 | 0.465 | 0.002 | 0.461 | 0.468 |
| 12.5 | 100360 | 1983 | 0.510 | 0.001 | 0.508 | 0.512 | 32126 | 550 | 0.457 | 0.002 | 0.453 | 0.460 |
| 12.6 | 96949 | 1836 | 0.500 | 0.001 | 0.498 | 0.502 | 31427 | 511 | 0.449 | 0.002 | 0.446 | 0.453 |
| 12.7 | 93765 | 1729 | 0.491 | 0.001 | 0.489 | 0.493 | 30784 | 489 | 0.442 | 0.002 | 0.439 | 0.446 |
| 12.8 | 90673 | 2625 | 0.477 | 0.001 | 0.475 | 0.479 | 30193 | 747 | 0.431 | 0.002 | 0.428 | 0.435 |
| 12.9 | 86490 | 1725 | 0.467 | 0.001 | 0.465 | 0.469 | 29307 | 503 | 0.424 | 0.002 | 0.420 | 0.427 |
| 13 | 83249 | 3388 | 0.448 | 0.001 | 0.446 | 0.450 | 28696 | 1134 | 0.407 | 0.002 | 0.404 | 0.411 |
| 13.1 | 78165 | 1902 | 0.437 | 0.001 | 0.435 | 0.439 | 27376 | 610 | 0.398 | 0.002 | 0.395 | 0.402 |
| 13.2 | 74809 | 1849 | 0.426 | 0.001 | 0.424 | 0.429 | 26648 | 516 | 0.390 | 0.002 | 0.387 | 0.394 |
| 13.3 | 71319 | 2596 | 0.411 | 0.001 | 0.409 | 0.413 | 26029 | 729 | 0.379 | 0.002 | 0.376 | 0.383 |
| 13.4 | 67126 | 1765 | 0.400 | 0.001 | 0.398 | 0.402 | 25170 | 500 | 0.372 | 0.002 | 0.368 | 0.375 |
| 13.5 | 63791 | 1917 | 0.388 | 0.001 | 0.386 | 0.390 | 24577 | 568 | 0.363 | 0.002 | 0.360 | 0.367 |
| 13.6 | 60539 | 1743 | 0.377 | 0.001 | 0.375 | 0.379 | 23871 | 553 | 0.355 | 0.002 | 0.351 | 0.358 |
| 13.7 | 57545 | 1564 | 0.367 | 0.001 | 0.365 | 0.369 | 23227 | 440 | 0.348 | 0.002 | 0.345 | 0.352 |
| 13.8 | 54859 | 2277 | 0.351 | 0.001 | 0.349 | 0.354 | 22698 | 770 | 0.336 | 0.002 | 0.333 | 0.340 |
| 13.9 | 51613 | 1481 | 0.341 | 0.001 | 0.339 | 0.343 | 21798 | 433 | 0.330 | 0.002 | 0.326 | 0.333 |
| 14 | 49600 | 3025 | 0.321 | 0.001 | 0.318 | 0.323 | 21280 | 1184 | 0.311 | 0.002 | 0.308 | 0.315 |
| 14.1 | 46126 | 1513 | 0.310 | 0.001 | 0.308 | 0.312 | 19967 | 538 | 0.303 | 0.002 | 0.300 | 0.306 |
| 14.2 | 44314 | 1409 | 0.300 | 0.001 | 0.298 | 0.302 | 19331 | 487 | 0.295 | 0.002 | 0.292 | 0.299 |
| 14.3 | 42622 | 2008 | 0.286 | 0.001 | 0.284 | 0.288 | 18775 | 754 | 0.283 | 0.002 | 0.280 | 0.287 |
| 14.4 | 40351 | 1252 | 0.277 | 0.001 | 0.275 | 0.279 | 17938 | 474 | 0.276 | 0.002 | 0.273 | 0.279 |
| 14.5 | 38898 | 1361 | 0.267 | 0.001 | 0.265 | 0.270 | 17388 | 512 | 0.268 | 0.002 | 0.265 | 0.271 |
| 14.6 | 37304 | 1246 | 0.259 | 0.001 | 0.256 | 0.261 | 16786 | 450 | 0.261 | 0.002 | 0.257 | 0.264 |
| 14.7 | 35850 | 1136 | 0.250 | 0.001 | 0.248 | 0.252 | 16280 | 433 | 0.254 | 0.002 | 0.250 | 0.257 |
| 14.8 | 34498 | 1690 | 0.238 | 0.001 | 0.236 | 0.240 | 15776 | 657 | 0.243 | 0.002 | 0.240 | 0.246 |
| 14.9 | 32552 | 1061 | 0.230 | 0.001 | 0.228 | 0.232 | 15016 | 423 | 0.236 | 0.002 | 0.233 | 0.239 |
| 15 | 31296 | 2336 | 0.213 | 0.001 | 0.211 | 0.215 | 14511 | 1030 | 0.220 | 0.002 | 0.216 | 0.223 |
| 15.1 | 28727 | 1094 | 0.205 | 0.001 | 0.203 | 0.207 | 13377 | 458 | 0.212 | 0.002 | 0.209 | 0.215 |
| 15.2 | 27440 | 995 | 0.198 | 0.001 | 0.196 | 0.200 | 12866 | 385 | 0.206 | 0.002 | 0.203 | 0.209 |
| 15.3 | 26311 | 1422 | 0.187 | 0.001 | 0.185 | 0.189 | 12440 | 558 | 0.196 | 0.002 | 0.193 | 0.199 |
| 15.4 | 24678 | 874 | 0.180 | 0.001 | 0.178 | 0.182 | 11816 | 367 | 0.190 | 0.002 | 0.187 | 0.193 |
| 15.5 | 23652 | 913 | 0.173 | 0.001 | 0.171 | 0.175 | 11390 | 438 | 0.183 | 0.002 | 0.180 | 0.186 |
| 15.6 | 22603 | 835 | 0.167 | 0.001 | 0.165 | 0.169 | 10892 | 348 | 0.177 | 0.001 | 0.174 | 0.180 |
| 15.7 | 21623 | 779 | 0.161 | 0.001 | 0.159 | 0.163 | 10493 | 351 | 0.171 | 0.001 | 0.168 | 0.174 |
| 15.8 | 20713 | 1074 | 0.153 | 0.001 | 0.151 | 0.154 | 10105 | 480 | 0.163 | 0.001 | 0.160 | 0.166 |
| 15.9 | 19472 | 721 | 0.147 | 0.001 | 0.145 | 0.149 | 9579 | 339 | 0.157 | 0.001 | 0.155 | 0.160 |
| 16 | 18624 | 1525 | 0.135 | 0.001 | 0.133 | 0.137 | 9180 | 851 | 0.143 | 0.001 | 0.140 | 0.145 |
| 16.1 | 16944 | 726 | 0.129 | 0.001 | 0.127 | 0.131 | 8283 | 328 | 0.137 | 0.001 | 0.134 | 0.140 |
| 16.2 | 16096 | 616 | 0.124 | 0.001 | 0.122 | 0.126 | 7900 | 289 | 0.132 | 0.001 | 0.129 | 0.135 |
| 16.3 | 15376 | 779 | 0.118 | 0.001 | 0.116 | 0.120 | 7579 | 408 | 0.125 | 0.001 | 0.122 | 0.128 |
| 16.4 | 14476 | 503 | 0.114 | 0.001 | 0.112 | 0.115 | 7132 | 239 | 0.121 | 0.001 | 0.118 | 0.123 |
| 16.5 | 13887 | 546 | 0.109 | 0.001 | 0.108 | 0.111 | 6860 | 293 | 0.116 | 0.001 | 0.113 | 0.118 |
| 16.6 | 13233 | 505 | 0.105 | 0.001 | 0.104 | 0.107 | 6524 | 236 | 0.111 | 0.001 | 0.109 | 0.114 |
| 16.7 | 12626 | 450 | 0.101 | 0.001 | 0.100 | 0.103 | 6264 | 205 | 0.108 | 0.001 | 0.105 | 0.110 |
| 16.8 | 12093 | 573 | 0.097 | 0.001 | 0.095 | 0.098 | 6029 | 281 | 0.103 | 0.001 | 0.100 | 0.105 |
| 16.9 | 11399 | 347 | 0.094 | 0.001 | 0.092 | 0.095 | 5710 | 208 | 0.099 | 0.001 | 0.097 | 0.101 |
| 17 | 10972 | 864 | 0.086 | 0.001 | 0.085 | 0.088 | 5470 | 555 | 0.089 | 0.001 | 0.087 | 0.091 |
| 17.1 | 9963 | 380 | 0.083 | 0.001 | 0.082 | 0.084 | 4852 | 186 | 0.086 | 0.001 | 0.083 | 0.088 |
| 17.2 | 9502 | 345 | 0.080 | 0.001 | 0.079 | 0.081 | 4628 | 177 | 0.082 | 0.001 | 0.080 | 0.085 |
| 17.3 | 9084 | 441 | 0.076 | 0.001 | 0.075 | 0.078 | 4431 | 231 | 0.078 | 0.001 | 0.076 | 0.080 |
| 17.4 | 8551 | 273 | 0.074 | 0.001 | 0.072 | 0.075 | 4160 | 155 | 0.075 | 0.001 | 0.073 | 0.077 |
| 17.5 | 8201 | 291 | 0.071 | 0.001 | 0.070 | 0.072 | 3978 | 166 | 0.072 | 0.001 | 0.070 | 0.074 |
| 17.6 | 7839 | 278 | 0.069 | 0.001 | 0.067 | 0.070 | 3779 | 123 | 0.070 | 0.001 | 0.068 | 0.072 |
| 17.7 | 7493 | 248 | 0.066 | 0.001 | 0.065 | 0.068 | 3626 | 126 | 0.067 | 0.001 | 0.065 | 0.069 |
| 17.8 | 7188 | 307 | 0.063 | 0.001 | 0.062 | 0.065 | 3485 | 192 | 0.063 | 0.001 | 0.062 | 0.066 |
| 17.9 | 6807 | 217 | 0.061 | 0.001 | 0.060 | 0.063 | 3266 | 122 | 0.061 | 0.001 | 0.059 | 0.063 |
| 18 | 6536 | 490 | 0.057 | 0.001 | 0.056 | 0.058 | 3118 | 348 | 0.054 | 0.001 | 0.052 | 0.056 |
| 18.1 | 5963 | 235 | 0.055 | 0.001 | 0.053 | 0.056 | 2740 | 128 | 0.052 | 0.001 | 0.050 | 0.054 |
| 18.2 | 5675 | 201 | 0.053 | 0.001 | 0.051 | 0.054 | 2589 | 86 | 0.050 | 0.001 | 0.048 | 0.052 |
| 18.3 | 5433 | 245 | 0.050 | 0.001 | 0.049 | 0.051 | 2484 | 131 | 0.047 | 0.001 | 0.046 | 0.049 |
| 18.4 | 5138 | 191 | 0.048 | 0.001 | 0.047 | 0.050 | 2336 | 81 | 0.046 | 0.001 | 0.044 | 0.048 |
| 18.5 | 4908 | 181 | 0.047 | 0.001 | 0.045 | 0.048 | 2236 | 80 | 0.044 | 0.001 | 0.042 | 0.046 |
| 18.6 | 4693 | 165 | 0.045 | 0.001 | 0.044 | 0.046 | 2126 | 85 | 0.042 | 0.001 | 0.041 | 0.044 |
| 18.7 | 4490 | 140 | 0.044 | 0.001 | 0.042 | 0.045 | 2023 | 70 | 0.041 | 0.001 | 0.039 | 0.043 |
| 18.8 | 4314 | 164 | 0.042 | 0.001 | 0.041 | 0.043 | 1935 | 89 | 0.039 | 0.001 | 0.037 | 0.041 |
| 18.9 | 4094 | 175 | 0.040 | 0.001 | 0.039 | 0.041 | 1821 | 64 | 0.038 | 0.001 | 0.036 | 0.039 |
| 19 | 3876 | 201 | 0.038 | 0.001 | 0.037 | 0.039 | 1740 | 129 | 0.035 | 0.001 | 0.033 | 0.036 |
| 19.1 | 3615 | 142 | 0.037 | 0.001 | 0.035 | 0.038 | 1577 | 58 | 0.034 | 0.001 | 0.032 | 0.035 |
| 19.2 | 3439 | 97 | 0.036 | 0.001 | 0.034 | 0.037 | 1504 | 49 | 0.032 | 0.001 | 0.031 | 0.034 |
| 19.3 | 3303 | 113 | 0.034 | 0.001 | 0.033 | 0.035 | 1444 | 86 | 0.031 | 0.001 | 0.029 | 0.032 |
| 19.4 | 3153 | 105 | 0.033 | 0.001 | 0.032 | 0.034 | 1341 | 50 | 0.029 | 0.001 | 0.028 | 0.031 |
| 19.5 | 3021 | 91 | 0.032 | 0.001 | 0.031 | 0.033 | 1282 | 39 | 0.029 | 0.001 | 0.027 | 0.030 |
| 19.6 | 2902 | 104 | 0.031 | 0.001 | 0.030 | 0.032 | 1224 | 48 | 0.027 | 0.001 | 0.026 | 0.029 |
| 19.7 | 2769 | 67 | 0.030 | 0.001 | 0.029 | 0.031 | 1161 | 39 | 0.026 | 0.001 | 0.025 | 0.028 |
| 19.8 | 2676 | 91 | 0.029 | 0.001 | 0.028 | 0.030 | 1108 | 44 | 0.025 | 0.001 | 0.024 | 0.027 |
| 19.9 | 2548 | 74 | 0.028 | 0.001 | 0.027 | 0.029 | 1047 | 42 | 0.024 | 0.001 | 0.023 | 0.026 |
| 20 | 2445 | 120 | 0.027 | 0.000 | 0.026 | 0.028 | 991 | 82 | 0.022 | 0.001 | 0.021 | 0.024 |
| 20.1 | 2284 | 74 | 0.026 | 0.000 | 0.025 | 0.027 | 882 | 24 | 0.022 | 0.001 | 0.021 | 0.023 |
| 20.2 | 2189 | 71 | 0.025 | 0.000 | 0.024 | 0.026 | 839 | 33 | 0.021 | 0.001 | 0.020 | 0.022 |
| 20.3 | 2101 | 71 | 0.024 | 0.000 | 0.023 | 0.025 | 795 | 31 | 0.020 | 0.001 | 0.019 | 0.021 |
| 20.4 | 2011 | 64 | 0.024 | 0.000 | 0.023 | 0.025 | 750 | 31 | 0.019 | 0.001 | 0.018 | 0.021 |
| 20.5 | 1929 | 53 | 0.023 | 0.000 | 0.022 | 0.024 | 708 | 12 | 0.019 | 0.001 | 0.018 | 0.020 |
| 20.6 | 1866 | 84 | 0.022 | 0.000 | 0.021 | 0.023 | 688 | 25 | 0.018 | 0.001 | 0.017 | 0.020 |
| 20.7 | 1761 | 50 | 0.021 | 0.000 | 0.020 | 0.022 | 654 | 23 | 0.018 | 0.001 | 0.016 | 0.019 |
| 20.8 | 1699 | 65 | 0.021 | 0.000 | 0.020 | 0.021 | 621 | 15 | 0.017 | 0.001 | 0.016 | 0.018 |
| 20.9 | 1618 | 49 | 0.020 | 0.000 | 0.019 | 0.021 | 589 | 21 | 0.017 | 0.001 | 0.015 | 0.018 |
| 21 | 1558 | 76 | 0.019 | 0.000 | 0.018 | 0.020 | 560 | 22 | 0.016 | 0.001 | 0.015 | 0.017 |
| 21.1 | 1464 | 70 | 0.018 | 0.000 | 0.017 | 0.019 | 519 | 17 | 0.015 | 0.001 | 0.014 | 0.017 |
| 21.2 | 1375 | 45 | 0.017 | 0.000 | 0.017 | 0.018 | 492 | 19 | 0.015 | 0.001 | 0.014 | 0.016 |
| 21.3 | 1317 | 51 | 0.017 | 0.000 | 0.016 | 0.018 | 466 | 16 | 0.014 | 0.001 | 0.013 | 0.015 |
| 21.4 | 1250 | 47 | 0.016 | 0.000 | 0.015 | 0.017 | 441 | 12 | 0.014 | 0.001 | 0.013 | 0.015 |
| 21.5 | 1191 | 34 | 0.016 | 0.000 | 0.015 | 0.016 | 420 | 14 | 0.013 | 0.001 | 0.012 | 0.015 |
| 21.6 | 1148 | 54 | 0.015 | 0.000 | 0.014 | 0.016 | 398 | 18 | 0.013 | 0.001 | 0.012 | 0.014 |
| 21.7 | 1083 | 47 | 0.014 | 0.000 | 0.014 | 0.015 | 372 | 9 | 0.013 | 0.001 | 0.011 | 0.014 |
| 21.8 | 1031 | 36 | 0.014 | 0.000 | 0.013 | 0.015 | 356 | 13 | 0.012 | 0.001 | 0.011 | 0.013 |
| 21.9 | 987 | 53 | 0.013 | 0.000 | 0.012 | 0.014 | 338 | 11 | 0.012 | 0.001 | 0.011 | 0.013 |
| 22 | 926 | 50 | 0.012 | 0.000 | 0.012 | 0.013 | 323 | 24 | 0.011 | 0.001 | 0.010 | 0.012 |
| 22.1 | 858 | 76 | 0.011 | 0.000 | 0.011 | 0.012 | 281 | 18 | 0.010 | 0.001 | 0.009 | 0.011 |
| 22.2 | 771 | 28 | 0.011 | 0.000 | 0.010 | 0.012 | 258 | 4 | 0.010 | 0.001 | 0.009 | 0.011 |
| 22.3 | 734 | 31 | 0.010 | 0.000 | 0.010 | 0.011 | 251 | 7 | 0.010 | 0.001 | 0.009 | 0.011 |
| 22.4 | 694 | 37 | 0.010 | 0.000 | 0.009 | 0.011 | 238 | 13 | 0.009 | 0.001 | 0.008 | 0.010 |
| 22.5 | 649 | 35 | 0.009 | 0.000 | 0.009 | 0.010 | 222 | 8 | 0.009 | 0.001 | 0.008 | 0.010 |
| 22.6 | 604 | 42 | 0.009 | 0.000 | 0.008 | 0.009 | 210 | 6 | 0.009 | 0.000 | 0.008 | 0.010 |
| 22.7 | 555 | 38 | 0.008 | 0.000 | 0.007 | 0.009 | 202 | 6 | 0.008 | 0.000 | 0.007 | 0.009 |
| 22.8 | 511 | 31 | 0.008 | 0.000 | 0.007 | 0.008 | 190 | 7 | 0.008 | 0.000 | 0.007 | 0.009 |
| 22.9 | 470 | 37 | 0.007 | 0.000 | 0.006 | 0.008 | 181 | 8 | 0.008 | 0.000 | 0.007 | 0.009 |
| 23 | 427 | 38 | 0.006 | 0.000 | 0.006 | 0.007 | 172 | 17 | 0.007 | 0.000 | 0.006 | 0.008 |
| 23.1 | 378 | 53 | 0.005 | 0.000 | 0.005 | 0.006 | 146 | 10 | 0.006 | 0.000 | 0.006 | 0.007 |
| 23.2 | 314 | 24 | 0.005 | 0.000 | 0.005 | 0.006 | 131 | 6 | 0.006 | 0.000 | 0.005 | 0.007 |
| 23.3 | 288 | 32 | 0.004 | 0.000 | 0.004 | 0.005 | 120 | 4 | 0.006 | 0.000 | 0.005 | 0.007 |
| 23.4 | 251 | 24 | 0.004 | 0.000 | 0.004 | 0.005 | 111 | 6 | 0.006 | 0.000 | 0.005 | 0.007 |
| 23.5 | 216 | 24 | 0.004 | 0.000 | 0.003 | 0.004 | 102 | 14 | 0.005 | 0.000 | 0.004 | 0.006 |
| 23.6 | 186 | 33 | 0.003 | 0.000 | 0.003 | 0.003 | 85 | 9 | 0.004 | 0.000 | 0.004 | 0.005 |
| 23.7 | 150 | 32 | 0.002 | 0.000 | 0.002 | 0.003 | 70 | 9 | 0.004 | 0.000 | 0.003 | 0.005 |
| 23.8 | 113 | 25 | 0.002 | 0.000 | 0.001 | 0.002 | 59 | 7 | 0.003 | 0.000 | 0.003 | 0.004 |
| 23.9 | 81 | 27 | 0.001 | 0.000 | 0.001 | 0.002 | 47 | 8 | 0.003 | 0.000 | 0.002 | 0.004 |
| 24 | 49 | 34 | 0.000 | 0.000 | 0.000 | 0.001 | 35 | 29 | 0.000 | 0.000 | 0.000 | 0.001 |
