# Supplementary material for: Longevity of companion dog breeds: those at risk from early death
Source: Sci Rep. 2024 Feb 1;14:531. doi: 10.1038/s41598-023-50458-w (PMC10834484; doi:10.1038/s41598-023-50458-w)
Supplement: Supplementary file 7 — Supplementary Table 2. [file 41598_2023_50458_MOESM7_ESM.docx]

***Table S2:*** *Probability of* ***Female*** *and* ***Male*** *survival (include purebred and crossbred individuals), per* ***Decimal Year****. Includes the following statistics per decimal year:* $\boldsymbol{N}_{\boldsymbol{A}}$ *i.e., total number of individuals still alive;* $\boldsymbol{N}_{\boldsymbol{D}}$ *i.e., total number of deaths;* ***Survival Probability*** *(%);* ***Standard Error*** *(SE);* ***Lower 95% Confidence Interval*** *and* ***Upper 95% Confidence Interval****. Highlighted rows identify that 95% of both sexes are deceased by the age of 18.3 years.*

|  | ***Female*** | | | | | | ***Male*** | | | | | |
| --- | --- | --- | --- | --- | --- | --- | --- | --- | --- | --- | --- | --- |
| ***Decimal Year*** | $\boldsymbol{N}_{\boldsymbol{A}}$ | $\boldsymbol{N}_{\boldsymbol{D}}$ | ***Survival Probability*** | ***SE*** | ***Lower 95% CI*** | ***Upper 95% CI*** | $\boldsymbol{N}_{\boldsymbol{A}}$ | $\boldsymbol{N}_{\boldsymbol{D}}$ | ***Survival Probability*** | ***SE*** | ***Lower 95% CI*** | ***Upper 95% CI*** |
| 0 | 283984 | 282 | 0.999 | 0.000 | 0.999 | 0.999 | 300750 | 343 | 0.999 | 0.000 | 0.999 | 0.999 |
| 0.1 | 283472 | 393 | 0.998 | 0.000 | 0.997 | 0.998 | 300144 | 400 | 0.998 | 0.000 | 0.997 | 0.998 |
| 0.2 | 283072 | 1177 | 0.993 | 0.000 | 0.993 | 0.994 | 299727 | 1297 | 0.993 | 0.000 | 0.993 | 0.994 |
| 0.3 | 281757 | 923 | 0.990 | 0.000 | 0.990 | 0.991 | 298281 | 917 | 0.990 | 0.000 | 0.990 | 0.991 |
| 0.4 | 280498 | 452 | 0.989 | 0.000 | 0.988 | 0.989 | 296963 | 491 | 0.989 | 0.000 | 0.988 | 0.989 |
| 0.5 | 279524 | 434 | 0.987 | 0.000 | 0.987 | 0.988 | 295933 | 549 | 0.987 | 0.000 | 0.986 | 0.987 |
| 0.6 | 278614 | 412 | 0.986 | 0.000 | 0.985 | 0.986 | 294726 | 451 | 0.985 | 0.000 | 0.985 | 0.986 |
| 0.7 | 277614 | 402 | 0.984 | 0.000 | 0.984 | 0.985 | 293573 | 492 | 0.984 | 0.000 | 0.983 | 0.984 |
| 0.8 | 276626 | 544 | 0.982 | 0.000 | 0.982 | 0.983 | 292418 | 679 | 0.981 | 0.000 | 0.981 | 0.982 |
| 0.9 | 274828 | 338 | 0.981 | 0.000 | 0.981 | 0.982 | 290435 | 469 | 0.980 | 0.000 | 0.979 | 0.980 |
| 1 | 273360 | 510 | 0.979 | 0.000 | 0.979 | 0.980 | 288764 | 661 | 0.977 | 0.000 | 0.977 | 0.978 |
| 1.1 | 271564 | 398 | 0.978 | 0.000 | 0.977 | 0.978 | 286633 | 521 | 0.976 | 0.000 | 0.975 | 0.976 |
| 1.2 | 269934 | 421 | 0.976 | 0.000 | 0.976 | 0.977 | 284730 | 523 | 0.974 | 0.000 | 0.973 | 0.974 |
| 1.3 | 268247 | 573 | 0.974 | 0.000 | 0.974 | 0.975 | 282930 | 770 | 0.971 | 0.000 | 0.971 | 0.972 |
| 1.4 | 266148 | 337 | 0.973 | 0.000 | 0.972 | 0.974 | 280480 | 497 | 0.969 | 0.000 | 0.969 | 0.970 |
| 1.5 | 264508 | 393 | 0.972 | 0.000 | 0.971 | 0.972 | 278571 | 508 | 0.968 | 0.000 | 0.967 | 0.968 |
| 1.6 | 262994 | 334 | 0.970 | 0.000 | 0.970 | 0.971 | 276796 | 458 | 0.966 | 0.000 | 0.965 | 0.967 |
| 1.7 | 261508 | 318 | 0.969 | 0.000 | 0.968 | 0.970 | 274948 | 417 | 0.965 | 0.000 | 0.964 | 0.965 |
| 1.8 | 260022 | 476 | 0.967 | 0.000 | 0.967 | 0.968 | 273295 | 616 | 0.962 | 0.000 | 0.962 | 0.963 |
| 1.9 | 257997 | 297 | 0.966 | 0.000 | 0.966 | 0.967 | 270927 | 398 | 0.961 | 0.000 | 0.960 | 0.962 |
| 2 | 256420 | 590 | 0.964 | 0.000 | 0.963 | 0.965 | 269197 | 684 | 0.959 | 0.000 | 0.958 | 0.959 |
| 2.1 | 254413 | 346 | 0.963 | 0.000 | 0.962 | 0.963 | 266987 | 467 | 0.957 | 0.000 | 0.956 | 0.958 |
| 2.2 | 252839 | 384 | 0.961 | 0.000 | 0.960 | 0.962 | 265249 | 488 | 0.955 | 0.000 | 0.954 | 0.956 |
| 2.3 | 251087 | 545 | 0.959 | 0.000 | 0.958 | 0.960 | 263276 | 630 | 0.953 | 0.000 | 0.952 | 0.954 |
| 2.4 | 248944 | 327 | 0.958 | 0.000 | 0.957 | 0.959 | 260881 | 460 | 0.951 | 0.000 | 0.950 | 0.952 |
| 2.5 | 247429 | 364 | 0.956 | 0.000 | 0.956 | 0.957 | 259128 | 487 | 0.949 | 0.000 | 0.949 | 0.950 |
| 2.6 | 245859 | 343 | 0.955 | 0.000 | 0.954 | 0.956 | 257413 | 399 | 0.948 | 0.000 | 0.947 | 0.949 |
| 2.7 | 244407 | 337 | 0.954 | 0.000 | 0.953 | 0.955 | 255826 | 428 | 0.946 | 0.000 | 0.946 | 0.947 |
| 2.8 | 242942 | 499 | 0.952 | 0.000 | 0.951 | 0.953 | 254233 | 584 | 0.944 | 0.000 | 0.943 | 0.945 |
| 2.9 | 240892 | 316 | 0.951 | 0.000 | 0.950 | 0.951 | 251945 | 365 | 0.943 | 0.000 | 0.942 | 0.944 |
| 3 | 239324 | 639 | 0.948 | 0.000 | 0.947 | 0.949 | 250251 | 747 | 0.940 | 0.000 | 0.939 | 0.941 |
| 3.1 | 237276 | 408 | 0.946 | 0.000 | 0.946 | 0.947 | 248022 | 466 | 0.938 | 0.000 | 0.937 | 0.939 |
| 3.2 | 235628 | 381 | 0.945 | 0.000 | 0.944 | 0.946 | 246278 | 449 | 0.937 | 0.000 | 0.936 | 0.937 |
| 3.3 | 233897 | 516 | 0.943 | 0.000 | 0.942 | 0.944 | 244453 | 651 | 0.934 | 0.000 | 0.933 | 0.935 |
| 3.4 | 231832 | 330 | 0.941 | 0.000 | 0.941 | 0.942 | 242087 | 428 | 0.932 | 0.000 | 0.931 | 0.933 |
| 3.5 | 230197 | 360 | 0.940 | 0.000 | 0.939 | 0.941 | 240294 | 424 | 0.931 | 0.000 | 0.930 | 0.932 |
| 3.6 | 228654 | 309 | 0.939 | 0.000 | 0.938 | 0.940 | 238620 | 415 | 0.929 | 0.000 | 0.928 | 0.930 |
| 3.7 | 227270 | 336 | 0.937 | 0.000 | 0.936 | 0.938 | 237078 | 414 | 0.927 | 0.000 | 0.927 | 0.928 |
| 3.8 | 225840 | 499 | 0.935 | 0.000 | 0.934 | 0.936 | 235567 | 629 | 0.925 | 0.000 | 0.924 | 0.926 |
| 3.9 | 223874 | 333 | 0.934 | 0.000 | 0.933 | 0.935 | 233416 | 391 | 0.923 | 0.001 | 0.922 | 0.924 |
| 4 | 222414 | 689 | 0.931 | 0.000 | 0.930 | 0.932 | 231811 | 742 | 0.921 | 0.001 | 0.920 | 0.922 |
| 4.1 | 220373 | 356 | 0.929 | 0.001 | 0.928 | 0.930 | 229581 | 460 | 0.919 | 0.001 | 0.918 | 0.920 |
| 4.2 | 218926 | 354 | 0.928 | 0.001 | 0.927 | 0.929 | 227970 | 436 | 0.917 | 0.001 | 0.916 | 0.918 |
| 4.3 | 217411 | 525 | 0.926 | 0.001 | 0.925 | 0.927 | 226328 | 651 | 0.914 | 0.001 | 0.913 | 0.915 |
| 4.4 | 215430 | 358 | 0.924 | 0.001 | 0.923 | 0.925 | 224239 | 441 | 0.912 | 0.001 | 0.911 | 0.914 |
| 4.5 | 213950 | 395 | 0.922 | 0.001 | 0.921 | 0.924 | 222682 | 470 | 0.911 | 0.001 | 0.909 | 0.912 |
| 4.6 | 212543 | 355 | 0.921 | 0.001 | 0.920 | 0.922 | 221084 | 400 | 0.909 | 0.001 | 0.908 | 0.910 |
| 4.7 | 211193 | 319 | 0.920 | 0.001 | 0.919 | 0.921 | 219614 | 407 | 0.907 | 0.001 | 0.906 | 0.908 |
| 4.8 | 209893 | 490 | 0.917 | 0.001 | 0.916 | 0.918 | 218251 | 649 | 0.905 | 0.001 | 0.903 | 0.906 |
| 4.9 | 208099 | 361 | 0.916 | 0.001 | 0.915 | 0.917 | 216259 | 437 | 0.903 | 0.001 | 0.902 | 0.904 |
| 5 | 206577 | 751 | 0.912 | 0.001 | 0.911 | 0.914 | 214712 | 892 | 0.899 | 0.001 | 0.898 | 0.900 |
| 5.1 | 204480 | 409 | 0.911 | 0.001 | 0.910 | 0.912 | 212380 | 501 | 0.897 | 0.001 | 0.896 | 0.898 |
| 5.2 | 203138 | 386 | 0.909 | 0.001 | 0.908 | 0.910 | 210844 | 464 | 0.895 | 0.001 | 0.894 | 0.896 |
| 5.3 | 201662 | 569 | 0.906 | 0.001 | 0.905 | 0.908 | 209194 | 692 | 0.892 | 0.001 | 0.891 | 0.893 |
| 5.4 | 199704 | 371 | 0.905 | 0.001 | 0.904 | 0.906 | 207176 | 489 | 0.890 | 0.001 | 0.889 | 0.891 |
| 5.5 | 198285 | 433 | 0.903 | 0.001 | 0.902 | 0.904 | 205646 | 513 | 0.888 | 0.001 | 0.886 | 0.889 |
| 5.6 | 196838 | 401 | 0.901 | 0.001 | 0.900 | 0.902 | 204040 | 473 | 0.886 | 0.001 | 0.884 | 0.887 |
| 5.7 | 195468 | 357 | 0.899 | 0.001 | 0.898 | 0.900 | 202570 | 480 | 0.883 | 0.001 | 0.882 | 0.885 |
| 5.8 | 194215 | 557 | 0.897 | 0.001 | 0.895 | 0.898 | 201130 | 683 | 0.880 | 0.001 | 0.879 | 0.882 |
| 5.9 | 192348 | 390 | 0.895 | 0.001 | 0.894 | 0.896 | 199166 | 491 | 0.878 | 0.001 | 0.877 | 0.879 |
| 6 | 190931 | 742 | 0.891 | 0.001 | 0.890 | 0.893 | 197648 | 854 | 0.874 | 0.001 | 0.873 | 0.876 |
| 6.1 | 188910 | 503 | 0.889 | 0.001 | 0.888 | 0.890 | 195460 | 543 | 0.872 | 0.001 | 0.871 | 0.873 |
| 6.2 | 187339 | 452 | 0.887 | 0.001 | 0.886 | 0.888 | 193859 | 530 | 0.870 | 0.001 | 0.868 | 0.871 |
| 6.3 | 185797 | 679 | 0.884 | 0.001 | 0.882 | 0.885 | 192199 | 782 | 0.866 | 0.001 | 0.865 | 0.867 |
| 6.4 | 183857 | 439 | 0.881 | 0.001 | 0.880 | 0.883 | 190084 | 538 | 0.864 | 0.001 | 0.862 | 0.865 |
| 6.5 | 182342 | 447 | 0.879 | 0.001 | 0.878 | 0.881 | 188444 | 567 | 0.861 | 0.001 | 0.860 | 0.862 |
| 6.6 | 180950 | 436 | 0.877 | 0.001 | 0.876 | 0.879 | 186945 | 523 | 0.859 | 0.001 | 0.857 | 0.860 |
| 6.7 | 179588 | 463 | 0.875 | 0.001 | 0.874 | 0.876 | 185430 | 525 | 0.856 | 0.001 | 0.855 | 0.858 |
| 6.8 | 178253 | 684 | 0.872 | 0.001 | 0.870 | 0.873 | 183945 | 824 | 0.852 | 0.001 | 0.851 | 0.854 |
| 6.9 | 176361 | 475 | 0.869 | 0.001 | 0.868 | 0.871 | 181847 | 518 | 0.850 | 0.001 | 0.849 | 0.851 |
| 7 | 174856 | 905 | 0.865 | 0.001 | 0.863 | 0.866 | 180198 | 1086 | 0.845 | 0.001 | 0.843 | 0.846 |
| 7.1 | 172759 | 560 | 0.862 | 0.001 | 0.861 | 0.863 | 177809 | 611 | 0.842 | 0.001 | 0.840 | 0.843 |
| 7.2 | 171156 | 504 | 0.859 | 0.001 | 0.858 | 0.861 | 176146 | 659 | 0.839 | 0.001 | 0.837 | 0.840 |
| 7.3 | 169589 | 827 | 0.855 | 0.001 | 0.854 | 0.857 | 174359 | 923 | 0.834 | 0.001 | 0.833 | 0.836 |
| 7.4 | 167511 | 529 | 0.853 | 0.001 | 0.851 | 0.854 | 172115 | 592 | 0.831 | 0.001 | 0.830 | 0.833 |
| 7.5 | 165878 | 631 | 0.849 | 0.001 | 0.848 | 0.851 | 170440 | 721 | 0.828 | 0.001 | 0.826 | 0.829 |
| 7.6 | 164327 | 486 | 0.847 | 0.001 | 0.845 | 0.848 | 168796 | 647 | 0.825 | 0.001 | 0.823 | 0.826 |
| 7.7 | 162956 | 555 | 0.844 | 0.001 | 0.842 | 0.845 | 167233 | 645 | 0.822 | 0.001 | 0.820 | 0.823 |
| 7.8 | 161513 | 862 | 0.839 | 0.001 | 0.838 | 0.841 | 165625 | 983 | 0.817 | 0.001 | 0.815 | 0.818 |
| 7.9 | 159413 | 581 | 0.836 | 0.001 | 0.835 | 0.838 | 163436 | 639 | 0.814 | 0.001 | 0.812 | 0.815 |
| 8 | 157749 | 1236 | 0.830 | 0.001 | 0.828 | 0.831 | 161663 | 1343 | 0.807 | 0.001 | 0.805 | 0.808 |
| 8.1 | 155245 | 656 | 0.826 | 0.001 | 0.825 | 0.828 | 158987 | 811 | 0.803 | 0.001 | 0.801 | 0.804 |
| 8.2 | 153655 | 638 | 0.823 | 0.001 | 0.821 | 0.824 | 157131 | 766 | 0.799 | 0.001 | 0.797 | 0.800 |
| 8.3 | 151938 | 997 | 0.817 | 0.001 | 0.816 | 0.819 | 155262 | 1069 | 0.793 | 0.001 | 0.792 | 0.795 |
| 8.4 | 149741 | 669 | 0.814 | 0.001 | 0.812 | 0.815 | 152871 | 733 | 0.789 | 0.001 | 0.788 | 0.791 |
| 8.5 | 147949 | 754 | 0.810 | 0.001 | 0.808 | 0.811 | 151020 | 870 | 0.785 | 0.001 | 0.783 | 0.787 |
| 8.6 | 146279 | 703 | 0.806 | 0.001 | 0.804 | 0.807 | 149215 | 785 | 0.781 | 0.001 | 0.779 | 0.782 |
| 8.7 | 144724 | 650 | 0.802 | 0.001 | 0.800 | 0.804 | 147475 | 710 | 0.777 | 0.001 | 0.775 | 0.779 |
| 8.8 | 143212 | 1094 | 0.796 | 0.001 | 0.794 | 0.798 | 145855 | 1217 | 0.771 | 0.001 | 0.769 | 0.772 |
| 8.9 | 140910 | 723 | 0.792 | 0.001 | 0.790 | 0.794 | 143449 | 765 | 0.766 | 0.001 | 0.765 | 0.768 |
| 9 | 139247 | 1424 | 0.784 | 0.001 | 0.782 | 0.786 | 141729 | 1456 | 0.759 | 0.001 | 0.757 | 0.760 |
| 9.1 | 136654 | 805 | 0.779 | 0.001 | 0.777 | 0.781 | 139031 | 944 | 0.753 | 0.001 | 0.752 | 0.755 |
| 9.2 | 134931 | 784 | 0.775 | 0.001 | 0.773 | 0.776 | 137155 | 875 | 0.749 | 0.001 | 0.747 | 0.750 |
| 9.3 | 133120 | 1183 | 0.768 | 0.001 | 0.766 | 0.770 | 135293 | 1309 | 0.741 | 0.001 | 0.739 | 0.743 |
| 9.4 | 130684 | 800 | 0.763 | 0.001 | 0.761 | 0.765 | 132786 | 816 | 0.737 | 0.001 | 0.735 | 0.739 |
| 9.5 | 128885 | 892 | 0.758 | 0.001 | 0.756 | 0.760 | 131021 | 981 | 0.731 | 0.001 | 0.729 | 0.733 |
| 9.6 | 127141 | 797 | 0.753 | 0.001 | 0.751 | 0.755 | 129152 | 869 | 0.726 | 0.001 | 0.724 | 0.728 |
| 9.7 | 125582 | 815 | 0.748 | 0.001 | 0.746 | 0.750 | 127496 | 856 | 0.721 | 0.001 | 0.720 | 0.723 |
| 9.8 | 123917 | 1234 | 0.741 | 0.001 | 0.739 | 0.743 | 125819 | 1278 | 0.714 | 0.001 | 0.712 | 0.716 |
| 9.9 | 121591 | 785 | 0.736 | 0.001 | 0.734 | 0.738 | 123466 | 886 | 0.709 | 0.001 | 0.707 | 0.711 |
| 10 | 119896 | 1734 | 0.725 | 0.001 | 0.723 | 0.727 | 121705 | 1784 | 0.699 | 0.001 | 0.697 | 0.701 |
| 10.1 | 117065 | 954 | 0.719 | 0.001 | 0.717 | 0.721 | 118762 | 1103 | 0.692 | 0.001 | 0.690 | 0.694 |
| 10.2 | 115224 | 880 | 0.714 | 0.001 | 0.712 | 0.716 | 116819 | 1006 | 0.686 | 0.001 | 0.684 | 0.688 |
| 10.3 | 113390 | 1395 | 0.705 | 0.001 | 0.703 | 0.707 | 114835 | 1582 | 0.677 | 0.001 | 0.675 | 0.679 |
| 10.4 | 110936 | 930 | 0.699 | 0.001 | 0.697 | 0.701 | 112173 | 956 | 0.671 | 0.001 | 0.669 | 0.673 |
| 10.5 | 109198 | 1078 | 0.692 | 0.001 | 0.690 | 0.694 | 110375 | 1160 | 0.664 | 0.001 | 0.662 | 0.666 |
| 10.6 | 107290 | 938 | 0.686 | 0.001 | 0.684 | 0.688 | 108422 | 992 | 0.658 | 0.001 | 0.656 | 0.660 |
| 10.7 | 105613 | 933 | 0.680 | 0.001 | 0.678 | 0.682 | 106734 | 977 | 0.652 | 0.001 | 0.650 | 0.654 |
| 10.8 | 103960 | 1510 | 0.670 | 0.001 | 0.668 | 0.672 | 105041 | 1500 | 0.642 | 0.001 | 0.640 | 0.645 |
| 10.9 | 101491 | 989 | 0.664 | 0.001 | 0.662 | 0.666 | 102626 | 993 | 0.636 | 0.001 | 0.634 | 0.638 |
| 11 | 99535 | 1923 | 0.651 | 0.001 | 0.649 | 0.653 | 100703 | 1868 | 0.624 | 0.001 | 0.622 | 0.627 |
| 11.1 | 96575 | 1127 | 0.643 | 0.001 | 0.641 | 0.646 | 97788 | 1108 | 0.617 | 0.001 | 0.615 | 0.620 |
| 11.2 | 94538 | 1018 | 0.636 | 0.001 | 0.634 | 0.639 | 95797 | 1109 | 0.610 | 0.001 | 0.608 | 0.612 |
| 11.3 | 92606 | 1543 | 0.626 | 0.001 | 0.624 | 0.628 | 93787 | 1647 | 0.600 | 0.001 | 0.597 | 0.602 |
| 11.4 | 89957 | 1019 | 0.619 | 0.001 | 0.616 | 0.621 | 91041 | 1088 | 0.592 | 0.001 | 0.590 | 0.595 |
| 11.5 | 87981 | 1187 | 0.610 | 0.001 | 0.608 | 0.613 | 88978 | 1200 | 0.584 | 0.001 | 0.582 | 0.587 |
| 11.6 | 85965 | 1098 | 0.603 | 0.001 | 0.600 | 0.605 | 86936 | 1151 | 0.577 | 0.001 | 0.574 | 0.579 |
| 11.7 | 84053 | 1017 | 0.595 | 0.001 | 0.593 | 0.598 | 85014 | 1021 | 0.570 | 0.001 | 0.567 | 0.572 |
| 11.8 | 82359 | 1543 | 0.584 | 0.001 | 0.582 | 0.586 | 83261 | 1639 | 0.558 | 0.001 | 0.556 | 0.561 |
| 11.9 | 79874 | 1027 | 0.577 | 0.001 | 0.574 | 0.579 | 80641 | 1078 | 0.551 | 0.001 | 0.549 | 0.553 |
| 12 | 77931 | 2120 | 0.561 | 0.001 | 0.559 | 0.563 | 78641 | 2133 | 0.536 | 0.001 | 0.534 | 0.538 |
| 12.1 | 74914 | 1212 | 0.552 | 0.001 | 0.549 | 0.554 | 75566 | 1269 | 0.527 | 0.001 | 0.525 | 0.529 |
| 12.2 | 72896 | 1130 | 0.543 | 0.001 | 0.541 | 0.546 | 73471 | 1171 | 0.519 | 0.001 | 0.516 | 0.521 |
| 12.3 | 70768 | 1737 | 0.530 | 0.001 | 0.528 | 0.532 | 71303 | 1730 | 0.506 | 0.001 | 0.504 | 0.508 |
| 12.4 | 68062 | 1164 | 0.521 | 0.001 | 0.518 | 0.523 | 68637 | 1100 | 0.498 | 0.001 | 0.496 | 0.500 |
| 12.5 | 66068 | 1218 | 0.511 | 0.001 | 0.509 | 0.514 | 66618 | 1315 | 0.488 | 0.001 | 0.486 | 0.491 |
| 12.6 | 64098 | 1126 | 0.502 | 0.001 | 0.500 | 0.505 | 64467 | 1221 | 0.479 | 0.001 | 0.477 | 0.481 |
| 12.7 | 62241 | 1099 | 0.493 | 0.001 | 0.491 | 0.496 | 62542 | 1119 | 0.470 | 0.001 | 0.468 | 0.473 |
| 12.8 | 60446 | 1694 | 0.480 | 0.001 | 0.477 | 0.482 | 60680 | 1678 | 0.457 | 0.001 | 0.455 | 0.460 |
| 12.9 | 57916 | 1109 | 0.470 | 0.001 | 0.468 | 0.473 | 58154 | 1119 | 0.449 | 0.001 | 0.446 | 0.451 |
| 13 | 55932 | 2254 | 0.451 | 0.001 | 0.449 | 0.454 | 56264 | 2268 | 0.430 | 0.001 | 0.428 | 0.433 |
| 13.1 | 52714 | 1201 | 0.441 | 0.001 | 0.439 | 0.444 | 53039 | 1311 | 0.420 | 0.001 | 0.417 | 0.422 |
| 13.2 | 50718 | 1182 | 0.431 | 0.001 | 0.428 | 0.433 | 50963 | 1183 | 0.410 | 0.001 | 0.408 | 0.413 |
| 13.3 | 48699 | 1639 | 0.416 | 0.001 | 0.414 | 0.419 | 48919 | 1686 | 0.396 | 0.001 | 0.393 | 0.398 |
| 13.4 | 46178 | 1111 | 0.406 | 0.001 | 0.404 | 0.409 | 46364 | 1154 | 0.386 | 0.001 | 0.384 | 0.389 |
| 13.5 | 44270 | 1196 | 0.395 | 0.001 | 0.393 | 0.398 | 44340 | 1289 | 0.375 | 0.001 | 0.372 | 0.377 |
| 13.6 | 42368 | 1119 | 0.385 | 0.001 | 0.382 | 0.388 | 42313 | 1177 | 0.364 | 0.001 | 0.362 | 0.367 |
| 13.7 | 40563 | 980 | 0.376 | 0.001 | 0.373 | 0.378 | 40459 | 1024 | 0.355 | 0.001 | 0.353 | 0.358 |
| 13.8 | 38968 | 1490 | 0.361 | 0.001 | 0.359 | 0.364 | 38778 | 1557 | 0.341 | 0.001 | 0.338 | 0.343 |
| 13.9 | 36940 | 969 | 0.352 | 0.001 | 0.349 | 0.354 | 36704 | 945 | 0.332 | 0.001 | 0.330 | 0.335 |
| 14 | 35668 | 2107 | 0.331 | 0.001 | 0.328 | 0.334 | 35461 | 2102 | 0.312 | 0.001 | 0.310 | 0.315 |
| 14.1 | 33240 | 1000 | 0.321 | 0.001 | 0.319 | 0.324 | 33083 | 1051 | 0.303 | 0.001 | 0.300 | 0.305 |
| 14.2 | 32037 | 952 | 0.312 | 0.001 | 0.309 | 0.314 | 31847 | 944 | 0.294 | 0.001 | 0.291 | 0.296 |
| 14.3 | 30918 | 1419 | 0.297 | 0.001 | 0.295 | 0.300 | 30749 | 1343 | 0.281 | 0.001 | 0.278 | 0.283 |
| 14.4 | 29289 | 844 | 0.289 | 0.001 | 0.286 | 0.291 | 29205 | 882 | 0.272 | 0.001 | 0.270 | 0.275 |
| 14.5 | 28295 | 951 | 0.279 | 0.001 | 0.276 | 0.282 | 28192 | 922 | 0.263 | 0.001 | 0.261 | 0.266 |
| 14.6 | 27176 | 822 | 0.271 | 0.001 | 0.268 | 0.273 | 27123 | 874 | 0.255 | 0.001 | 0.253 | 0.257 |
| 14.7 | 26208 | 784 | 0.262 | 0.001 | 0.260 | 0.265 | 26089 | 785 | 0.247 | 0.001 | 0.245 | 0.250 |
| 14.8 | 25313 | 1170 | 0.250 | 0.001 | 0.248 | 0.253 | 25176 | 1177 | 0.236 | 0.001 | 0.233 | 0.238 |
| 14.9 | 23994 | 784 | 0.242 | 0.001 | 0.240 | 0.245 | 23838 | 700 | 0.229 | 0.001 | 0.226 | 0.231 |
| 15 | 23078 | 1647 | 0.225 | 0.001 | 0.222 | 0.227 | 23001 | 1719 | 0.212 | 0.001 | 0.209 | 0.214 |
| 15.1 | 21262 | 785 | 0.217 | 0.001 | 0.214 | 0.219 | 21098 | 767 | 0.204 | 0.001 | 0.202 | 0.206 |
| 15.2 | 20355 | 696 | 0.209 | 0.001 | 0.207 | 0.212 | 20203 | 684 | 0.197 | 0.001 | 0.195 | 0.199 |
| 15.3 | 19549 | 994 | 0.199 | 0.001 | 0.196 | 0.201 | 19420 | 986 | 0.187 | 0.001 | 0.185 | 0.189 |
| 15.4 | 18438 | 609 | 0.192 | 0.001 | 0.190 | 0.194 | 18295 | 632 | 0.181 | 0.001 | 0.178 | 0.183 |
| 15.5 | 17709 | 659 | 0.185 | 0.001 | 0.183 | 0.187 | 17556 | 692 | 0.173 | 0.001 | 0.171 | 0.176 |
| 15.6 | 16948 | 596 | 0.178 | 0.001 | 0.176 | 0.181 | 16750 | 587 | 0.167 | 0.001 | 0.165 | 0.170 |
| 15.7 | 16244 | 605 | 0.172 | 0.001 | 0.169 | 0.174 | 16045 | 525 | 0.162 | 0.001 | 0.160 | 0.164 |
| 15.8 | 15547 | 810 | 0.163 | 0.001 | 0.161 | 0.165 | 15431 | 744 | 0.154 | 0.001 | 0.152 | 0.156 |
| 15.9 | 14607 | 512 | 0.157 | 0.001 | 0.155 | 0.159 | 14546 | 548 | 0.148 | 0.001 | 0.146 | 0.150 |
| 16 | 14003 | 1177 | 0.144 | 0.001 | 0.142 | 0.146 | 13915 | 1199 | 0.136 | 0.001 | 0.134 | 0.138 |
| 16.1 | 12710 | 543 | 0.138 | 0.001 | 0.136 | 0.140 | 12595 | 511 | 0.130 | 0.001 | 0.128 | 0.132 |
| 16.2 | 12080 | 464 | 0.132 | 0.001 | 0.130 | 0.134 | 11989 | 441 | 0.125 | 0.001 | 0.123 | 0.127 |
| 16.3 | 11557 | 596 | 0.126 | 0.001 | 0.124 | 0.128 | 11482 | 591 | 0.119 | 0.001 | 0.117 | 0.121 |
| 16.4 | 10866 | 401 | 0.121 | 0.001 | 0.119 | 0.123 | 10805 | 341 | 0.115 | 0.001 | 0.113 | 0.117 |
| 16.5 | 10400 | 420 | 0.116 | 0.001 | 0.114 | 0.118 | 10380 | 419 | 0.110 | 0.001 | 0.109 | 0.112 |
| 16.6 | 9902 | 361 | 0.112 | 0.001 | 0.110 | 0.114 | 9879 | 380 | 0.106 | 0.001 | 0.104 | 0.108 |
| 16.7 | 9477 | 337 | 0.108 | 0.001 | 0.106 | 0.110 | 9429 | 318 | 0.103 | 0.001 | 0.101 | 0.104 |
| 16.8 | 9084 | 424 | 0.103 | 0.001 | 0.101 | 0.105 | 9036 | 430 | 0.098 | 0.001 | 0.096 | 0.100 |
| 16.9 | 8582 | 289 | 0.099 | 0.001 | 0.097 | 0.101 | 8538 | 266 | 0.095 | 0.001 | 0.093 | 0.096 |
| 17 | 8226 | 724 | 0.091 | 0.001 | 0.089 | 0.092 | 8209 | 695 | 0.087 | 0.001 | 0.085 | 0.088 |
| 17.1 | 7426 | 301 | 0.087 | 0.001 | 0.085 | 0.089 | 7419 | 265 | 0.084 | 0.001 | 0.082 | 0.085 |
| 17.2 | 7070 | 273 | 0.084 | 0.001 | 0.082 | 0.085 | 7099 | 249 | 0.081 | 0.001 | 0.079 | 0.082 |
| 17.3 | 6747 | 350 | 0.079 | 0.001 | 0.078 | 0.081 | 6808 | 322 | 0.077 | 0.001 | 0.075 | 0.078 |
| 17.4 | 6333 | 232 | 0.076 | 0.001 | 0.075 | 0.078 | 6421 | 196 | 0.074 | 0.001 | 0.073 | 0.076 |
| 17.5 | 6060 | 246 | 0.073 | 0.001 | 0.072 | 0.075 | 6163 | 211 | 0.072 | 0.001 | 0.070 | 0.074 |
| 17.6 | 5778 | 212 | 0.071 | 0.001 | 0.069 | 0.072 | 5911 | 189 | 0.070 | 0.001 | 0.068 | 0.071 |
| 17.7 | 5508 | 191 | 0.068 | 0.001 | 0.066 | 0.070 | 5678 | 183 | 0.067 | 0.001 | 0.066 | 0.069 |
| 17.8 | 5290 | 245 | 0.065 | 0.001 | 0.063 | 0.067 | 5454 | 254 | 0.064 | 0.001 | 0.063 | 0.066 |
| 17.9 | 4989 | 168 | 0.063 | 0.001 | 0.061 | 0.064 | 5140 | 171 | 0.062 | 0.001 | 0.061 | 0.064 |
| 18 | 4788 | 403 | 0.057 | 0.001 | 0.056 | 0.059 | 4921 | 435 | 0.057 | 0.001 | 0.055 | 0.058 |
| 18.1 | 4329 | 192 | 0.055 | 0.001 | 0.053 | 0.056 | 4417 | 171 | 0.054 | 0.001 | 0.053 | 0.056 |
| 18.2 | 4095 | 142 | 0.053 | 0.001 | 0.052 | 0.055 | 4203 | 145 | 0.053 | 0.001 | 0.051 | 0.054 |
| 18.3 | 3922 | 192 | 0.050 | 0.001 | 0.049 | 0.052 | 4023 | 184 | 0.050 | 0.001 | 0.049 | 0.052 |
| 18.4 | 3694 | 119 | 0.049 | 0.001 | 0.047 | 0.050 | 3786 | 153 | 0.048 | 0.001 | 0.047 | 0.050 |
| 18.5 | 3549 | 119 | 0.047 | 0.001 | 0.046 | 0.049 | 3602 | 142 | 0.046 | 0.001 | 0.045 | 0.048 |
| 18.6 | 3396 | 121 | 0.045 | 0.001 | 0.044 | 0.047 | 3420 | 129 | 0.044 | 0.001 | 0.043 | 0.046 |
| 18.7 | 3244 | 101 | 0.044 | 0.001 | 0.043 | 0.045 | 3250 | 109 | 0.043 | 0.001 | 0.042 | 0.044 |
| 18.8 | 3116 | 138 | 0.042 | 0.001 | 0.041 | 0.044 | 3112 | 115 | 0.041 | 0.001 | 0.040 | 0.043 |
| 18.9 | 2935 | 115 | 0.040 | 0.001 | 0.039 | 0.042 | 2961 | 124 | 0.040 | 0.001 | 0.038 | 0.041 |
| 19 | 2799 | 177 | 0.038 | 0.001 | 0.037 | 0.039 | 2803 | 153 | 0.037 | 0.001 | 0.036 | 0.039 |
| 19.1 | 2576 | 90 | 0.037 | 0.001 | 0.035 | 0.038 | 2595 | 110 | 0.036 | 0.001 | 0.035 | 0.037 |
| 19.2 | 2461 | 75 | 0.035 | 0.001 | 0.034 | 0.037 | 2458 | 71 | 0.035 | 0.001 | 0.034 | 0.036 |
| 19.3 | 2366 | 105 | 0.034 | 0.001 | 0.033 | 0.035 | 2362 | 94 | 0.033 | 0.001 | 0.032 | 0.035 |
| 19.4 | 2234 | 81 | 0.033 | 0.001 | 0.031 | 0.034 | 2242 | 74 | 0.032 | 0.001 | 0.031 | 0.034 |
| 19.5 | 2129 | 68 | 0.032 | 0.001 | 0.030 | 0.033 | 2149 | 62 | 0.031 | 0.001 | 0.030 | 0.033 |
| 19.6 | 2023 | 83 | 0.030 | 0.001 | 0.029 | 0.032 | 2059 | 69 | 0.030 | 0.001 | 0.029 | 0.032 |
| 19.7 | 1918 | 49 | 0.030 | 0.001 | 0.028 | 0.031 | 1964 | 57 | 0.030 | 0.001 | 0.028 | 0.031 |
| 19.8 | 1852 | 62 | 0.029 | 0.001 | 0.027 | 0.030 | 1891 | 73 | 0.028 | 0.001 | 0.027 | 0.030 |
| 19.9 | 1770 | 63 | 0.028 | 0.001 | 0.026 | 0.029 | 1803 | 53 | 0.028 | 0.001 | 0.026 | 0.029 |
| 20 | 1687 | 105 | 0.026 | 0.001 | 0.025 | 0.027 | 1728 | 97 | 0.026 | 0.001 | 0.025 | 0.027 |
| 20.1 | 1557 | 47 | 0.025 | 0.001 | 0.024 | 0.026 | 1592 | 51 | 0.025 | 0.001 | 0.024 | 0.026 |
| 20.2 | 1486 | 48 | 0.024 | 0.001 | 0.023 | 0.025 | 1530 | 56 | 0.024 | 0.001 | 0.023 | 0.025 |
| 20.3 | 1426 | 47 | 0.023 | 0.001 | 0.022 | 0.025 | 1462 | 55 | 0.023 | 0.001 | 0.022 | 0.024 |
| 20.4 | 1368 | 45 | 0.023 | 0.001 | 0.022 | 0.024 | 1387 | 50 | 0.022 | 0.001 | 0.021 | 0.024 |
| 20.5 | 1310 | 34 | 0.022 | 0.001 | 0.021 | 0.023 | 1327 | 31 | 0.022 | 0.001 | 0.021 | 0.023 |
| 20.6 | 1265 | 61 | 0.021 | 0.001 | 0.020 | 0.022 | 1282 | 48 | 0.021 | 0.001 | 0.020 | 0.022 |
| 20.7 | 1193 | 32 | 0.020 | 0.001 | 0.019 | 0.022 | 1212 | 41 | 0.020 | 0.001 | 0.019 | 0.021 |
| 20.8 | 1153 | 41 | 0.020 | 0.001 | 0.019 | 0.021 | 1162 | 39 | 0.020 | 0.001 | 0.019 | 0.021 |
| 20.9 | 1096 | 35 | 0.019 | 0.001 | 0.018 | 0.020 | 1112 | 35 | 0.019 | 0.001 | 0.018 | 0.020 |
| 21 | 1051 | 49 | 0.018 | 0.001 | 0.017 | 0.019 | 1069 | 49 | 0.018 | 0.001 | 0.017 | 0.019 |
| 21.1 | 987 | 43 | 0.017 | 0.001 | 0.016 | 0.018 | 1001 | 44 | 0.017 | 0.000 | 0.016 | 0.018 |
| 21.2 | 936 | 31 | 0.017 | 0.000 | 0.016 | 0.018 | 944 | 33 | 0.017 | 0.000 | 0.016 | 0.018 |
| 21.3 | 896 | 37 | 0.016 | 0.000 | 0.015 | 0.017 | 893 | 30 | 0.016 | 0.000 | 0.015 | 0.017 |
| 21.4 | 846 | 29 | 0.016 | 0.000 | 0.015 | 0.017 | 854 | 30 | 0.016 | 0.000 | 0.015 | 0.017 |
| 21.5 | 808 | 23 | 0.015 | 0.000 | 0.014 | 0.016 | 817 | 25 | 0.015 | 0.000 | 0.014 | 0.016 |
| 21.6 | 778 | 40 | 0.014 | 0.000 | 0.013 | 0.015 | 779 | 32 | 0.015 | 0.000 | 0.014 | 0.016 |
| 21.7 | 730 | 27 | 0.014 | 0.000 | 0.013 | 0.015 | 735 | 29 | 0.014 | 0.000 | 0.013 | 0.015 |
| 21.8 | 695 | 23 | 0.013 | 0.000 | 0.013 | 0.014 | 697 | 26 | 0.013 | 0.000 | 0.013 | 0.014 |
| 21.9 | 666 | 35 | 0.013 | 0.000 | 0.012 | 0.014 | 655 | 29 | 0.013 | 0.000 | 0.012 | 0.014 |
| 22 | 619 | 37 | 0.012 | 0.000 | 0.011 | 0.013 | 615 | 37 | 0.012 | 0.000 | 0.011 | 0.013 |
| 22.1 | 562 | 47 | 0.011 | 0.000 | 0.010 | 0.012 | 566 | 47 | 0.011 | 0.000 | 0.010 | 0.012 |
| 22.2 | 509 | 17 | 0.011 | 0.000 | 0.010 | 0.011 | 509 | 15 | 0.011 | 0.000 | 0.010 | 0.012 |
| 22.3 | 488 | 22 | 0.010 | 0.000 | 0.009 | 0.011 | 490 | 16 | 0.010 | 0.000 | 0.010 | 0.011 |
| 22.4 | 459 | 24 | 0.010 | 0.000 | 0.009 | 0.010 | 471 | 26 | 0.010 | 0.000 | 0.009 | 0.011 |
| 22.5 | 429 | 17 | 0.009 | 0.000 | 0.008 | 0.010 | 439 | 26 | 0.009 | 0.000 | 0.009 | 0.010 |
| 22.6 | 404 | 27 | 0.009 | 0.000 | 0.008 | 0.009 | 411 | 21 | 0.009 | 0.000 | 0.008 | 0.010 |
| 22.7 | 372 | 26 | 0.008 | 0.000 | 0.007 | 0.009 | 380 | 18 | 0.008 | 0.000 | 0.008 | 0.009 |
| 22.8 | 343 | 21 | 0.007 | 0.000 | 0.007 | 0.008 | 360 | 17 | 0.008 | 0.000 | 0.007 | 0.009 |
| 22.9 | 313 | 25 | 0.007 | 0.000 | 0.006 | 0.008 | 339 | 20 | 0.008 | 0.000 | 0.007 | 0.008 |
| 23 | 285 | 22 | 0.006 | 0.000 | 0.006 | 0.007 | 317 | 33 | 0.007 | 0.000 | 0.006 | 0.007 |
| 23.1 | 253 | 26 | 0.006 | 0.000 | 0.005 | 0.006 | 268 | 37 | 0.006 | 0.000 | 0.005 | 0.007 |
| 23.2 | 218 | 12 | 0.005 | 0.000 | 0.005 | 0.006 | 225 | 18 | 0.005 | 0.000 | 0.005 | 0.006 |
| 23.3 | 205 | 20 | 0.005 | 0.000 | 0.004 | 0.006 | 204 | 16 | 0.005 | 0.000 | 0.004 | 0.006 |
| 23.4 | 182 | 14 | 0.004 | 0.000 | 0.004 | 0.005 | 185 | 16 | 0.004 | 0.000 | 0.004 | 0.005 |
| 23.5 | 168 | 23 | 0.004 | 0.000 | 0.003 | 0.004 | 162 | 15 | 0.004 | 0.000 | 0.004 | 0.005 |
| 23.6 | 140 | 18 | 0.003 | 0.000 | 0.003 | 0.004 | 142 | 24 | 0.003 | 0.000 | 0.003 | 0.004 |
| 23.7 | 119 | 19 | 0.003 | 0.000 | 0.002 | 0.003 | 114 | 22 | 0.003 | 0.000 | 0.002 | 0.003 |
| 23.8 | 97 | 15 | 0.002 | 0.000 | 0.002 | 0.003 | 87 | 17 | 0.002 | 0.000 | 0.002 | 0.003 |
| 23.9 | 74 | 19 | 0.002 | 0.000 | 0.001 | 0.002 | 58 | 16 | 0.002 | 0.000 | 0.001 | 0.002 |
| 24 | 52 | 38 | 0.000 | 0.000 | 0.000 | 0.001 | 39 | 25 | 0.001 | 0.000 | 0.000 | 0.001 |
