# Supplementary material for: Longevity of companion dog breeds: those at risk from early death
Source: Sci Rep. 2024 Feb 1;14:531. doi: 10.1038/s41598-023-50458-w (PMC10834484; doi:10.1038/s41598-023-50458-w)
Supplement: Supplementary file 8 — Supplementary Table 3. [file 41598_2023_50458_MOESM8_ESM.docx]

***Table S3:*** *Kaplan-Meier survival estimates and cox proportional hazards regression model outputs for 155 recognised purebred versus the crossbred group (n = 1). Includes the following statistics per* ***Breed****:* $\boldsymbol{N}_{\boldsymbol{A}}$ *i.e., total number of individuals still alive;* $\boldsymbol{N}_{\boldsymbol{D}}$ *i.e., total number of deaths;* ***Median Survival*** *i.e., median age of death;* ***Lower 95% Confidence Interval (CI)*** *and* ***Upper 95% CI****;* ***Hazards Ratio*** *(Lower 95% CI and Upper 95% CI) and* ***p-value****.*

| **Breed** | $\boldsymbol{N}_{\boldsymbol{A}}$ | $\boldsymbol{N}_{\boldsymbol{D}}$ | **Median Survival** | ***Lower 95% CI*** | ***Upper 95% CI*** | **HR (95% CI)** | **p-value** |
| --- | --- | --- | --- | --- | --- | --- | --- |
| Crossbreed | 111053 | 72445 | 12 | 12 | 12.1 | — | — |
| Affenpinscher | 338 | 204 | 9.3 | 7.4 | 10.8 | 1.7 (1.48, 1.94) | <0.001 |
| Afghan Hound | 380 | 238 | 11.1 | 10.5 | 11.8 | 1.35 (1.19, 1.53) | <0.001 |
| Airedale Terrier | 1270 | 556 | 12 | 11.8 | 12.3 | 1.04 (0.96, 1.13) | 0.31 |
| Akita | 3828 | 2358 | 11.4 | 11.1 | 11.6 | 1.29 (1.24, 1.34) | <0.001 |
| Alaskan Malamute | 1980 | 842 | 11.3 | 11.1 | 11.8 | 1.35 (1.26, 1.44) | <0.001 |
| American Cocker Spaniel | 657 | 293 | 13.3 | 13 | 13.7 | 0.75 (0.67, 0.84) | <0.001 |
| American Eskimo Dog | 36 | 22 | 11.3 | 10 | 12 | 1.42 (0.94, 2.16) | 0.10 |
| American Staffordshire Terrier | 142 | 94 | 12.5 | 11.4 | 13.1 | 1.09 (0.89, 1.33) | 0.40 |
| Anatolian Shepherd | 70 | 40 | 10.1 | 7.6 | 11.2 | 1.53 (1.12, 2.08) | <0.01 |
| Australian Cattle Dog | 117 | 50 | 14 | 13.2 | 15.1 | 0.59 (0.45, 0.78) | <0.001 |
| Australian Shepherd | 224 | 62 | 13.7 | 12.8 | 14.6 | 0.69 (0.54, 0.88) | <0.01 |
| Basenji | 92 | 30 | 12.1 | 11.3 | 15.8 | 0.88 (0.61, 1.25) | 0.47 |
| Basset Hound | 2013 | 883 | 12.5 | 12.2 | 12.7 | 0.9 (0.84, 0.96) | <0.01 |
| Beagle | 4678 | 1552 | 12.5 | 12.3 | 12.7 | 0.95 (0.91, 1) | 0.07 |
| Bearded Collie | 1165 | 570 | 13.9 | 13.7 | 14 | 0.64 (0.59, 0.69) | <0.001 |
| Bedlington Terrier | 1294 | 549 | 13.7 | 13.4 | 14 | 0.67 (0.61, 0.73) | <0.001 |
| Belgian Malenois | 606 | 337 | 12 | 11.5 | 12.6 | 1.06 (0.96, 1.18) | 0.25 |
| Belgian Tervuren | 208 | 55 | 13.8 | 12.7 | 14.8 | 0.7 (0.54, 0.92) | <0.01 |
| Bernese Mountain Dog | 1116 | 449 | 10.1 | 9.8 | 10.4 | 1.42 (1.3, 1.56) | <0.001 |
| Bichon Frise | 5902 | 2900 | 12.5 | 12.3 | 12.7 | 0.92 (0.89, 0.96) | <0.001 |
| Black Russian Terrier | 163 | 71 | 10.9 | 10.2 | 12.3 | 1.32 (1.05, 1.67) | 0.02 |
| Bloodhound | 134 | 71 | 9.3 | 8.6 | 11 | 1.68 (1.33, 2.12) | <0.001 |
| Bolognese | 182 | 32 | 14.9 | 14 | NA | 0.49 (0.35, 0.7) | <0.001 |
| Border Collie | 12917 | 7969 | 13.1 | 13 | 13.2 | 0.85 (0.83, 0.87) | <0.001 |
| Border Terrier | 9387 | 2427 | 14.2 | 14.1 | 14.3 | 0.53 (0.51, 0.55) | <0.001 |
| Borzoi | 142 | 43 | 12 | 10.8 | NA | 0.88 (0.65, 1.19) | 0.40 |
| Boston Terrier | 1984 | 443 | 11.8 | 11 | 12.2 | 1.3 (1.18, 1.42) | <0.001 |
| Bouvier des Flandres | 151 | 79 | 11.3 | 10.4 | 11.9 | 1.07 (0.86, 1.34) | 0.52 |
| Boxer | 11294 | 5925 | 11.3 | 11.3 | 11.5 | 1.18 (1.15, 1.21) | <0.001 |
| Bracco Italiano | 106 | 22 | 13.8 | 11.8 | NA | 0.95 (0.63, 1.44) | 0.81 |
| Briard | 201 | 76 | 12.6 | 11.3 | 13.4 | 0.82 (0.66, 1.03) | 0.09 |
| Brittany | 991 | 734 | 11.1 | 10.9 | 11.3 | 1.49 (1.38, 1.6) | <0.001 |
| Brussels Griffon | 315 | 102 | 13.3 | 12.7 | 13.9 | 0.82 (0.67, 0.99) | 0.04 |
| Bull Terrier | 4503 | 2151 | 12 | 11.9 | 12.2 | 1.04 (1, 1.09) | 0.07 |
| Bulldog | 11065 | 4743 | 9.8 | 9.7 | 10 | 1.78 (1.73, 1.83) | <0.001 |
| Bullmastiff | 2188 | 1169 | 10.2 | 9.9 | 10.5 | 1.31 (1.24, 1.39) | <0.001 |
| Cairn Terrier | 2585 | 1139 | 14 | 13.8 | 14.3 | 0.6 (0.57, 0.64) | <0.001 |
| Canaan Dog | 45 | 28 | 12 | 9.5 | 14.7 | 1.07 (0.74, 1.54) | 0.74 |
| Cane Corso | 202 | 101 | 8.1 | 6.5 | 9.5 | 2.44 (2.01, 2.97) | <0.001 |
| Cardigan Welsh Corgi | 203 | 69 | 13.1 | 12.5 | 14.4 | 0.69 (0.55, 0.88) | <0.01 |
| Caucasian Shepherd Dog | 52 | 22 | 5.4 | 4.6 | NA | 2.44 (1.61, 3.71) | <0.001 |
| Cavalier King Charles Spaniel | 13782 | 6879 | 11.8 | 11.7 | 11.9 | 1.07 (1.04, 1.09) | <0.001 |
| Chesapeake Bay Retriever | 126 | 42 | 11.6 | 10.5 | NA | 1.16 (0.85, 1.56) | 0.35 |
| Chihuahua | 10788 | 3848 | 11.8 | 11.5 | 11.9 | 1.17 (1.13, 1.21) | <0.001 |
| Chinese Crested | 928 | 394 | 13.4 | 13 | 13.8 | 0.77 (0.7, 0.85) | <0.001 |
| Chinese Shar Pei | 4326 | 2028 | 10.6 | 10.3 | 10.9 | 1.46 (1.39, 1.52) | <0.001 |
| Chow Chow | 940 | 395 | 12.1 | 11.7 | 12.5 | 1.05 (0.95, 1.16) | 0.36 |
| Clumber Spaniel | 300 | 85 | 12.3 | 11.7 | 13.9 | 0.99 (0.8, 1.23) | 0.94 |
| Collie | 2171 | 1104 | 13.3 | 13.3 | 13.6 | 0.72 (0.68, 0.76) | <0.001 |
| Coton de Tulear | 319 | 44 | 14.2 | 13.1 | NA | 0.63 (0.47, 0.85) | <0.01 |
| Curly Coated Retriever | 152 | 66 | 12.2 | 11.2 | 12.8 | 0.96 (0.75, 1.22) | 0.74 |
| Dachshund | 2305 | 926 | 13.2 | 13 | 13.5 | 0.77 (0.72, 0.83) | <0.001 |
| Dalmatian | 3126 | 1443 | 13.2 | 13 | 13.3 | 0.77 (0.73, 0.81) | <0.001 |
| Dandie Dinmont Terrier | 179 | 66 | 12.8 | 12.2 | 14.6 | 0.86 (0.68, 1.1) | 0.23 |
| Doberman Pinscher | 3531 | 1623 | 11.2 | 11 | 11.5 | 1.14 (1.08, 1.2) | <0.001 |
| Dogue de Bordeaux | 3427 | 1204 | 11.1 | 10.6 | 11.7 | 1.4 (1.32, 1.48) | <0.001 |
| English Cocker Spaniel | 26303 | 7584 | 13.3 | 13.2 | 13.3 | 0.75 (0.74, 0.77) | <0.001 |
| English Setter | 596 | 238 | 13.1 | 12.7 | 13.6 | 0.7 (0.62, 0.8) | <0.001 |
| English Springer Spaniel | 17905 | 6221 | 13.5 | 13.4 | 13.6 | 0.7 (0.68, 0.72) | <0.001 |
| Field Spaniel | 242 | 136 | 13.1 | 12.5 | 13.4 | 0.83 (0.7, 0.98) | 0.03 |
| Finnish Lapphund | 105 | 23 | 13.8 | 13.5 | NA | 0.77 (0.51, 1.16) | 0.21 |
| Flat Coated Retriever | 2148 | 784 | 11.7 | 11.5 | 11.9 | 1.08 (1.01, 1.16) | 0.03 |
| Foxhound | 71 | 31 | 13 | 11.4 | 16.9 | 0.67 (0.47, 0.95) | 0.02 |
| French Bulldog | 11821 | 2495 | 9.8 | 9.4 | 10.1 | 2.22 (2.13, 2.31) | <0.001 |
| German Shepherd Dog | 19908 | 10773 | 11.3 | 11.3 | 11.4 | 1.24 (1.22, 1.27) | <0.001 |
| German Shorthaired Pointer | 3313 | 947 | 13.4 | 13.3 | 13.6 | 0.67 (0.63, 0.72) | <0.001 |
| German Spitz Mittel | 211 | 96 | 14 | 13.3 | 14.6 | 0.59 (0.49, 0.73) | <0.001 |
| German Wirehaired Pointer | 562 | 138 | 13 | 12.5 | 13.6 | 0.75 (0.64, 0.89) | <0.001 |
| Giant Schnauzer | 369 | 126 | 12.1 | 11.5 | 12.8 | 0.92 (0.77, 1.09) | 0.33 |
| Glen Of Imaal Terrier | 113 | 40 | 12.1 | 11.6 | 13.5 | 1.06 (0.78, 1.44) | 0.72 |
| Golden Retriever | 11506 | 4252 | 13.2 | 13.1 | 13.2 | 0.76 (0.74, 0.78) | <0.001 |
| Gordon Setter | 393 | 138 | 12.4 | 12.1 | 12.8 | 0.85 (0.72, 1.01) | 0.06 |
| Great Dane | 1986 | 864 | 10.6 | 10.4 | 11 | 1.2 (1.13, 1.29) | <0.001 |
| Great Pyrenees | 260 | 128 | 10.9 | 10.5 | 11.3 | 1.5 (1.26, 1.79) | <0.001 |
| Greyhound | 3964 | 3025 | 11.5 | 11.4 | 11.6 | 1.19 (1.14, 1.23) | <0.001 |
| Havanese | 381 | 56 | 14.5 | 14.1 | NA | 0.72 (0.56, 0.94) | 0.02 |
| Irish Setter | 1487 | 547 | 12.9 | 12.6 | 13.1 | 0.74 (0.68, 0.8) | <0.001 |
| Irish Terrier | 535 | 146 | 13.5 | 13.1 | 14 | 0.65 (0.55, 0.76) | <0.001 |
| Irish Water Spaniel | 174 | 68 | 10.8 | 10.4 | 11.9 | 1.33 (1.05, 1.68) | 0.02 |
| Irish Wolfhound | 509 | 210 | 9.9 | 9.2 | 11 | 1.53 (1.34, 1.75) | <0.001 |
| Italian Greyhound | 389 | 70 | 14 | 13.2 | 15.1 | 0.71 (0.56, 0.89) | <0.01 |
| Jack Russell Terrier | 19166 | 13497 | 13.3 | 13.2 | 13.3 | 0.79 (0.77, 0.8) | <0.001 |
| Japanese Chin | 373 | 142 | 12.5 | 12.2 | 13.3 | 0.88 (0.75, 1.04) | 0.13 |
| Japanese Spitz | 336 | 117 | 13 | 12.3 | 13.7 | 0.86 (0.72, 1.03) | 0.10 |
| Keeshond | 171 | 55 | 12.3 | 11.6 | 13.8 | 0.84 (0.65, 1.1) | 0.21 |
| Kelpie | 97 | 49 | 12 | 11.6 | 13.5 | 0.97 (0.74, 1.29) | 0.86 |
| Kerry Blue Terrier | 368 | 168 | 12.4 | 11.9 | 13 | 0.93 (0.8, 1.08) | 0.31 |
| Labrador Retriever | 43428 | 16155 | 13.1 | 13 | 13.1 | 0.8 (0.78, 0.81) | <0.001 |
| Lakeland Terrier | 1068 | 601 | 14.2 | 13.9 | 14.5 | 0.66 (0.61, 0.71) | <0.001 |
| Lancashire Heeler | 241 | 110 | 15.4 | 14.7 | 15.9 | 0.49 (0.41, 0.59) | <0.001 |
| Large Munsterlander | 139 | 26 | 14.1 | 13.5 | NA | 0.47 (0.32, 0.69) | <0.001 |
| Leonberger | 676 | 321 | 10 | 9.5 | 10.3 | 1.61 (1.44, 1.79) | <0.001 |
| Lhasa Apso | 7216 | 2723 | 14 | 13.9 | 14.2 | 0.62 (0.59, 0.64) | <0.001 |
| Lowchen | 143 | 50 | 13.9 | 13.3 | 15.7 | 0.59 (0.45, 0.78) | <0.001 |
| Maltese | 2021 | 666 | 13.1 | 12.6 | 13.5 | 0.93 (0.86, 1) | 0.06 |
| Mastiff | 1840 | 1419 | 9 | 8.9 | 9.2 | 2.32 (2.2, 2.44) | <0.001 |
| Miniature Bull Terrier | 268 | 94 | 12.2 | 11 | 13.8 | 1.02 (0.83, 1.25) | 0.86 |
| Miniature Dachshund | 7342 | 1655 | 14 | 13.7 | 14.2 | 0.65 (0.62, 0.69) | <0.001 |
| Miniature Pinscher | 660 | 167 | 13.7 | 13.1 | 14.2 | 0.75 (0.64, 0.87) | <0.001 |
| Miniature Schnauzer | 7693 | 1977 | 13.3 | 13.2 | 13.5 | 0.7 (0.67, 0.73) | <0.001 |
| Neapolitan Mastiff | 367 | 210 | 9.3 | 8.8 | 10.2 | 1.77 (1.55, 2.03) | <0.001 |
| Newfoundland | 1529 | 633 | 11 | 10.9 | 11.6 | 1.2 (1.11, 1.3) | <0.001 |
| Norfolk Terrier | 905 | 297 | 13.5 | 13.1 | 13.8 | 0.71 (0.63, 0.79) | <0.001 |
| Norwegian Elkhound | 145 | 64 | 12.9 | 12.4 | 14.1 | 0.78 (0.61, 1) | 0.05 |
| Norwich Terrier | 190 | 45 | 14 | 13.5 | 15.1 | 0.56 (0.42, 0.75) | <0.001 |
| Nova Scotia Duck Tolling Retriever | 286 | 47 | 13.2 | 12.2 | NA | 0.65 (0.49, 0.87) | <0.01 |
| Old English Sheepdog | 1210 | 604 | 12.1 | 11.9 | 12.5 | 0.98 (0.9, 1.06) | 0.61 |
| Papillon | 1115 | 427 | 14.5 | 14.1 | 14.9 | 0.57 (0.52, 0.63) | <0.001 |
| Parson Russell Terrier | 2600 | 1176 | 13.8 | 13.5 | 14 | 0.68 (0.64, 0.72) | <0.001 |
| Pekingese | 695 | 339 | 13.3 | 13 | 13.8 | 0.71 (0.64, 0.79) | <0.001 |
| Pembroke Welsh Corgi | 643 | 221 | 13.2 | 12.7 | 13.6 | 0.72 (0.63, 0.82) | <0.001 |
| Petite Basset Griffon Vendeen | 496 | 148 | 13.7 | 13.2 | 14 | 0.68 (0.58, 0.79) | <0.001 |
| Pharaoh Hound | 36 | 23 | 10 | 6.9 | 13.3 | 1.87 (1.24, 2.81) | <0.01 |
| Polish Lowland Sheepdog | 83 | 24 | 13.2 | 12.3 | 14.8 | 0.8 (0.53, 1.19) | 0.27 |
| Pomeranian | 2941 | 1170 | 12.2 | 12 | 12.5 | 1.03 (0.97, 1.09) | 0.37 |
| Poodle | 6427 | 2399 | 14 | 13.8 | 14.1 | 0.64 (0.61, 0.67) | <0.001 |
| Portuguese Water Dog | 179 | 36 | 13 | 11.5 | 14.9 | 0.85 (0.61, 1.18) | 0.32 |
| Presa Canario | 144 | 98 | 7.7 | 7 | 9.5 | 3.04 (2.49, 3.71) | <0.001 |
| Pug | 10026 | 2782 | 11.6 | 11.4 | 11.8 | 1.34 (1.29, 1.4) | <0.001 |
| Puli | 122 | 39 | 13.3 | 12.8 | 14.4 | 0.73 (0.53, 1) | 0.05 |
| Rhodesian Ridgeback | 2108 | 883 | 12 | 11.9 | 12.2 | 1 (0.94, 1.07) | 0.98 |
| Rottweiler | 6275 | 3442 | 10.6 | 10.4 | 10.7 | 1.27 (1.23, 1.32) | <0.001 |
| Saluki | 428 | 169 | 13.3 | 12.3 | 14 | 0.87 (0.75, 1.01) | 0.08 |
| Samoyed | 723 | 336 | 13.1 | 12.7 | 13.5 | 0.75 (0.67, 0.84) | <0.001 |
| Schipperke | 76 | 28 | 14.2 | 12.7 | 15.8 | 0.6 (0.42, 0.87) | <0.01 |
| Scottish Deerhound | 426 | 191 | 10.5 | 9.7 | 11.3 | 1.43 (1.24, 1.65) | <0.001 |
| Scottish Terrier | 1747 | 869 | 12.7 | 12.5 | 12.9 | 0.84 (0.79, 0.9) | <0.001 |
| Sealyham Terrier | 125 | 33 | 13.1 | 12.3 | 16 | 0.58 (0.41, 0.81) | <0.01 |
| Shetland Sheepdog | 1905 | 793 | 13.4 | 13.1 | 13.6 | 0.69 (0.64, 0.74) | <0.001 |
| Shiba Inu | 423 | 91 | 14.6 | 14 | 15.1 | 0.45 (0.37, 0.55) | <0.001 |
| Shih Tzu | 12007 | 5894 | 12.8 | 12.6 | 12.9 | 0.94 (0.91, 0.96) | <0.001 |
| Siberian Husky | 4453 | 1947 | 11.9 | 11.7 | 12 | 1.15 (1.1, 1.21) | <0.001 |
| Silky Terrier | 105 | 45 | 13.3 | 11.7 | 14.2 | 0.83 (0.62, 1.11) | 0.20 |
| Skye Terrier | 91 | 39 | 12.4 | 11.6 | 13.9 | 0.93 (0.68, 1.27) | 0.64 |
| Soft Coated Wheaten Terrier | 620 | 173 | 13.7 | 13.2 | 14.4 | 0.61 (0.53, 0.71) | <0.001 |
| Spanish Water Dog | 265 | 37 | 13.7 | 13.3 | NA | 0.51 (0.37, 0.7) | <0.001 |
| Spinone Italiano | 693 | 215 | 11.9 | 11.6 | 12.4 | 1 (0.88, 1.15) | 0.97 |
| St Bernard | 1129 | 509 | 9.3 | 9 | 9.8 | 1.66 (1.52, 1.81) | <0.001 |
| Staffordshire Bull Terrier | 36216 | 25332 | 12 | 12 | 12.1 | 1.13 (1.11, 1.14) | <0.001 |
| Standard Schnauzer | 826 | 435 | 13 | 12.7 | 13.2 | 0.82 (0.75, 0.9) | <0.001 |
| Sussex Spaniel | 108 | 34 | 13.5 | 12.6 | 15.1 | 0.79 (0.56, 1.1) | 0.16 |
| Swedish Valhund | 61 | 22 | 14 | 12.5 | NA | 0.65 (0.43, 0.99) | 0.04 |
| Tibetan Mastiff | 117 | 22 | 13.3 | 10.9 | NA | 0.95 (0.63, 1.45) | 0.83 |
| Tibetan Spaniel | 351 | 137 | 15.2 | 14.8 | 15.7 | 0.45 (0.38, 0.53) | <0.001 |
| Tibetan Terrier | 1947 | 583 | 13.8 | 13.5 | 14.2 | 0.58 (0.54, 0.63) | <0.001 |
| Toy Fox Terrier | 163 | 52 | 12.9 | 12.3 | 13.8 | 0.83 (0.63, 1.09) | 0.17 |
| Toy Manchester Terrier | 339 | 138 | 13 | 12 | 13.6 | 0.86 (0.73, 1.02) | 0.08 |
| Vizsla | 2965 | 503 | 13.5 | 13.2 | 13.9 | 0.67 (0.61, 0.73) | <0.001 |
| Weimaraner | 2990 | 1229 | 12.8 | 12.6 | 13 | 0.8 (0.75, 0.84) | <0.001 |
| Welsh Springer Spaniel | 572 | 162 | 14 | 13.3 | 14.3 | 0.6 (0.51, 0.7) | <0.001 |
| Welsh Terrier | 570 | 146 | 13.8 | 13.5 | 14.6 | 0.54 (0.46, 0.64) | <0.001 |
| West Highland White Terrier | 11932 | 6730 | 13.4 | 13.3 | 13.5 | 0.73 (0.72, 0.75) | <0.001 |
| Whippet | 4971 | 1483 | 13.4 | 13.2 | 13.7 | 0.7 (0.66, 0.73) | <0.001 |
| Wire Fox Terrier | 1413 | 521 | 13.5 | 13.3 | 13.7 | 0.73 (0.67, 0.8) | <0.001 |
| Yorkshire Terrier | 14579 | 9509 | 13.3 | 13.3 | 13.4 | 0.79 (0.77, 0.8) | <0.001 |
