# Supplementary material for: Longevity of companion dog breeds: those at risk from early death
Source: Sci Rep. 2024 Feb 1;14:531. doi: 10.1038/s41598-023-50458-w (PMC10834484; doi:10.1038/s41598-023-50458-w)
Supplement: Supplementary file 9 — Supplementary Table 4. [file 41598_2023_50458_MOESM9_ESM.docx]

***Table S4:*** *Probability of* ***Small, Medium*** *and* ***Large Body Size*** *survival (include purebred individuals only), per* ***Decimal Year****. Includes the following statistics per decimal year:* $\boldsymbol{N}_{\boldsymbol{A}}$ *i.e., total number of individuals still alive;* $\boldsymbol{N}_{\boldsymbol{D}}$ *i.e., total number of deaths;* ***Survival Probability*** *(%);* ***Standard Error*** *(SE);* ***Lower 95% Confidence Interval*** *and* ***Upper 95% Confidence Interval****. Highlighted rows identify that 95% of small, medium and large individuals are deceased by 18.1, 18.3 and 17.9 years of age, respectively.*

|  | ***Small*** | | | | | | ***Medium*** | | | | | | ***Large*** | | | | | |
| --- | --- | --- | --- | --- | --- | --- | --- | --- | --- | --- | --- | --- | --- | --- | --- | --- | --- | --- |
| ***Decimal Year*** | $\boldsymbol{N}_{\boldsymbol{A}}$ | $\boldsymbol{N}_{\boldsymbol{D}}$ | ***Survival Probability*** | ***SE*** | ***Lower 95% CI*** | ***Upper 95% CI*** | $\boldsymbol{N}_{\boldsymbol{A}}$ | $\boldsymbol{N}_{\boldsymbol{D}}$ | ***Survival Probability*** | ***SE*** | ***Lower 95% CI*** | ***Upper 95% CI*** | $\boldsymbol{N}_{\boldsymbol{A}}$ | $\boldsymbol{N}_{\boldsymbol{D}}$ | ***Survival Probability*** | ***SE*** | ***Lower 95% CI*** | ***Upper 95% CI*** |
| 0 | 222740 | 202 | 0.999 | 0.000 | 0.999 | 0.999 | 73908 | 57 | 0.999 | 0.000 | 0.999 | 0.999 | 123178 | 104 | 0.999 | 0.000 | 0.999 | 0.999 |
| 0.1 | 222370 | 305 | 0.998 | 0.000 | 0.998 | 0.998 | 73794 | 86 | 0.998 | 0.000 | 0.998 | 0.998 | 122959 | 141 | 0.998 | 0.000 | 0.998 | 0.998 |
| 0.2 | 222058 | 1017 | 0.993 | 0.000 | 0.993 | 0.993 | 73705 | 316 | 0.994 | 0.000 | 0.993 | 0.994 | 122815 | 452 | 0.994 | 0.000 | 0.994 | 0.995 |
| 0.3 | 220950 | 734 | 0.990 | 0.000 | 0.989 | 0.990 | 73362 | 283 | 0.990 | 0.000 | 0.989 | 0.991 | 122327 | 335 | 0.992 | 0.000 | 0.991 | 0.992 |
| 0.4 | 220014 | 385 | 0.988 | 0.000 | 0.988 | 0.989 | 73001 | 158 | 0.988 | 0.000 | 0.987 | 0.989 | 121916 | 149 | 0.990 | 0.000 | 0.990 | 0.991 |
| 0.5 | 219279 | 352 | 0.987 | 0.000 | 0.986 | 0.987 | 72732 | 155 | 0.986 | 0.000 | 0.985 | 0.987 | 121604 | 201 | 0.989 | 0.000 | 0.988 | 0.989 |
| 0.6 | 218592 | 335 | 0.985 | 0.000 | 0.985 | 0.986 | 72454 | 108 | 0.984 | 0.000 | 0.983 | 0.985 | 121210 | 172 | 0.987 | 0.000 | 0.987 | 0.988 |
| 0.7 | 217877 | 368 | 0.983 | 0.000 | 0.983 | 0.984 | 72221 | 115 | 0.983 | 0.000 | 0.982 | 0.984 | 120811 | 177 | 0.986 | 0.000 | 0.985 | 0.987 |
| 0.8 | 217087 | 489 | 0.981 | 0.000 | 0.981 | 0.982 | 71946 | 161 | 0.980 | 0.001 | 0.979 | 0.981 | 120425 | 225 | 0.984 | 0.000 | 0.983 | 0.985 |
| 0.9 | 215670 | 323 | 0.980 | 0.000 | 0.979 | 0.980 | 71494 | 112 | 0.979 | 0.001 | 0.978 | 0.980 | 119798 | 158 | 0.983 | 0.000 | 0.982 | 0.984 |
| 1 | 214461 | 452 | 0.978 | 0.000 | 0.977 | 0.978 | 71121 | 153 | 0.977 | 0.001 | 0.976 | 0.978 | 119199 | 206 | 0.981 | 0.000 | 0.980 | 0.982 |
| 1.1 | 213015 | 318 | 0.976 | 0.000 | 0.976 | 0.977 | 70626 | 136 | 0.975 | 0.001 | 0.974 | 0.976 | 118454 | 202 | 0.979 | 0.000 | 0.979 | 0.980 |
| 1.2 | 211714 | 375 | 0.974 | 0.000 | 0.974 | 0.975 | 70126 | 123 | 0.973 | 0.001 | 0.972 | 0.974 | 117741 | 165 | 0.978 | 0.000 | 0.977 | 0.979 |
| 1.3 | 210343 | 510 | 0.972 | 0.000 | 0.971 | 0.973 | 69677 | 190 | 0.971 | 0.001 | 0.969 | 0.972 | 117079 | 250 | 0.976 | 0.000 | 0.975 | 0.977 |
| 1.4 | 208667 | 310 | 0.971 | 0.000 | 0.970 | 0.971 | 69071 | 118 | 0.969 | 0.001 | 0.968 | 0.970 | 116251 | 160 | 0.975 | 0.000 | 0.974 | 0.975 |
| 1.5 | 207327 | 331 | 0.969 | 0.000 | 0.968 | 0.970 | 68605 | 122 | 0.967 | 0.001 | 0.966 | 0.968 | 115596 | 175 | 0.973 | 0.000 | 0.972 | 0.974 |
| 1.6 | 206148 | 281 | 0.968 | 0.000 | 0.967 | 0.968 | 68197 | 122 | 0.965 | 0.001 | 0.964 | 0.967 | 114991 | 164 | 0.972 | 0.000 | 0.971 | 0.973 |
| 1.7 | 204947 | 271 | 0.966 | 0.000 | 0.966 | 0.967 | 67784 | 117 | 0.964 | 0.001 | 0.962 | 0.965 | 114385 | 142 | 0.971 | 0.000 | 0.970 | 0.971 |
| 1.8 | 203747 | 426 | 0.964 | 0.000 | 0.964 | 0.965 | 67391 | 179 | 0.961 | 0.001 | 0.960 | 0.963 | 113830 | 173 | 0.969 | 0.000 | 0.968 | 0.970 |
| 1.9 | 202054 | 265 | 0.963 | 0.000 | 0.962 | 0.964 | 66833 | 92 | 0.960 | 0.001 | 0.958 | 0.961 | 113012 | 149 | 0.968 | 0.001 | 0.967 | 0.969 |
| 2 | 200815 | 429 | 0.961 | 0.000 | 0.960 | 0.962 | 66387 | 167 | 0.958 | 0.001 | 0.956 | 0.959 | 112349 | 229 | 0.966 | 0.001 | 0.965 | 0.967 |
| 2.1 | 199317 | 278 | 0.960 | 0.000 | 0.959 | 0.961 | 65872 | 120 | 0.956 | 0.001 | 0.954 | 0.957 | 111564 | 170 | 0.964 | 0.001 | 0.963 | 0.965 |
| 2.2 | 198053 | 309 | 0.958 | 0.000 | 0.957 | 0.959 | 65437 | 125 | 0.954 | 0.001 | 0.952 | 0.955 | 110917 | 192 | 0.963 | 0.001 | 0.962 | 0.964 |
| 2.3 | 196646 | 434 | 0.956 | 0.000 | 0.955 | 0.957 | 64955 | 171 | 0.951 | 0.001 | 0.950 | 0.953 | 110145 | 228 | 0.961 | 0.001 | 0.960 | 0.962 |
| 2.4 | 194983 | 295 | 0.955 | 0.000 | 0.954 | 0.956 | 64360 | 123 | 0.950 | 0.001 | 0.948 | 0.951 | 109283 | 141 | 0.959 | 0.001 | 0.958 | 0.961 |
| 2.5 | 193717 | 290 | 0.953 | 0.000 | 0.952 | 0.954 | 63896 | 128 | 0.948 | 0.001 | 0.946 | 0.949 | 108646 | 162 | 0.958 | 0.001 | 0.957 | 0.959 |
| 2.6 | 192500 | 292 | 0.952 | 0.000 | 0.951 | 0.953 | 63448 | 95 | 0.946 | 0.001 | 0.945 | 0.948 | 108008 | 140 | 0.957 | 0.001 | 0.956 | 0.958 |
| 2.7 | 191322 | 326 | 0.950 | 0.000 | 0.949 | 0.951 | 63048 | 93 | 0.945 | 0.001 | 0.943 | 0.947 | 107467 | 149 | 0.955 | 0.001 | 0.954 | 0.957 |
| 2.8 | 190089 | 441 | 0.948 | 0.000 | 0.947 | 0.949 | 62662 | 152 | 0.943 | 0.001 | 0.941 | 0.944 | 106909 | 200 | 0.954 | 0.001 | 0.952 | 0.955 |
| 2.9 | 188444 | 295 | 0.947 | 0.000 | 0.946 | 0.947 | 62129 | 82 | 0.941 | 0.001 | 0.940 | 0.943 | 106140 | 131 | 0.952 | 0.001 | 0.951 | 0.954 |
| 3 | 187168 | 542 | 0.944 | 0.001 | 0.943 | 0.945 | 61697 | 188 | 0.938 | 0.001 | 0.937 | 0.940 | 105522 | 225 | 0.950 | 0.001 | 0.949 | 0.952 |
| 3.1 | 185564 | 319 | 0.942 | 0.001 | 0.941 | 0.943 | 61161 | 119 | 0.937 | 0.001 | 0.935 | 0.938 | 104733 | 175 | 0.949 | 0.001 | 0.948 | 0.950 |
| 3.2 | 184303 | 333 | 0.940 | 0.001 | 0.939 | 0.941 | 60698 | 119 | 0.935 | 0.001 | 0.933 | 0.937 | 104082 | 156 | 0.947 | 0.001 | 0.946 | 0.949 |
| 3.3 | 182935 | 476 | 0.938 | 0.001 | 0.937 | 0.939 | 60241 | 131 | 0.933 | 0.001 | 0.931 | 0.935 | 103357 | 235 | 0.945 | 0.001 | 0.944 | 0.947 |
| 3.4 | 181265 | 285 | 0.937 | 0.001 | 0.936 | 0.938 | 59725 | 114 | 0.931 | 0.001 | 0.929 | 0.933 | 102508 | 160 | 0.944 | 0.001 | 0.942 | 0.945 |
| 3.5 | 180004 | 294 | 0.935 | 0.001 | 0.934 | 0.936 | 59327 | 97 | 0.929 | 0.001 | 0.928 | 0.931 | 101887 | 166 | 0.942 | 0.001 | 0.941 | 0.944 |
| 3.6 | 178804 | 303 | 0.933 | 0.001 | 0.932 | 0.935 | 58929 | 90 | 0.928 | 0.001 | 0.926 | 0.930 | 101229 | 139 | 0.941 | 0.001 | 0.940 | 0.942 |
| 3.7 | 177711 | 303 | 0.932 | 0.001 | 0.931 | 0.933 | 58566 | 106 | 0.926 | 0.001 | 0.924 | 0.928 | 100712 | 157 | 0.939 | 0.001 | 0.938 | 0.941 |
| 3.8 | 176584 | 432 | 0.930 | 0.001 | 0.928 | 0.931 | 58228 | 143 | 0.924 | 0.001 | 0.922 | 0.926 | 100148 | 222 | 0.937 | 0.001 | 0.936 | 0.939 |
| 3.9 | 174992 | 297 | 0.928 | 0.001 | 0.927 | 0.929 | 57712 | 97 | 0.923 | 0.001 | 0.921 | 0.925 | 99412 | 138 | 0.936 | 0.001 | 0.935 | 0.938 |
| 4 | 173822 | 516 | 0.925 | 0.001 | 0.924 | 0.926 | 57315 | 216 | 0.919 | 0.001 | 0.917 | 0.921 | 98841 | 261 | 0.934 | 0.001 | 0.932 | 0.935 |
| 4.1 | 172250 | 315 | 0.924 | 0.001 | 0.922 | 0.925 | 56712 | 115 | 0.917 | 0.001 | 0.915 | 0.919 | 98017 | 176 | 0.932 | 0.001 | 0.931 | 0.933 |
| 4.2 | 171073 | 291 | 0.922 | 0.001 | 0.921 | 0.923 | 56297 | 106 | 0.915 | 0.001 | 0.913 | 0.918 | 97428 | 153 | 0.931 | 0.001 | 0.929 | 0.932 |
| 4.3 | 169869 | 436 | 0.920 | 0.001 | 0.918 | 0.921 | 55855 | 160 | 0.913 | 0.001 | 0.911 | 0.915 | 96785 | 271 | 0.928 | 0.001 | 0.926 | 0.929 |
| 4.4 | 168350 | 294 | 0.918 | 0.001 | 0.917 | 0.919 | 55348 | 104 | 0.911 | 0.001 | 0.909 | 0.913 | 95969 | 177 | 0.926 | 0.001 | 0.925 | 0.928 |
| 4.5 | 167254 | 340 | 0.916 | 0.001 | 0.915 | 0.917 | 54966 | 123 | 0.909 | 0.001 | 0.907 | 0.911 | 95360 | 181 | 0.924 | 0.001 | 0.923 | 0.926 |
| 4.6 | 166087 | 298 | 0.914 | 0.001 | 0.913 | 0.916 | 54597 | 107 | 0.907 | 0.001 | 0.905 | 0.910 | 94762 | 163 | 0.923 | 0.001 | 0.921 | 0.924 |
| 4.7 | 165024 | 278 | 0.913 | 0.001 | 0.912 | 0.914 | 54248 | 112 | 0.905 | 0.001 | 0.903 | 0.908 | 94255 | 163 | 0.921 | 0.001 | 0.920 | 0.923 |
| 4.8 | 164056 | 449 | 0.910 | 0.001 | 0.909 | 0.912 | 53902 | 154 | 0.903 | 0.001 | 0.901 | 0.905 | 93716 | 234 | 0.919 | 0.001 | 0.917 | 0.921 |
| 4.9 | 162616 | 321 | 0.909 | 0.001 | 0.907 | 0.910 | 53435 | 115 | 0.901 | 0.001 | 0.899 | 0.903 | 93030 | 169 | 0.917 | 0.001 | 0.916 | 0.919 |
| 5 | 161474 | 645 | 0.905 | 0.001 | 0.904 | 0.906 | 53034 | 220 | 0.897 | 0.001 | 0.895 | 0.900 | 92486 | 319 | 0.914 | 0.001 | 0.912 | 0.916 |
| 5.1 | 159733 | 323 | 0.903 | 0.001 | 0.902 | 0.904 | 52469 | 125 | 0.895 | 0.001 | 0.893 | 0.897 | 91644 | 201 | 0.912 | 0.001 | 0.910 | 0.914 |
| 5.2 | 158709 | 310 | 0.901 | 0.001 | 0.900 | 0.903 | 52122 | 111 | 0.893 | 0.001 | 0.891 | 0.896 | 91085 | 201 | 0.910 | 0.001 | 0.908 | 0.912 |
| 5.3 | 157570 | 459 | 0.899 | 0.001 | 0.897 | 0.900 | 51719 | 173 | 0.890 | 0.001 | 0.888 | 0.893 | 90451 | 280 | 0.907 | 0.001 | 0.906 | 0.909 |
| 5.4 | 156069 | 322 | 0.897 | 0.001 | 0.896 | 0.898 | 51219 | 129 | 0.888 | 0.001 | 0.886 | 0.890 | 89650 | 206 | 0.905 | 0.001 | 0.903 | 0.907 |
| 5.5 | 154949 | 375 | 0.895 | 0.001 | 0.893 | 0.896 | 50819 | 118 | 0.886 | 0.001 | 0.883 | 0.888 | 89008 | 236 | 0.903 | 0.001 | 0.901 | 0.905 |
| 5.6 | 153852 | 327 | 0.893 | 0.001 | 0.892 | 0.894 | 50426 | 121 | 0.884 | 0.001 | 0.881 | 0.886 | 88317 | 196 | 0.901 | 0.001 | 0.899 | 0.903 |
| 5.7 | 152781 | 315 | 0.891 | 0.001 | 0.890 | 0.892 | 50069 | 104 | 0.882 | 0.001 | 0.879 | 0.884 | 87760 | 202 | 0.899 | 0.001 | 0.897 | 0.901 |
| 5.8 | 151844 | 466 | 0.888 | 0.001 | 0.887 | 0.890 | 49717 | 148 | 0.879 | 0.001 | 0.877 | 0.882 | 87213 | 319 | 0.895 | 0.001 | 0.894 | 0.897 |
| 5.9 | 150424 | 343 | 0.886 | 0.001 | 0.885 | 0.888 | 49221 | 109 | 0.877 | 0.001 | 0.875 | 0.880 | 86378 | 222 | 0.893 | 0.001 | 0.891 | 0.895 |
| 6 | 149296 | 586 | 0.883 | 0.001 | 0.881 | 0.884 | 48872 | 213 | 0.874 | 0.001 | 0.871 | 0.876 | 85757 | 328 | 0.890 | 0.001 | 0.888 | 0.892 |
| 6.1 | 147727 | 383 | 0.881 | 0.001 | 0.879 | 0.882 | 48329 | 142 | 0.871 | 0.001 | 0.868 | 0.874 | 84841 | 261 | 0.887 | 0.001 | 0.885 | 0.889 |
| 6.2 | 146536 | 382 | 0.878 | 0.001 | 0.877 | 0.880 | 47881 | 134 | 0.868 | 0.001 | 0.866 | 0.871 | 84123 | 242 | 0.884 | 0.001 | 0.883 | 0.886 |
| 6.3 | 145324 | 528 | 0.875 | 0.001 | 0.874 | 0.877 | 47415 | 196 | 0.865 | 0.001 | 0.862 | 0.868 | 83399 | 383 | 0.880 | 0.001 | 0.878 | 0.882 |
| 6.4 | 143824 | 352 | 0.873 | 0.001 | 0.871 | 0.874 | 46837 | 119 | 0.863 | 0.001 | 0.860 | 0.865 | 82553 | 259 | 0.878 | 0.001 | 0.876 | 0.880 |
| 6.5 | 142681 | 366 | 0.871 | 0.001 | 0.869 | 0.872 | 46465 | 132 | 0.860 | 0.001 | 0.858 | 0.863 | 81835 | 276 | 0.875 | 0.001 | 0.873 | 0.877 |
| 6.6 | 141538 | 382 | 0.868 | 0.001 | 0.867 | 0.870 | 46075 | 132 | 0.858 | 0.001 | 0.855 | 0.861 | 81202 | 239 | 0.872 | 0.001 | 0.870 | 0.874 |
| 6.7 | 140427 | 371 | 0.866 | 0.001 | 0.864 | 0.868 | 45701 | 126 | 0.855 | 0.001 | 0.853 | 0.858 | 80639 | 242 | 0.869 | 0.001 | 0.867 | 0.871 |
| 6.8 | 139405 | 559 | 0.863 | 0.001 | 0.861 | 0.864 | 45332 | 205 | 0.852 | 0.001 | 0.849 | 0.854 | 80040 | 405 | 0.865 | 0.001 | 0.863 | 0.867 |
| 6.9 | 137920 | 351 | 0.860 | 0.001 | 0.859 | 0.862 | 44839 | 146 | 0.849 | 0.001 | 0.846 | 0.852 | 79139 | 281 | 0.862 | 0.001 | 0.860 | 0.864 |
| 7 | 136815 | 689 | 0.856 | 0.001 | 0.854 | 0.858 | 44400 | 235 | 0.844 | 0.001 | 0.841 | 0.847 | 78460 | 476 | 0.857 | 0.001 | 0.855 | 0.859 |
| 7.1 | 135131 | 416 | 0.853 | 0.001 | 0.852 | 0.855 | 43787 | 165 | 0.841 | 0.001 | 0.838 | 0.844 | 77490 | 299 | 0.853 | 0.001 | 0.851 | 0.856 |
| 7.2 | 133981 | 440 | 0.851 | 0.001 | 0.849 | 0.852 | 43353 | 172 | 0.838 | 0.001 | 0.835 | 0.841 | 76758 | 314 | 0.850 | 0.001 | 0.848 | 0.852 |
| 7.3 | 132713 | 656 | 0.846 | 0.001 | 0.845 | 0.848 | 42857 | 201 | 0.834 | 0.002 | 0.831 | 0.837 | 75979 | 479 | 0.845 | 0.001 | 0.842 | 0.847 |
| 7.4 | 131117 | 432 | 0.844 | 0.001 | 0.842 | 0.845 | 42337 | 132 | 0.831 | 0.002 | 0.828 | 0.834 | 74982 | 301 | 0.841 | 0.001 | 0.839 | 0.843 |
| 7.5 | 129860 | 504 | 0.840 | 0.001 | 0.839 | 0.842 | 41932 | 172 | 0.828 | 0.002 | 0.825 | 0.831 | 74190 | 387 | 0.837 | 0.001 | 0.835 | 0.839 |
| 7.6 | 128632 | 410 | 0.838 | 0.001 | 0.836 | 0.839 | 41532 | 137 | 0.825 | 0.002 | 0.822 | 0.828 | 73422 | 331 | 0.833 | 0.001 | 0.831 | 0.835 |
| 7.7 | 127556 | 451 | 0.835 | 0.001 | 0.833 | 0.836 | 41162 | 177 | 0.822 | 0.002 | 0.818 | 0.825 | 72733 | 336 | 0.829 | 0.001 | 0.827 | 0.832 |
| 7.8 | 126392 | 691 | 0.830 | 0.001 | 0.828 | 0.832 | 40740 | 195 | 0.818 | 0.002 | 0.815 | 0.821 | 71990 | 535 | 0.823 | 0.001 | 0.821 | 0.825 |
| 7.9 | 124725 | 439 | 0.827 | 0.001 | 0.825 | 0.829 | 40227 | 165 | 0.814 | 0.002 | 0.811 | 0.817 | 70981 | 370 | 0.819 | 0.001 | 0.816 | 0.821 |
| 8 | 123527 | 895 | 0.821 | 0.001 | 0.819 | 0.823 | 39793 | 337 | 0.807 | 0.002 | 0.804 | 0.811 | 70127 | 637 | 0.811 | 0.001 | 0.809 | 0.814 |
| 8.1 | 121718 | 524 | 0.818 | 0.001 | 0.816 | 0.819 | 39123 | 183 | 0.804 | 0.002 | 0.800 | 0.807 | 68934 | 423 | 0.806 | 0.001 | 0.804 | 0.809 |
| 8.2 | 120427 | 538 | 0.814 | 0.001 | 0.812 | 0.816 | 38693 | 194 | 0.800 | 0.002 | 0.796 | 0.803 | 68093 | 383 | 0.802 | 0.001 | 0.799 | 0.804 |
| 8.3 | 119032 | 784 | 0.809 | 0.001 | 0.807 | 0.810 | 38197 | 253 | 0.794 | 0.002 | 0.791 | 0.798 | 67246 | 556 | 0.795 | 0.001 | 0.793 | 0.798 |
| 8.4 | 117304 | 490 | 0.805 | 0.001 | 0.803 | 0.807 | 37627 | 185 | 0.790 | 0.002 | 0.787 | 0.794 | 66154 | 413 | 0.790 | 0.001 | 0.788 | 0.793 |
| 8.5 | 115958 | 576 | 0.801 | 0.001 | 0.799 | 0.803 | 37155 | 174 | 0.787 | 0.002 | 0.783 | 0.790 | 65251 | 487 | 0.784 | 0.001 | 0.782 | 0.787 |
| 8.6 | 114699 | 563 | 0.797 | 0.001 | 0.795 | 0.799 | 36761 | 187 | 0.783 | 0.002 | 0.779 | 0.786 | 64409 | 413 | 0.779 | 0.001 | 0.777 | 0.782 |
| 8.7 | 113421 | 506 | 0.794 | 0.001 | 0.792 | 0.796 | 36343 | 176 | 0.779 | 0.002 | 0.775 | 0.782 | 63612 | 378 | 0.775 | 0.001 | 0.772 | 0.777 |
| 8.8 | 112239 | 853 | 0.788 | 0.001 | 0.786 | 0.790 | 35900 | 274 | 0.773 | 0.002 | 0.769 | 0.776 | 62834 | 635 | 0.767 | 0.001 | 0.764 | 0.770 |
| 8.9 | 110555 | 525 | 0.784 | 0.001 | 0.782 | 0.786 | 35327 | 179 | 0.769 | 0.002 | 0.766 | 0.773 | 61683 | 449 | 0.761 | 0.001 | 0.759 | 0.764 |
| 9 | 109321 | 988 | 0.777 | 0.001 | 0.775 | 0.779 | 34901 | 334 | 0.762 | 0.002 | 0.758 | 0.765 | 60811 | 729 | 0.752 | 0.001 | 0.749 | 0.755 |
| 9.1 | 107471 | 652 | 0.772 | 0.001 | 0.770 | 0.774 | 34255 | 199 | 0.757 | 0.002 | 0.754 | 0.761 | 59566 | 470 | 0.746 | 0.001 | 0.743 | 0.749 |
| 9.2 | 106174 | 606 | 0.768 | 0.001 | 0.766 | 0.770 | 33801 | 185 | 0.753 | 0.002 | 0.749 | 0.757 | 58678 | 500 | 0.740 | 0.001 | 0.737 | 0.743 |
| 9.3 | 104822 | 911 | 0.761 | 0.001 | 0.759 | 0.763 | 33284 | 302 | 0.746 | 0.002 | 0.743 | 0.750 | 57698 | 746 | 0.730 | 0.001 | 0.727 | 0.733 |
| 9.4 | 103048 | 608 | 0.757 | 0.001 | 0.755 | 0.759 | 32661 | 188 | 0.742 | 0.002 | 0.738 | 0.746 | 56415 | 473 | 0.724 | 0.001 | 0.721 | 0.727 |
| 9.5 | 101698 | 668 | 0.752 | 0.001 | 0.750 | 0.754 | 32203 | 210 | 0.737 | 0.002 | 0.733 | 0.741 | 55471 | 565 | 0.717 | 0.002 | 0.714 | 0.720 |
| 9.6 | 100453 | 633 | 0.747 | 0.001 | 0.745 | 0.749 | 31786 | 203 | 0.732 | 0.002 | 0.729 | 0.736 | 54500 | 438 | 0.711 | 0.002 | 0.708 | 0.714 |
| 9.7 | 99211 | 614 | 0.742 | 0.001 | 0.740 | 0.744 | 31367 | 215 | 0.727 | 0.002 | 0.724 | 0.731 | 53711 | 483 | 0.705 | 0.002 | 0.702 | 0.708 |
| 9.8 | 97935 | 963 | 0.735 | 0.001 | 0.733 | 0.737 | 30941 | 331 | 0.720 | 0.002 | 0.716 | 0.723 | 52879 | 642 | 0.696 | 0.002 | 0.693 | 0.699 |
| 9.9 | 96115 | 619 | 0.730 | 0.001 | 0.728 | 0.732 | 30355 | 197 | 0.715 | 0.002 | 0.711 | 0.719 | 51770 | 477 | 0.690 | 0.002 | 0.687 | 0.693 |
| 10 | 94829 | 1252 | 0.721 | 0.001 | 0.718 | 0.723 | 29950 | 392 | 0.706 | 0.002 | 0.702 | 0.710 | 50874 | 879 | 0.678 | 0.002 | 0.675 | 0.681 |
| 10.1 | 92786 | 751 | 0.715 | 0.001 | 0.713 | 0.717 | 29238 | 245 | 0.700 | 0.002 | 0.696 | 0.704 | 49470 | 545 | 0.670 | 0.002 | 0.667 | 0.673 |
| 10.2 | 91331 | 691 | 0.709 | 0.001 | 0.707 | 0.712 | 28787 | 237 | 0.694 | 0.002 | 0.690 | 0.698 | 48535 | 526 | 0.663 | 0.002 | 0.660 | 0.666 |
| 10.3 | 89921 | 1111 | 0.701 | 0.001 | 0.698 | 0.703 | 28259 | 331 | 0.686 | 0.002 | 0.682 | 0.690 | 47593 | 831 | 0.651 | 0.002 | 0.648 | 0.655 |
| 10.4 | 88003 | 699 | 0.695 | 0.001 | 0.693 | 0.697 | 27637 | 230 | 0.680 | 0.002 | 0.676 | 0.684 | 46287 | 498 | 0.644 | 0.002 | 0.641 | 0.648 |
| 10.5 | 86683 | 829 | 0.688 | 0.001 | 0.686 | 0.691 | 27185 | 279 | 0.673 | 0.002 | 0.669 | 0.677 | 45404 | 646 | 0.635 | 0.002 | 0.632 | 0.638 |
| 10.6 | 85286 | 701 | 0.683 | 0.001 | 0.680 | 0.685 | 26707 | 230 | 0.667 | 0.002 | 0.663 | 0.671 | 44379 | 543 | 0.627 | 0.002 | 0.624 | 0.631 |
| 10.7 | 84032 | 723 | 0.677 | 0.001 | 0.675 | 0.679 | 26310 | 239 | 0.661 | 0.002 | 0.657 | 0.665 | 43510 | 527 | 0.620 | 0.002 | 0.617 | 0.623 |
| 10.8 | 82712 | 1182 | 0.667 | 0.001 | 0.665 | 0.670 | 25867 | 375 | 0.652 | 0.002 | 0.647 | 0.656 | 42657 | 764 | 0.609 | 0.002 | 0.605 | 0.612 |
| 10.9 | 80841 | 739 | 0.661 | 0.001 | 0.659 | 0.664 | 25249 | 240 | 0.645 | 0.002 | 0.641 | 0.650 | 41489 | 551 | 0.601 | 0.002 | 0.597 | 0.604 |
| 11 | 79377 | 1386 | 0.650 | 0.001 | 0.647 | 0.652 | 24780 | 477 | 0.633 | 0.002 | 0.629 | 0.637 | 40551 | 938 | 0.587 | 0.002 | 0.583 | 0.590 |
| 11.1 | 77239 | 814 | 0.643 | 0.001 | 0.640 | 0.645 | 24031 | 286 | 0.625 | 0.002 | 0.621 | 0.630 | 39179 | 602 | 0.578 | 0.002 | 0.574 | 0.581 |
| 11.2 | 75762 | 797 | 0.636 | 0.001 | 0.633 | 0.638 | 23489 | 274 | 0.618 | 0.002 | 0.614 | 0.623 | 38192 | 588 | 0.569 | 0.002 | 0.565 | 0.572 |
| 11.3 | 74384 | 1233 | 0.625 | 0.001 | 0.623 | 0.628 | 22923 | 372 | 0.608 | 0.002 | 0.604 | 0.613 | 37126 | 844 | 0.556 | 0.002 | 0.552 | 0.559 |
| 11.4 | 72333 | 819 | 0.618 | 0.001 | 0.616 | 0.621 | 22242 | 256 | 0.601 | 0.002 | 0.597 | 0.606 | 35785 | 567 | 0.547 | 0.002 | 0.544 | 0.551 |
| 11.5 | 70775 | 900 | 0.610 | 0.001 | 0.608 | 0.613 | 21730 | 293 | 0.593 | 0.002 | 0.589 | 0.598 | 34754 | 672 | 0.537 | 0.002 | 0.533 | 0.540 |
| 11.6 | 69266 | 849 | 0.603 | 0.001 | 0.600 | 0.606 | 21222 | 276 | 0.585 | 0.002 | 0.581 | 0.590 | 33699 | 586 | 0.527 | 0.002 | 0.524 | 0.531 |
| 11.7 | 67842 | 746 | 0.596 | 0.001 | 0.594 | 0.599 | 20751 | 288 | 0.577 | 0.002 | 0.573 | 0.582 | 32762 | 533 | 0.519 | 0.002 | 0.515 | 0.522 |
| 11.8 | 66548 | 1226 | 0.585 | 0.001 | 0.583 | 0.588 | 20302 | 389 | 0.566 | 0.002 | 0.562 | 0.571 | 31912 | 854 | 0.505 | 0.002 | 0.501 | 0.508 |
| 11.9 | 64612 | 810 | 0.578 | 0.001 | 0.575 | 0.581 | 19664 | 274 | 0.558 | 0.002 | 0.554 | 0.563 | 30654 | 559 | 0.496 | 0.002 | 0.492 | 0.499 |
| 12 | 63125 | 1609 | 0.563 | 0.001 | 0.561 | 0.566 | 19157 | 506 | 0.544 | 0.002 | 0.539 | 0.548 | 29672 | 1014 | 0.479 | 0.002 | 0.475 | 0.482 |
| 12.1 | 60844 | 966 | 0.554 | 0.001 | 0.552 | 0.557 | 18408 | 298 | 0.535 | 0.002 | 0.530 | 0.539 | 28245 | 623 | 0.468 | 0.002 | 0.464 | 0.472 |
| 12.2 | 59351 | 893 | 0.546 | 0.001 | 0.543 | 0.549 | 17883 | 280 | 0.526 | 0.002 | 0.522 | 0.531 | 27190 | 605 | 0.458 | 0.002 | 0.454 | 0.461 |
| 12.3 | 57770 | 1427 | 0.533 | 0.001 | 0.530 | 0.535 | 17328 | 423 | 0.514 | 0.002 | 0.509 | 0.518 | 26118 | 821 | 0.443 | 0.002 | 0.440 | 0.447 |
| 12.4 | 55627 | 901 | 0.524 | 0.001 | 0.521 | 0.527 | 16646 | 263 | 0.505 | 0.002 | 0.501 | 0.510 | 24818 | 570 | 0.433 | 0.002 | 0.429 | 0.437 |
| 12.5 | 54103 | 968 | 0.515 | 0.001 | 0.512 | 0.517 | 16106 | 295 | 0.496 | 0.002 | 0.491 | 0.501 | 23763 | 704 | 0.420 | 0.002 | 0.417 | 0.424 |
| 12.6 | 52567 | 922 | 0.505 | 0.001 | 0.503 | 0.508 | 15640 | 306 | 0.486 | 0.002 | 0.482 | 0.491 | 22632 | 589 | 0.409 | 0.002 | 0.406 | 0.413 |
| 12.7 | 51089 | 883 | 0.497 | 0.001 | 0.494 | 0.500 | 15155 | 289 | 0.477 | 0.002 | 0.472 | 0.482 | 21695 | 537 | 0.399 | 0.002 | 0.395 | 0.403 |
| 12.8 | 49690 | 1392 | 0.483 | 0.001 | 0.480 | 0.486 | 14703 | 414 | 0.464 | 0.003 | 0.459 | 0.469 | 20819 | 788 | 0.384 | 0.002 | 0.380 | 0.388 |
| 12.9 | 47662 | 898 | 0.474 | 0.001 | 0.471 | 0.477 | 14087 | 260 | 0.455 | 0.003 | 0.450 | 0.460 | 19599 | 552 | 0.373 | 0.002 | 0.370 | 0.377 |
| 13 | 46176 | 1790 | 0.455 | 0.001 | 0.453 | 0.458 | 13640 | 539 | 0.437 | 0.003 | 0.432 | 0.442 | 18650 | 1028 | 0.353 | 0.002 | 0.349 | 0.356 |
| 13.1 | 43671 | 999 | 0.445 | 0.001 | 0.442 | 0.448 | 12878 | 306 | 0.427 | 0.003 | 0.422 | 0.432 | 17122 | 570 | 0.341 | 0.002 | 0.337 | 0.345 |
| 13.2 | 42109 | 954 | 0.435 | 0.001 | 0.432 | 0.438 | 12347 | 333 | 0.415 | 0.003 | 0.410 | 0.420 | 16169 | 542 | 0.330 | 0.002 | 0.326 | 0.333 |
| 13.3 | 40559 | 1403 | 0.420 | 0.001 | 0.417 | 0.423 | 11784 | 475 | 0.399 | 0.003 | 0.394 | 0.404 | 15163 | 686 | 0.315 | 0.002 | 0.311 | 0.318 |
| 13.4 | 38553 | 922 | 0.410 | 0.001 | 0.407 | 0.413 | 11077 | 289 | 0.388 | 0.003 | 0.383 | 0.393 | 14027 | 528 | 0.303 | 0.002 | 0.299 | 0.306 |
| 13.5 | 37030 | 984 | 0.399 | 0.001 | 0.396 | 0.402 | 10565 | 353 | 0.375 | 0.003 | 0.370 | 0.380 | 12988 | 557 | 0.290 | 0.002 | 0.286 | 0.293 |
| 13.6 | 35535 | 944 | 0.388 | 0.001 | 0.385 | 0.391 | 10026 | 296 | 0.364 | 0.003 | 0.359 | 0.369 | 12037 | 482 | 0.278 | 0.002 | 0.275 | 0.282 |
| 13.7 | 34100 | 898 | 0.378 | 0.001 | 0.375 | 0.381 | 9579 | 261 | 0.354 | 0.003 | 0.349 | 0.359 | 11209 | 375 | 0.269 | 0.002 | 0.265 | 0.273 |
| 13.8 | 32739 | 1266 | 0.363 | 0.001 | 0.361 | 0.366 | 9157 | 397 | 0.339 | 0.003 | 0.334 | 0.344 | 10484 | 584 | 0.254 | 0.002 | 0.250 | 0.258 |
| 13.9 | 31078 | 800 | 0.354 | 0.001 | 0.351 | 0.357 | 8612 | 270 | 0.328 | 0.003 | 0.323 | 0.333 | 9636 | 386 | 0.244 | 0.002 | 0.240 | 0.247 |
| 14 | 30036 | 1712 | 0.334 | 0.001 | 0.331 | 0.337 | 8268 | 553 | 0.306 | 0.003 | 0.301 | 0.311 | 9125 | 729 | 0.224 | 0.002 | 0.221 | 0.228 |
| 14.1 | 28116 | 885 | 0.323 | 0.001 | 0.321 | 0.326 | 7651 | 248 | 0.296 | 0.003 | 0.291 | 0.301 | 8302 | 361 | 0.214 | 0.002 | 0.211 | 0.218 |
| 14.2 | 27098 | 828 | 0.314 | 0.001 | 0.311 | 0.316 | 7368 | 258 | 0.286 | 0.003 | 0.281 | 0.291 | 7867 | 295 | 0.206 | 0.002 | 0.203 | 0.210 |
| 14.3 | 26153 | 1162 | 0.300 | 0.001 | 0.297 | 0.302 | 7069 | 355 | 0.272 | 0.003 | 0.267 | 0.277 | 7498 | 464 | 0.194 | 0.002 | 0.190 | 0.197 |
| 14.4 | 24845 | 708 | 0.291 | 0.001 | 0.288 | 0.294 | 6674 | 242 | 0.262 | 0.003 | 0.257 | 0.267 | 6977 | 285 | 0.186 | 0.002 | 0.182 | 0.189 |
| 14.5 | 24040 | 811 | 0.281 | 0.001 | 0.278 | 0.284 | 6403 | 241 | 0.252 | 0.003 | 0.247 | 0.257 | 6635 | 289 | 0.178 | 0.002 | 0.174 | 0.181 |
| 14.6 | 23126 | 766 | 0.272 | 0.001 | 0.269 | 0.275 | 6124 | 231 | 0.242 | 0.002 | 0.238 | 0.247 | 6303 | 226 | 0.171 | 0.002 | 0.168 | 0.175 |
| 14.7 | 22252 | 679 | 0.264 | 0.001 | 0.261 | 0.266 | 5855 | 197 | 0.234 | 0.002 | 0.229 | 0.239 | 6028 | 223 | 0.165 | 0.002 | 0.162 | 0.168 |
| 14.8 | 21465 | 1077 | 0.250 | 0.001 | 0.248 | 0.253 | 5631 | 292 | 0.222 | 0.002 | 0.217 | 0.227 | 5765 | 295 | 0.157 | 0.002 | 0.153 | 0.160 |
| 14.9 | 20279 | 651 | 0.242 | 0.001 | 0.240 | 0.245 | 5295 | 191 | 0.214 | 0.002 | 0.209 | 0.219 | 5435 | 198 | 0.151 | 0.002 | 0.148 | 0.154 |
| 15 | 19536 | 1511 | 0.224 | 0.001 | 0.221 | 0.226 | 5077 | 382 | 0.198 | 0.002 | 0.193 | 0.203 | 5188 | 400 | 0.139 | 0.002 | 0.136 | 0.143 |
| 15.1 | 17913 | 682 | 0.215 | 0.001 | 0.212 | 0.218 | 4651 | 188 | 0.190 | 0.002 | 0.185 | 0.195 | 4746 | 202 | 0.133 | 0.002 | 0.130 | 0.137 |
| 15.2 | 17163 | 647 | 0.207 | 0.001 | 0.204 | 0.210 | 4432 | 155 | 0.183 | 0.002 | 0.179 | 0.188 | 4501 | 177 | 0.128 | 0.002 | 0.125 | 0.131 |
| 15.3 | 16458 | 918 | 0.195 | 0.001 | 0.193 | 0.198 | 4258 | 254 | 0.172 | 0.002 | 0.168 | 0.177 | 4298 | 220 | 0.121 | 0.002 | 0.118 | 0.125 |
| 15.4 | 15430 | 567 | 0.188 | 0.001 | 0.186 | 0.191 | 3971 | 142 | 0.166 | 0.002 | 0.162 | 0.171 | 4049 | 146 | 0.117 | 0.002 | 0.114 | 0.120 |
| 15.5 | 14798 | 559 | 0.181 | 0.001 | 0.179 | 0.184 | 3806 | 181 | 0.158 | 0.002 | 0.154 | 0.163 | 3870 | 159 | 0.112 | 0.002 | 0.109 | 0.115 |
| 15.6 | 14163 | 538 | 0.174 | 0.001 | 0.172 | 0.177 | 3601 | 146 | 0.152 | 0.002 | 0.148 | 0.156 | 3689 | 132 | 0.108 | 0.002 | 0.105 | 0.111 |
| 15.7 | 13551 | 508 | 0.168 | 0.001 | 0.165 | 0.170 | 3425 | 131 | 0.146 | 0.002 | 0.142 | 0.150 | 3527 | 119 | 0.105 | 0.002 | 0.102 | 0.108 |
| 15.8 | 12990 | 704 | 0.159 | 0.001 | 0.156 | 0.161 | 3275 | 177 | 0.138 | 0.002 | 0.134 | 0.142 | 3378 | 164 | 0.100 | 0.002 | 0.097 | 0.103 |
| 15.9 | 12207 | 482 | 0.152 | 0.001 | 0.150 | 0.155 | 3080 | 115 | 0.133 | 0.002 | 0.129 | 0.137 | 3188 | 102 | 0.096 | 0.002 | 0.094 | 0.099 |
| 16 | 11664 | 1043 | 0.139 | 0.001 | 0.136 | 0.141 | 2949 | 230 | 0.123 | 0.002 | 0.119 | 0.127 | 3063 | 229 | 0.089 | 0.001 | 0.086 | 0.092 |
| 16.1 | 10541 | 456 | 0.133 | 0.001 | 0.131 | 0.135 | 2699 | 135 | 0.117 | 0.002 | 0.113 | 0.121 | 2798 | 122 | 0.085 | 0.001 | 0.083 | 0.088 |
| 16.2 | 10040 | 422 | 0.127 | 0.001 | 0.125 | 0.129 | 2535 | 96 | 0.112 | 0.002 | 0.108 | 0.116 | 2654 | 82 | 0.083 | 0.001 | 0.080 | 0.085 |
| 16.3 | 9581 | 540 | 0.120 | 0.001 | 0.118 | 0.122 | 2426 | 127 | 0.106 | 0.002 | 0.102 | 0.110 | 2554 | 91 | 0.080 | 0.001 | 0.077 | 0.082 |
| 16.4 | 8980 | 325 | 0.116 | 0.001 | 0.114 | 0.118 | 2285 | 94 | 0.102 | 0.002 | 0.098 | 0.106 | 2433 | 74 | 0.077 | 0.001 | 0.075 | 0.080 |
| 16.5 | 8603 | 348 | 0.111 | 0.001 | 0.109 | 0.113 | 2180 | 103 | 0.097 | 0.002 | 0.093 | 0.101 | 2330 | 73 | 0.075 | 0.001 | 0.072 | 0.078 |
| 16.6 | 8211 | 343 | 0.106 | 0.001 | 0.104 | 0.108 | 2055 | 83 | 0.093 | 0.002 | 0.090 | 0.097 | 2233 | 67 | 0.073 | 0.001 | 0.070 | 0.075 |
| 16.7 | 7827 | 323 | 0.102 | 0.001 | 0.100 | 0.104 | 1957 | 51 | 0.091 | 0.002 | 0.087 | 0.094 | 2149 | 65 | 0.070 | 0.001 | 0.068 | 0.073 |
| 16.8 | 7451 | 415 | 0.096 | 0.001 | 0.094 | 0.098 | 1893 | 82 | 0.087 | 0.002 | 0.083 | 0.090 | 2073 | 66 | 0.068 | 0.001 | 0.066 | 0.071 |
| 16.9 | 6991 | 234 | 0.093 | 0.001 | 0.091 | 0.095 | 1794 | 62 | 0.084 | 0.002 | 0.080 | 0.087 | 1979 | 42 | 0.067 | 0.001 | 0.064 | 0.069 |
| 17 | 6707 | 623 | 0.084 | 0.001 | 0.083 | 0.086 | 1721 | 118 | 0.078 | 0.002 | 0.075 | 0.082 | 1922 | 99 | 0.063 | 0.001 | 0.061 | 0.066 |
| 17.1 | 6031 | 254 | 0.081 | 0.001 | 0.079 | 0.083 | 1591 | 60 | 0.075 | 0.002 | 0.072 | 0.079 | 1801 | 51 | 0.061 | 0.001 | 0.059 | 0.064 |
| 17.2 | 5742 | 249 | 0.077 | 0.001 | 0.076 | 0.079 | 1513 | 41 | 0.073 | 0.002 | 0.070 | 0.076 | 1737 | 46 | 0.060 | 0.001 | 0.057 | 0.062 |
| 17.3 | 5465 | 318 | 0.073 | 0.001 | 0.071 | 0.075 | 1467 | 59 | 0.070 | 0.002 | 0.067 | 0.073 | 1677 | 51 | 0.058 | 0.001 | 0.056 | 0.061 |
| 17.4 | 5100 | 193 | 0.070 | 0.001 | 0.068 | 0.072 | 1392 | 39 | 0.068 | 0.002 | 0.065 | 0.071 | 1606 | 39 | 0.057 | 0.001 | 0.054 | 0.059 |
| 17.5 | 4879 | 202 | 0.067 | 0.001 | 0.065 | 0.069 | 1342 | 36 | 0.066 | 0.002 | 0.063 | 0.070 | 1554 | 46 | 0.055 | 0.001 | 0.053 | 0.057 |
| 17.6 | 4655 | 197 | 0.064 | 0.001 | 0.063 | 0.066 | 1298 | 39 | 0.064 | 0.002 | 0.061 | 0.068 | 1490 | 34 | 0.054 | 0.001 | 0.051 | 0.056 |
| 17.7 | 4428 | 168 | 0.062 | 0.001 | 0.060 | 0.064 | 1248 | 41 | 0.062 | 0.002 | 0.059 | 0.065 | 1443 | 33 | 0.052 | 0.001 | 0.050 | 0.055 |
| 17.8 | 4228 | 221 | 0.059 | 0.001 | 0.057 | 0.060 | 1201 | 45 | 0.060 | 0.002 | 0.057 | 0.063 | 1394 | 36 | 0.051 | 0.001 | 0.049 | 0.054 |
| 17.9 | 3961 | 138 | 0.057 | 0.001 | 0.055 | 0.058 | 1139 | 34 | 0.058 | 0.002 | 0.055 | 0.061 | 1339 | 39 | 0.050 | 0.001 | 0.047 | 0.052 |
| 18 | 3802 | 375 | 0.051 | 0.001 | 0.049 | 0.053 | 1091 | 52 | 0.055 | 0.002 | 0.052 | 0.058 | 1292 | 54 | 0.048 | 0.001 | 0.045 | 0.050 |
| 18.1 | 3407 | 159 | 0.049 | 0.001 | 0.047 | 0.050 | 1026 | 37 | 0.053 | 0.002 | 0.050 | 0.056 | 1227 | 37 | 0.046 | 0.001 | 0.044 | 0.048 |
| 18.2 | 3226 | 130 | 0.047 | 0.001 | 0.045 | 0.048 | 979 | 33 | 0.052 | 0.001 | 0.049 | 0.055 | 1180 | 34 | 0.045 | 0.001 | 0.043 | 0.047 |
| 18.3 | 3077 | 170 | 0.044 | 0.001 | 0.043 | 0.046 | 939 | 38 | 0.049 | 0.001 | 0.047 | 0.052 | 1137 | 33 | 0.043 | 0.001 | 0.041 | 0.046 |
| 18.4 | 2873 | 117 | 0.042 | 0.001 | 0.041 | 0.044 | 897 | 32 | 0.048 | 0.001 | 0.045 | 0.051 | 1091 | 40 | 0.042 | 0.001 | 0.040 | 0.044 |
| 18.5 | 2733 | 117 | 0.041 | 0.001 | 0.039 | 0.042 | 861 | 25 | 0.046 | 0.001 | 0.044 | 0.049 | 1037 | 35 | 0.040 | 0.001 | 0.038 | 0.043 |
| 18.6 | 2593 | 114 | 0.039 | 0.001 | 0.037 | 0.040 | 828 | 22 | 0.045 | 0.001 | 0.042 | 0.048 | 987 | 26 | 0.039 | 0.001 | 0.037 | 0.042 |
| 18.7 | 2456 | 95 | 0.037 | 0.001 | 0.036 | 0.039 | 796 | 20 | 0.044 | 0.001 | 0.041 | 0.047 | 957 | 24 | 0.038 | 0.001 | 0.036 | 0.041 |
| 18.8 | 2345 | 111 | 0.035 | 0.001 | 0.034 | 0.037 | 772 | 23 | 0.043 | 0.001 | 0.040 | 0.045 | 921 | 27 | 0.037 | 0.001 | 0.035 | 0.039 |
| 18.9 | 2211 | 103 | 0.034 | 0.001 | 0.033 | 0.035 | 740 | 39 | 0.040 | 0.001 | 0.038 | 0.043 | 881 | 28 | 0.036 | 0.001 | 0.034 | 0.038 |
| 19 | 2094 | 148 | 0.031 | 0.001 | 0.030 | 0.033 | 695 | 27 | 0.039 | 0.001 | 0.036 | 0.042 | 847 | 21 | 0.035 | 0.001 | 0.033 | 0.037 |
| 19.1 | 1917 | 80 | 0.030 | 0.001 | 0.029 | 0.031 | 652 | 25 | 0.037 | 0.001 | 0.035 | 0.040 | 815 | 32 | 0.034 | 0.001 | 0.032 | 0.036 |
| 19.2 | 1821 | 54 | 0.029 | 0.001 | 0.028 | 0.031 | 622 | 16 | 0.036 | 0.001 | 0.034 | 0.039 | 777 | 24 | 0.033 | 0.001 | 0.031 | 0.035 |
| 19.3 | 1757 | 72 | 0.028 | 0.001 | 0.027 | 0.029 | 601 | 15 | 0.035 | 0.001 | 0.033 | 0.038 | 747 | 23 | 0.032 | 0.001 | 0.030 | 0.034 |
| 19.4 | 1666 | 65 | 0.027 | 0.001 | 0.026 | 0.028 | 583 | 19 | 0.034 | 0.001 | 0.032 | 0.037 | 714 | 18 | 0.031 | 0.001 | 0.029 | 0.033 |
| 19.5 | 1577 | 60 | 0.026 | 0.001 | 0.025 | 0.027 | 559 | 10 | 0.034 | 0.001 | 0.031 | 0.036 | 693 | 20 | 0.030 | 0.001 | 0.028 | 0.032 |
| 19.6 | 1506 | 69 | 0.025 | 0.001 | 0.024 | 0.026 | 543 | 18 | 0.033 | 0.001 | 0.030 | 0.035 | 671 | 17 | 0.029 | 0.001 | 0.027 | 0.031 |
| 19.7 | 1427 | 36 | 0.024 | 0.001 | 0.023 | 0.025 | 513 | 12 | 0.032 | 0.001 | 0.029 | 0.034 | 649 | 15 | 0.029 | 0.001 | 0.027 | 0.031 |
| 19.8 | 1377 | 56 | 0.023 | 0.001 | 0.022 | 0.024 | 497 | 18 | 0.031 | 0.001 | 0.028 | 0.033 | 627 | 16 | 0.028 | 0.001 | 0.026 | 0.030 |
| 19.9 | 1308 | 48 | 0.022 | 0.001 | 0.021 | 0.023 | 477 | 11 | 0.030 | 0.001 | 0.028 | 0.032 | 608 | 11 | 0.027 | 0.001 | 0.026 | 0.029 |
| 20 | 1255 | 88 | 0.021 | 0.001 | 0.020 | 0.022 | 462 | 10 | 0.029 | 0.001 | 0.027 | 0.032 | 587 | 20 | 0.026 | 0.001 | 0.025 | 0.028 |
| 20.1 | 1152 | 42 | 0.020 | 0.001 | 0.019 | 0.021 | 444 | 16 | 0.028 | 0.001 | 0.026 | 0.031 | 557 | 15 | 0.026 | 0.001 | 0.024 | 0.028 |
| 20.2 | 1103 | 41 | 0.019 | 0.001 | 0.018 | 0.020 | 426 | 13 | 0.027 | 0.001 | 0.025 | 0.030 | 539 | 15 | 0.025 | 0.001 | 0.023 | 0.027 |
| 20.3 | 1054 | 47 | 0.018 | 0.001 | 0.017 | 0.019 | 411 | 8 | 0.027 | 0.001 | 0.025 | 0.029 | 518 | 15 | 0.024 | 0.001 | 0.023 | 0.026 |
| 20.4 | 991 | 34 | 0.018 | 0.001 | 0.017 | 0.019 | 396 | 14 | 0.026 | 0.001 | 0.024 | 0.028 | 500 | 13 | 0.024 | 0.001 | 0.022 | 0.026 |
| 20.5 | 952 | 29 | 0.017 | 0.001 | 0.016 | 0.018 | 380 | 8 | 0.025 | 0.001 | 0.023 | 0.028 | 484 | 16 | 0.023 | 0.001 | 0.021 | 0.025 |
| 20.6 | 918 | 41 | 0.016 | 0.001 | 0.015 | 0.017 | 370 | 24 | 0.024 | 0.001 | 0.022 | 0.026 | 464 | 17 | 0.022 | 0.001 | 0.020 | 0.024 |
| 20.7 | 872 | 20 | 0.016 | 0.001 | 0.015 | 0.017 | 345 | 13 | 0.023 | 0.001 | 0.021 | 0.025 | 444 | 13 | 0.021 | 0.001 | 0.020 | 0.023 |
| 20.8 | 847 | 42 | 0.015 | 0.000 | 0.014 | 0.016 | 328 | 8 | 0.022 | 0.001 | 0.020 | 0.025 | 430 | 14 | 0.021 | 0.001 | 0.019 | 0.023 |
| 20.9 | 798 | 26 | 0.015 | 0.000 | 0.014 | 0.016 | 319 | 8 | 0.022 | 0.001 | 0.020 | 0.024 | 414 | 14 | 0.020 | 0.001 | 0.018 | 0.022 |
| 21 | 762 | 49 | 0.014 | 0.000 | 0.013 | 0.015 | 309 | 12 | 0.021 | 0.001 | 0.019 | 0.023 | 396 | 12 | 0.019 | 0.001 | 0.018 | 0.021 |
| 21.1 | 703 | 40 | 0.013 | 0.000 | 0.012 | 0.014 | 291 | 10 | 0.020 | 0.001 | 0.018 | 0.022 | 377 | 19 | 0.018 | 0.001 | 0.017 | 0.020 |
| 21.2 | 656 | 22 | 0.013 | 0.000 | 0.012 | 0.014 | 278 | 7 | 0.020 | 0.001 | 0.018 | 0.022 | 357 | 15 | 0.018 | 0.001 | 0.016 | 0.019 |
| 21.3 | 626 | 29 | 0.012 | 0.000 | 0.011 | 0.013 | 271 | 9 | 0.019 | 0.001 | 0.017 | 0.021 | 341 | 10 | 0.017 | 0.001 | 0.016 | 0.019 |
| 21.4 | 592 | 22 | 0.012 | 0.000 | 0.011 | 0.012 | 260 | 12 | 0.018 | 0.001 | 0.016 | 0.020 | 329 | 13 | 0.016 | 0.001 | 0.015 | 0.018 |
| 21.5 | 567 | 19 | 0.011 | 0.000 | 0.010 | 0.012 | 246 | 5 | 0.018 | 0.001 | 0.016 | 0.020 | 315 | 10 | 0.016 | 0.001 | 0.014 | 0.018 |
| 21.6 | 544 | 29 | 0.011 | 0.000 | 0.010 | 0.011 | 238 | 8 | 0.017 | 0.001 | 0.015 | 0.019 | 302 | 14 | 0.015 | 0.001 | 0.014 | 0.017 |
| 21.7 | 508 | 25 | 0.010 | 0.000 | 0.009 | 0.011 | 229 | 11 | 0.016 | 0.001 | 0.015 | 0.018 | 287 | 10 | 0.015 | 0.001 | 0.013 | 0.016 |
| 21.8 | 481 | 11 | 0.010 | 0.000 | 0.009 | 0.011 | 215 | 11 | 0.015 | 0.001 | 0.014 | 0.018 | 271 | 12 | 0.014 | 0.001 | 0.013 | 0.016 |
| 21.9 | 464 | 28 | 0.009 | 0.000 | 0.008 | 0.010 | 202 | 10 | 0.015 | 0.001 | 0.013 | 0.017 | 254 | 13 | 0.013 | 0.001 | 0.012 | 0.015 |
| 22 | 429 | 30 | 0.009 | 0.000 | 0.008 | 0.009 | 190 | 9 | 0.014 | 0.001 | 0.012 | 0.016 | 240 | 11 | 0.013 | 0.001 | 0.011 | 0.014 |
| 22.1 | 396 | 35 | 0.008 | 0.000 | 0.007 | 0.009 | 178 | 16 | 0.013 | 0.001 | 0.011 | 0.015 | 228 | 22 | 0.011 | 0.001 | 0.010 | 0.013 |
| 22.2 | 356 | 12 | 0.008 | 0.000 | 0.007 | 0.008 | 160 | 8 | 0.012 | 0.001 | 0.011 | 0.014 | 204 | 6 | 0.011 | 0.001 | 0.010 | 0.013 |
| 22.3 | 341 | 17 | 0.007 | 0.000 | 0.007 | 0.008 | 152 | 6 | 0.012 | 0.001 | 0.010 | 0.013 | 198 | 7 | 0.011 | 0.001 | 0.009 | 0.012 |
| 22.4 | 323 | 18 | 0.007 | 0.000 | 0.006 | 0.008 | 145 | 5 | 0.011 | 0.001 | 0.010 | 0.013 | 189 | 12 | 0.010 | 0.001 | 0.009 | 0.011 |
| 22.5 | 299 | 12 | 0.007 | 0.000 | 0.006 | 0.007 | 139 | 9 | 0.011 | 0.001 | 0.009 | 0.012 | 173 | 13 | 0.009 | 0.001 | 0.008 | 0.011 |
| 22.6 | 286 | 22 | 0.006 | 0.000 | 0.005 | 0.007 | 128 | 10 | 0.010 | 0.001 | 0.008 | 0.011 | 160 | 10 | 0.009 | 0.001 | 0.008 | 0.010 |
| 22.7 | 262 | 16 | 0.006 | 0.000 | 0.005 | 0.006 | 117 | 13 | 0.009 | 0.001 | 0.007 | 0.010 | 148 | 9 | 0.008 | 0.001 | 0.007 | 0.010 |
| 22.8 | 243 | 13 | 0.005 | 0.000 | 0.005 | 0.006 | 103 | 7 | 0.008 | 0.001 | 0.007 | 0.010 | 138 | 9 | 0.008 | 0.001 | 0.007 | 0.009 |
| 22.9 | 225 | 20 | 0.005 | 0.000 | 0.004 | 0.006 | 95 | 10 | 0.007 | 0.001 | 0.006 | 0.009 | 127 | 7 | 0.007 | 0.001 | 0.006 | 0.009 |
| 23 | 204 | 22 | 0.004 | 0.000 | 0.004 | 0.005 | 81 | 4 | 0.007 | 0.001 | 0.006 | 0.008 | 119 | 11 | 0.007 | 0.001 | 0.006 | 0.008 |
| 23.1 | 173 | 21 | 0.004 | 0.000 | 0.003 | 0.004 | 75 | 12 | 0.006 | 0.001 | 0.005 | 0.007 | 105 | 18 | 0.005 | 0.001 | 0.004 | 0.007 |
| 23.2 | 149 | 13 | 0.003 | 0.000 | 0.003 | 0.004 | 60 | 4 | 0.005 | 0.001 | 0.004 | 0.007 | 85 | 6 | 0.005 | 0.001 | 0.004 | 0.006 |
| 23.3 | 134 | 14 | 0.003 | 0.000 | 0.003 | 0.004 | 56 | 6 | 0.005 | 0.001 | 0.004 | 0.006 | 79 | 11 | 0.004 | 0.000 | 0.003 | 0.005 |
| 23.4 | 118 | 16 | 0.003 | 0.000 | 0.002 | 0.003 | 49 | 4 | 0.004 | 0.001 | 0.003 | 0.006 | 68 | 4 | 0.004 | 0.000 | 0.003 | 0.005 |
| 23.5 | 97 | 11 | 0.002 | 0.000 | 0.002 | 0.003 | 42 | 4 | 0.004 | 0.001 | 0.003 | 0.005 | 63 | 8 | 0.004 | 0.000 | 0.003 | 0.005 |
| 23.6 | 85 | 18 | 0.002 | 0.000 | 0.002 | 0.002 | 37 | 3 | 0.004 | 0.001 | 0.003 | 0.005 | 52 | 11 | 0.003 | 0.000 | 0.002 | 0.004 |
| 23.7 | 66 | 16 | 0.001 | 0.000 | 0.001 | 0.002 | 32 | 8 | 0.003 | 0.001 | 0.002 | 0.004 | 41 | 7 | 0.002 | 0.000 | 0.002 | 0.003 |
| 23.8 | 49 | 12 | 0.001 | 0.000 | 0.001 | 0.001 | 23 | 3 | 0.002 | 0.000 | 0.002 | 0.004 | 32 | 9 | 0.002 | 0.000 | 0.001 | 0.002 |
| 23.9 | 35 | 14 | 0.001 | 0.000 | 0.000 | 0.001 | 17 | 6 | 0.002 | 0.000 | 0.001 | 0.003 | 23 | 7 | 0.001 | 0.000 | 0.001 | 0.002 |
| 24 | 19 | 15 | 0.000 | 0.000 | 0.000 | 0.000 | 9 | 6 | 0.001 | 0.000 | 0.000 | 0.001 | 15 | 12 | 0.000 | 0.000 | 0.000 | 0.001 |
