# Supplementary material for: Longevity of companion dog breeds: those at risk from early death
Source: Sci Rep. 2024 Feb 1;14:531. doi: 10.1038/s41598-023-50458-w (PMC10834484; doi:10.1038/s41598-023-50458-w)
Supplement: Supplementary file 10 — Supplementary Table 5. [file 41598_2023_50458_MOESM10_ESM.docx]

***Table S5:*** *Individual breeds* ***Included in Survival Analysis*** *or not (Y/N; due to presence in data), along with their* ***Phylogeny Reclassification*** *(i.e.,* *KC and/or FCI breeds included in ‘Breed’) and associated* ***Phylogeny Abbreviation*** *(those listed as NA are not included in phylogenetic analysis)*. *Reclassification of breeds carried out to match existing canine phylogenies^85,86^ based on alternative breed names and/or ancestry. Breed grouped by* ***KC Group*** *(Kennel Club grouping: Toy; Hound; Pastoral; Terrier; Gundog^83^),* ***FCI Group*** *(Fédération Cynologique Internationale grouping: Sheepdogs and Cattledogs (except Swiss Cattledogs); Terriers; Spitz and primitive types; Pointing Dogs; Companion and Toy Dogs; Pinscher and Schnauzer – Molossoid and Swiss Mountain and Cattledogs; Dachshunds; Scent hounds and related breeds; Retrievers – Flushing Dogs – Water Dogs; Sighthounds^84^),* ***Body Size*** *(Small; Medium; Large), and* ***Cephalic Index*** *(Brachycephalic; Mesocephalic; Dolichocephalic).* KC and FCI Group listed to signify presence of breed in nomenclature only - these grouping variables were not included in analyses.

| ***Breed*** | ***Included in Survival Analysis*** | ***Phylogeny Reclassification*** | ***Phylogeny Abbreviation*** | ***KC Group*** | ***FCI Group*** | ***Body Size*** | ***Cephalic Index*** |
| --- | --- | --- | --- | --- | --- | --- | --- |
| Affenpinscher | Y | NA | NA | Toy | Pinscher and Schnauzer - Molossoid and Swiss Mountain and Cattledogs | Small | Brachycephalic |
| Afghan Hound | Y | NA | AFGH | Hound | Sighthounds | Large | Dolichocephalic |
| Airedale Terrier | Y | NA | AIRT | Terrier | Terriers | Medium | Dolichocephalic |
| Akita | Y | American Akita  Japanese Akita Inu | AKIT | Utility | Spitz and primitive types | Large | Mesocephalic |
| Alaskan Malamute | Y | NA | AMAL | Working | Spitz and primitive types | Large | Mesocephalic |
| American Cocker Spaniel | Y | NA | ACOS | Gundog | Retrievers - Flushing Dogs - Water Dogs | Small | Mesocephalic |
| American Eskimo Dog | Y | Canadian Eskimo Dog | AESK | Working | Spitz and primitive types | Medium | Mesocephalic |
| American Staffordshire Terrier | Y | NA | AMST | NA | Terriers | Medium | Mesocephalic |
| Anatolian Shepherd | Y | NA | ANAT | Pastoral | Pinscher and Schnauzer - Molossoid and Swiss Mountain and Cattledogs | Large | Mesocephalic |
| Australian Cattle Dog | Y | NA | AUCD | Pastoral | Sheepdogs and cattledogs (except Swiss Cattledogs) | Medium | Mesocephalic |
| Australian Shepherd | Y | NA | AUSS | Pastoral | Sheepdogs and cattledogs (except Swiss Cattledogs) | Medium | Mesocephalic |
| Basenji | Y | NA | BSJI | Hound | Spitz and primitive types | Small | Mesocephalic |
| Basset Hound | Y | NA | BASS | Hound | Scent hounds and related breeds | Medium | Dolichocephalic |
| Beagle | Y | NA | BEAG | Hound | Scent hounds and related breeds | Small | Mesocephalic |
| Bearded Collie | Y | NA | BERD | Pastoral | Sheepdogs and cattledogs (except Swiss Cattledogs) | Medium | Mesocephalic |
| Bedlington Terrier | Y | NA | BEDT | Terrier | Terriers | Small | Dolichocephalic |
| Belgian Malenois | Y | NA | BMAL | Pastoral | Sheepdogs and cattledogs (except Swiss Cattledogs) | Medium | Dolichocephalic |
| Belgian Tervuren | Y | Belgian Shepherd Dog (Groenendael) | TURV | Pastoral | Sheepdogs and cattledogs (except Swiss Cattledogs) | Medium | Dolichocephalic |
| Bernese Mountain Dog | Y | NA | BMD | Working | Pinscher and Schnauzer - Molossoid and Swiss Mountain and Cattledogs | Large | Mesocephalic |
| Bichon Frise | Y | NA | BICH | Toy | Companion and Toy Dogs | Small | Mesocephalic |
| Black Russian Terrier | Y | NA | BRTR | Working | Pinscher and Schnauzer - Molossoid and Swiss Mountain and Cattledogs | Large | Mesocephalic |
| Bloodhound | Y | NA | BLDH | Hound | Scent hounds and related breeds | Large | Dolichocephalic |
| Bolognese | Y | NA | BOLG | Toy | Companion and Toy Dogs | Small | Mesocephalic |
| Border Collie | Y | NA | BORD | Pastoral | Sheepdogs and cattledogs (except Swiss Cattledogs) | Medium | Mesocephalic |
| Border Terrier | Y | NA | BORT | Terrier | Terriers | Small | Mesocephalic |
| Borzoi | Y | NA | BORZ | Hound | Sighthounds | Large | Dolichocephalic |
| Boston Terrier | Y | NA | BOST | Utility | Companion and Toy Dogs | Small | Brachycephalic |
| Bouvier des Flandres | Y | NA | BOUV | Working | Sheepdogs and cattledogs (except Swiss Cattledogs) | Large | Mesocephalic |
| Boxer | Y | NA | BOX | Working | Pinscher and Schnauzer - Molossoid and Swiss Mountain and Cattledogs | Large | Brachycephalic |
| Bracco Italiano | Y | NA | BRAC | Gundog | Pointing Dogs | Large | Dolichocephalic |
| Briard | Y | NA | BRIA | Pastoral | Sheepdogs and cattledogs (except Swiss Cattledogs) | Large | Mesocephalic |
| Brittany | Y | NA | BRIT | Gundog | Pointing Dogs | Medium | Mesocephalic |
| Brussels Griffon | Y | Griffon Bruxellois | BRUS | Toy | Companion and Toy Dogs | Small | Brachycephalic |
| Bull Terrier | Y | NA | BULT | Terrier | Terriers | Medium | Dolichocephalic |
| Bulldog | Y | NA | BULD | Utility | Pinscher and Schnauzer - Molossoid and Swiss Mountain and Cattledogs | Medium | Brachycephalic |
| Bullmastiff | Y | NA | BULM | Working | Pinscher and Schnauzer - Molossoid and Swiss Mountain and Cattledogs | Large | Brachycephalic |
| Cairn Terrier | Y | NA | CAIR | Terrier | Terriers | Small | Mesocephalic |
| Canaan Dog | Y | NA | NA | Utility | Spitz and primitive types | Medium | Mesocephalic |
| Cane Corso | Y | NA | CANE | NA | Pinscher and Schnauzer - Molossoid and Swiss Mountain and Cattledogs | Large | Brachycephalic |
| Cardigan Welsh Corgi | Y | NA | CARD | Pastoral | Sheepdogs and cattledogs (except Swiss Cattledogs) | Small | Mesocephalic |
| Caucasian Shepherd Dog | Y | NA | CAUC | NA | Pinscher and Schnauzer - Molossoid and Swiss Mountain and Cattledogs | Large | Mesocephalic |
| Cavalier King Charles Spaniel | Y | King Charles Spaniel | CKCS | Toy | Companion and Toy Dogs | Small | Brachycephalic |
| Chesapeake Bay Retriever | Y | NA | CBR | Gundog | Retrievers - Flushing Dogs - Water Dogs | Large | Mesocephalic |
| Chihuahua | Y | Chihuahua (Long haired)  Chihuahua (Smooth haired) | CHIH | Toy | Companion and Toy Dogs | Small | Brachycephalic |
| Chinese Crested | Y | Powder Puff or Hairless | CRES | Toy | Companion and Toy Dogs | Small | Mesocephalic |
| Chinese Shar Pei | Y | NA | SHAR | Utility | Pinscher and Schnauzer - Molossoid and Swiss Mountain and Cattledogs | Medium | Mesocephalic |
| Chow Chow | Y | NA | CHOW | Utility | Spitz and primitive types | Large | Mesocephalic |
| Clumber Spaniel | Y | NA | CLUS | Gundog | Retrievers - Flushing Dogs - Water Dogs | Large | Mesocephalic |
| Collie | Y | Collie (Rough)  Collie (Smooth) | COLL | Pastoral | Sheepdogs and cattledogs (except Swiss Cattledogs) | Medium | Dolichocephalic |
| Coton de Tulear | Y | NA | COTO | Toy | Companion and Toy Dogs | Small | Mesocephalic |
| Curly Coated Retriever | Y | NA | CCRT | Gundog | Retrievers - Flushing Dogs - Water Dogs | Large | Mesocephalic |
| Dachshund | Y | Dachshund (Long-Haired)  Dachshund (Smooth-Haired)  Dachshund (Wire-Haired) | DACH | Hound | Dachshunds | Medium | Dolichocephalic |
| Dalmatian | Y | NA | DALM | Utility | Scent hounds and related breeds | Medium | Mesocephalic |
| Dandie Dinmont Terrier | Y | NA | NA | Terrier | Terriers | Small | Mesocephalic |
| Doberman Pinscher | Y | Doberman  German Pinscher | DOBP | Working | Pinscher and Schnauzer - Molossoid and Swiss Mountain and Cattledogs | Medium | Dolichocephalic |
| Dogue de Bordeaux | Y | NA | DDBX | Working | Pinscher and Schnauzer - Molossoid and Swiss Mountain and Cattledogs | Large | Brachycephalic |
| English Cocker Spaniel | Y | NA | ECKR | Gundog | Retrievers - Flushing Dogs - Water Dogs | Small | Mesocephalic |
| English Setter | Y | NA | ESET | Gundog | Pointing Dogs | Large | Mesocephalic |
| English Springer Spaniel | Y | NA | ESSP | Gundog | Retrievers - Flushing Dogs - Water Dogs | Medium | Mesocephalic |
| Field Spaniel | Y | NA | FIEL | Gundog | Retrievers - Flushing Dogs - Water Dogs | Medium | Mesocephalic |
| Finnish Lapphund | Y | NA | NA | Pastoral | Spitz and primitive types | Medium | Mesocephalic |
| Flat Coated Retriever | Y | NA | FCR | Gundog | Retrievers - Flushing Dogs - Water Dogs | Large | Mesocephalic |
| Foxhound | Y | NA | FOXH | Hound | Scent hounds and related breeds | Large | Mesocephalic |
| French Bulldog | Y | NA | FBUL | Utility | Companion and Toy Dogs | Small | Brachycephalic |
| German Shepherd Dog | Y | NA | GSD | Pastoral | Sheepdogs and cattledogs (except Swiss Cattledogs) | Large | Dolichocephalic |
| German Shorthaired Pointer | Y | Pointer | GSHP | Gundog | Pointing Dogs | Medium | Mesocephalic |
| German Spitz Mittel | Y | NA | GSPM | Utility | Spitz and primitive types | Medium | Mesocephalic |
| German Wirehaired Pointer | Y | German Longhaired Pointer  German Roughhaired Pointer | GWHP | Gundog | Pointing Dogs | Large | Mesocephalic |
| Giant Schnauzer | Y | NA | GSNZ | Working | Pinscher and Schnauzer - Molossoid and Swiss Mountain and Cattledogs | Large | Mesocephalic |
| Glen of Imaal Terrier | Y | NA | GLEN | Terrier | Terriers | Small | Mesocephalic |
| Golden Retriever | Y | NA | GOLD | Gundog | Retrievers - Flushing Dogs - Water Dogs | Large | Mesocephalic |
| Gordon Setter | Y | NA | GORD | Gundog | Pointing Dogs | Large | Mesocephalic |
| Great Dane | Y | NA | DANE | Working | Pinscher and Schnauzer - Molossoid and Swiss Mountain and Cattledogs | Large | Dolichocephalic |
| Great Pyrenees | Y | Pyrenean Mountain Dog | GPYR | Pastoral | Pinscher and Schnauzer - Molossoid and Swiss Mountain and Cattledogs | Large | Mesocephalic |
| Greyhound | Y | NA | GREY | Hound | Sighthounds | Large | Dolichocephalic |
| Havanese | Y | NA | HAVA | Toy | Companion and Toy Dogs | Small | Mesocephalic |
| Irish Setter | Y | Irish Red & White Setter | ISET | Gundog | Pointing Dogs | Large | Mesocephalic |
| Irish Terrier | Y | NA | IRIT | Terrier | Terriers | Small | Dolichocephalic |
| Irish Water Spaniel | Y | NA | IWSP | Gundog | Retrievers - Flushing Dogs - Water Dogs | Medium | Mesocephalic |
| Irish Wolfhound | Y | NA | IWOF | Hound | Sighthounds | Large | Dolichocephalic |
| Italian Greyhound | Y | NA | ITGY | Toy | Sighthounds | Small | Dolichocephalic |
| Jack Russell Terrier | Y | NA | JACK | Terrier | Terriers | Small | Mesocephalic |
| Japanese Chin | Y | NA | CHIN | Toy | Companion and Toy Dogs | Small | Brachycephalic |
| Japanese Spitz | Y | NA | GSPM | Utility | Spitz and primitive types | Small | Mesocephalic |
| Keeshond | Y | NA | KEES | Utility | Spitz and primitive types | Medium | Mesocephalic |
| Kelpie | Y | Australian Kelpie | KELP | NA | Sheepdogs and cattledogs (except Swiss Cattledogs) | Medium | Dolichocephalic |
| Kerry Blue Terrier | Y | NA | KERY | Terrier | Terriers | Medium | Dolichocephalic |
| Labrador Retriever | Y | NA | LAB | Gundog | Retrievers - Flushing Dogs - Water Dogs | Large | Mesocephalic |
| Lakeland Terrier | Y | NA | LATE | Terrier | Terriers | Small | Dolichocephalic |
| Lancashire Heeler | Y | NA | NA | Pastoral | Sheepdogs and cattledogs (except Swiss Cattledogs) | Small | Mesocephalic |
| Large Munsterlander | Y | NA | LMUN | Gundog | Pointing Dogs | Large | Mesocephalic |
| Leonberger | Y | NA | LEON | Working | Pinscher and Schnauzer - Molossoid and Swiss Mountain and Cattledogs | Large | Mesocephalic |
| Lhasa Apso | Y | NA | LHSA | Utility | Companion and Toy Dogs | Small | Brachycephalic |
| Lowchen | Y | NA | NA | Toy | Companion and Toy Dogs | Small | Mesocephalic |
| Maltese | Y | NA | MALT | Toy | Companion and Toy Dogs | Small | Mesocephalic |
| Mastiff | Y | NA | MAST | Working | Pinscher and Schnauzer - Molossoid and Swiss Mountain and Cattledogs | Large | Brachycephalic |
| Miniature Bull Terrier | Y | NA | MBLT | Terrier | Terriers | Small | Dolichocephalic |
| Miniature Dachshund | Y | Dachshund (Miniature Long-Haired)  Dachshund (Miniature Wire-Haired)  Dachshund (Rabbit Long-Haired)  Dachshund (Rabbit Smooth-Haired)  Dachshund (Rabbit Wire-Haired) | MDCH | Hound | Dachshunds | Small | Dolichocephalic |
| Miniature Pinscher | Y | NA | MPIN | Toy | Pinscher and Schnauzer - Molossoid and Swiss Mountain and Cattledogs | Small | Dolichocephalic |
| Miniature Schnauzer | Y | NA | MSNZ | Utility | Pinscher and Schnauzer - Molossoid and Swiss Mountain and Cattledogs | Small | Mesocephalic |
| Neapolitan Mastiff | Y | NA | NEAP | Working | Pinscher and Schnauzer - Molossoid and Swiss Mountain and Cattledogs | Large | Brachycephalic |
| Newfoundland | Y | NA | NEWF | Working | Pinscher and Schnauzer - Molossoid and Swiss Mountain and Cattledogs | Large | Mesocephalic |
| Norfolk Terrier | Y | NA | NORF | Terrier | Terriers | Small | Mesocephalic |
| Norwegian Elkhound | Y | NA | NELK | Hound | Spitz and primitive types | Medium | Mesocephalic |
| Norwich Terrier | Y | NA | NOWT | Terrier | Terriers | Small | Mesocephalic |
| Nova Scotia Duck Tolling Retriever | Y | NA | NSDT | Gundog | Retrievers - Flushing Dogs - Water Dogs | Medium | Mesocephalic |
| Old English Sheepdog | Y | NA | OES | Pastoral | Sheepdogs and cattledogs (except Swiss Cattledogs) | Large | Mesocephalic |
| Papillon | Y | NA | PAPI | Toy | Companion and Toy Dogs | Small | Mesocephalic |
| Parson Russell Terrier | Y | NA | PARS | Terrier | Terriers | Small | Mesocephalic |
| Pekingese | Y | NA | PEKE | Toy | Companion and Toy Dogs | Small | Brachycephalic |
| Pembroke Welsh Corgi | Y | NA | PEMB | Pastoral | Sheepdogs and cattledogs (except Swiss Cattledogs) | Small | Mesocephalic |
| Petite Basset Griffon Vendeen | Y | Basset Fauve de Bretagne  Basset Griffon Vendeen (Grand)  Petite Basset Griffon Vendeen | PBGV | Hound | Scent hounds and related breeds | Medium | Dolichocephalic |
| Pharaoh Hound | Y | NA | PHAR | Hound | Spitz and primitive types | Medium | Dolichocephalic |
| Polish Lowland Sheepdog | Y | NA | POLS | Pastoral | Sheepdogs and cattledogs (except Swiss Cattledogs) | Medium | Mesocephalic |
| Pomeranian | Y | NA | POM | Toy | Spitz and primitive types | Small | Mesocephalic |
| Poodle | Y | NA | SPOO | Utility | Companion and Toy Dogs | NA | Dolichocephalic |
| Portuguese Water Dog | Y | NA | PTWD | Working | Retrievers - Flushing Dogs - Water Dogs | Medium | Mesocephalic |
| Presa Canario | Y | NA | PRCA | NA | Pinscher and Schnauzer - Molossoid and Swiss Mountain and Cattledogs | Large | Mesocephalic |
| Pug | Y | NA | PUG | Toy | Companion and Toy Dogs | Small | Brachycephalic |
| Puli | Y | NA | PULI | Pastoral | Sheepdogs and cattledogs (except Swiss Cattledogs) | Medium | Mesocephalic |
| Rhodesian Ridgeback | Y | NA | RHOD | Hound | Scent hounds and related breeds | Large | Mesocephalic |
| Rottweiler | Y | NA | ROTT | Working | Pinscher and Schnauzer - Molossoid and Swiss Mountain and Cattledogs | Large | Mesocephalic |
| Saluki | Y | NA | SALU | Hound | Sighthounds | Medium | Dolichocephalic |
| Samoyed | Y | NA | SAMO | Pastoral | Spitz and primitive types | Medium | Mesocephalic |
| Schipperke | Y | NA | SKIP | Utility | Sheepdogs and cattledogs (except Swiss Cattledogs) | Small | Mesocephalic |
| Scottish Deerhound | Y | NA | DEER | Hound | Sighthounds | Large | Dolichocephalic |
| Scottish Terrier | Y | NA | SCOT | Terrier | Terriers | Small | Dolichocephalic |
| Sealyham Terrier | Y | NA | SEGU | Terrier | Terriers | Small | Mesocephalic |
| Shetland Sheepdog | Y | NA | SSHP | Pastoral | Sheepdogs and cattledogs (except Swiss Cattledogs) | Small | Dolichocephalic |
| Shiba Inu | Y | NA | SHIB | Utility | Spitz and primitive types | Small | Mesocephalic |
| Shih Tzu | Y | NA | SHIH | Utility | Companion and Toy Dogs | Small | Brachycephalic |
| Siberian Husky | Y | NA | HUSK | Working | Spitz and primitive types | Medium | Mesocephalic |
| Silky Terrier | Y | Australian Silky Terrier | SILK | Toy | Terriers | Small | Mesocephalic |
| Skye Terrier | Y | NA | SKTE | Terrier | Terriers | Small | Mesocephalic |
| Soft Coated Wheaten Terrier | Y | NA | SCWT | Terrier | Terriers | Medium | Mesocephalic |
| Spanish Water Dog | Y | NA | NA | Gundog | Retrievers - Flushing Dogs - Water Dogs | Medium | Mesocephalic |
| Spinone Italiano | Y | NA | SPIN | Gundog | Pointing Dogs | Large | Mesocephalic |
| St Bernard | Y | NA | STBD | Working | Pinscher and Schnauzer - Molossoid and Swiss Mountain and Cattledogs | Large | Mesocephalic |
| Staffordshire Bull Terrier | Y | NA | STAF | Terrier | Terriers | Small | Mesocephalic |
| Standard Schnauzer | Y | Schnauzer | SSNZ | Utility | Pinscher and Schnauzer - Molossoid and Swiss Mountain and Cattledogs | Medium | Mesocephalic |
| Sussex Spaniel | Y | NA | SUSP | Gundog | Retrievers - Flushing Dogs - Water Dogs | Medium | Mesocephalic |
| Swedish Valhund | Y | NA | SVAL | Pastoral | Spitz and primitive types | Small | Mesocephalic |
| Tibetan Mastiff | Y | NA | TIBM | Working | Pinscher and Schnauzer - Molossoid and Swiss Mountain and Cattledogs | Large | Brachycephalic |
| Tibetan Spaniel | Y | NA | TIBS | Utility | Companion and Toy Dogs | Small | Mesocephalic |
| Tibetan Terrier | Y | NA | TIBT | Utility | Companion and Toy Dogs | Small | Mesocephalic |
| Toy Fox Terrier | Y | English Toy Terrier | TYFX | Toy | Terriers | Small | Mesocephalic |
| Toy Manchester Terrier | Y | NA | MNTY | Terrier | Terriers | Small | Dolichocephalic |
| Vizsla | Y | Hungarian Vizsla (Hungarian Shorthaired Pointer)  Hungarian Wirehaired Vizsla (Hungarian Wirehaired Pointer) | VIZS | Gundog | Pointing Dogs | Medium | Mesocephalic |
| Weimaraner | Y | NA | WEIM | Gundog | Pointing Dogs | Large | Mesocephalic |
| Welsh Springer Spaniel | Y | NA | WESS | Gundog | Retrievers - Flushing Dogs - Water Dogs | Medium | Mesocephalic |
| Welsh Terrier | Y | NA | WETE | Terrier | Terriers | Small | Dolichocephalic |
| West Highland White Terrier | Y | NA | WHWT | Terrier | Terriers | Small | Mesocephalic |
| Whippet | Y | NA | WHIP | Hound | Sighthounds | Small | Dolichocephalic |
| Wire Fox Terrier | Y | Fox Terrier (Smooth)  Fox Terrier (Wire) | WFOX | Terrier | Terriers | Small | Dolichocephalic |
| Yorkshire Terrier | Y | NA | YORK | Toy | Terriers | Small | Mesocephalic |
| Alpine Dachsbracke | N | NA | NA | NA | Scent hounds and related breeds | Medium | Mesocephalic |
| American Foxhound | N | NA | NA | NA | Scent hounds and related breeds | Medium | Mesocephalic |
| American Hairless Terrier | N | NA | AMHT | NA | NA | NA | NA |
| American Water Spaniel | N | NA | NA | Gundog | Retrievers - Flushing Dogs - Water Dogs | Medium | Mesocephalic |
| Apennine Wolf | N | NA | APWO | NA | NA | NA | NA |
| Appenzell Cattle Dog | N | NA | NA | NA | Pinscher and Schnauzer - Molossoid and Swiss Mountain and Cattledogs | Medium | Mesocephalic |
| Ariege Pointing Dog | N | NA | NA | NA | Pointing Dogs | Medium | Mesocephalic |
| Ariegeois | N | NA | NA | NA | Scent hounds and related breeds | Medium | Mesocephalic |
| Artois Hound | N | NA | NA | NA | Scent hounds and related breeds | Medium | Mesocephalic |
| Atlas Mountain Dog (Aidi) | N | NA | NA | NA | Pinscher and Schnauzer - Molossoid and Swiss Mountain and Cattledogs | Medium | Mesocephalic |
| Australian Terrier | N | NA | AUST | Terrier | Terriers | Small | Mesocephalic |
| Austrian Black & Tan Hound | N | NA | NA | NA | Scent hounds and related breeds | Medium | Mesocephalic |
| Austrian Pinscher | N | NA | NA | NA | Pinscher and Schnauzer - Molossoid and Swiss Mountain and Cattledogs | Medium | Dolichocephalic |
| Auvergne Pointer (Braque D'Auvergne) | N | NA | NA | Gundog | Pointing Dogs | Medium | Mesocephalic |
| Azawakh | N | NA | AZAW | Hound | Sighthounds | Medium | Dolichocephalic |
| Barbet | N | NA | NA | Gundog | Retrievers - Flushing Dogs - Water Dogs | Medium | Mesocephalic |
| Basset Artesien Normand (Norman Artesien Basset) | N | NA | NA | NA | Scent hounds and related breeds | Medium | Dolichocephalic |
| Basset Bleu De Gascogne (Blue Gascony Basset) | N | NA | NA | Hound | Scent hounds and related breeds | Medium | Dolichocephalic |
| Bavarian Mountain Hound | N | NA | NA | Hound | Scent hounds and related breeds | Medium | Mesocephalic |
| Beagle Harrier | N | NA | NA | NA | Scent hounds and related breeds | Medium | Mesocephalic |
| Beauceron (Berger de Beauce) | N | NA | NA | Pastoral | Sheepdogs and cattledogs (except Swiss Cattledogs) | Large | Mesocephalic |
| Belgian Lakenois | N | NA | NA | Pastoral | Sheepdogs and cattledogs (except Swiss Cattledogs) | Medium | Dolichocephalic |
| Bergamasco | N | NA | BERM | Pastoral | Sheepdogs and cattledogs (except Swiss Cattledogs) | Large | Mesocephalic |
| Berger Picard | N | Picardy Sheepdog | BERG | Pastoral | Sheepdogs and cattledogs (except Swiss Cattledogs) | Medium | Mesocephalic |
| Billy | N | NA | NA | NA | Scent hounds and related breeds | Medium | Mesocephalic |
| Black & Tan Coonhound | N | NA | NA | Hound | Scent hounds and related breeds | Large | Mesocephalic |
| Blue Picardy Spaniel | N | NA | NA | NA | Pointing Dogs | Medium | Mesocephalic |
| Bosnian and Herzegovinian - Croatian Shepherd Dog (Tornjak) | N | NA | NA | NA | Pinscher and Schnauzer - Molossoid and Swiss Mountain and Cattledogs | Large | Dolichocephalic |
| Bosnian Broken-Haired Hound | N | NA | NA | NA | Scent hounds and related breeds | Medium | Mesocephalic |
| Bourbonnais Pointing Dog | N | NA | NA | NA | Pointing Dogs | Medium | Mesocephalic |
| Bouvier des Ardennes | N | NA | NA | NA | Sheepdogs and cattledogs (except Swiss Cattledogs) | Large | Mesocephalic |
| Brazilian Terrier | N | NA | NA | NA | Terriers | Medium | Mesocephalic |
| Briquet Griffon Vendeen | N | NA | NA | NA | Scent hounds and related breeds | Medium | Mesocephalic |
| Broholmer | N | NA | NA | NA | Pinscher and Schnauzer - Molossoid and Swiss Mountain and Cattledogs | Large | Mesocephalic |
| Burgos Pointing Dog | N | NA | NA | NA | Pointing Dogs | Large | Mesocephalic |
| Canarian Warren Hound | N | NA | NA | NA | Spitz and primitive types | Medium | Dolichocephalic |
| Castro Laboreiro Dog | N | NA | NA | NA | NA | NA | NA |
| Catalan Sheepdog | N | NA | NA | Pastoral | Sheepdogs and cattledogs (except Swiss Cattledogs) | Medium | Mesocephalic |
| Central Asian Shepherd Dog | N | NA | CASD | NA | Pinscher and Schnauzer - Molossoid and Swiss Mountain and Cattledogs | Large | Mesocephalic |
| Cesky Terrier | N | NA | NA | Terrier | Terriers | Small | Mesocephalic |
| Cimarron Uruguayo | N | NA | NA | NA | Pinscher and Schnauzer - Molossoid and Swiss Mountain and Cattledogs | Medium | Mesocephalic |
| Cirneco dellEtna | N | NA | CIRN | Hound | Spitz and primitive types | Medium | Dolichocephalic |
| Coarse-Haired Styrian Hound | N | NA | NA | NA | Scent hounds and related breeds | Medium | Mesocephalic |
| Croatian Shepherd Dog | N | NA | NA | NA | Sheepdogs and cattledogs (except Swiss Cattledogs) | Medium | Dolichocephalic |
| Czechoslovakian Wolfdog | N | NA | NA | NA | Sheepdogs and cattledogs (except Swiss Cattledogs) | Large | Dolichocephalic |
| Danish-Swedish Farmdog | N | NA | NA | NA | Pinscher and Schnauzer - Molossoid and Swiss Mountain and Cattledogs | Small | Mesocephalic |
| Dogo Argentino | N | NA | NA | NA | Pinscher and Schnauzer - Molossoid and Swiss Mountain and Cattledogs | Large | Mesocephalic |
| Drentsche Partridge Dog | N | NA | NA | NA | Pointing Dogs | Medium | Mesocephalic |
| Drever | N | NA | NA | NA | Scent hounds and related breeds | Medium | Mesocephalic |
| Dutch Schapendoes | N | NA | NA | NA | Sheepdogs and cattledogs (except Swiss Cattledogs) | Medium | Mesocephalic |
| Dutch Shepherd Dog | N | NA | NA | NA | Sheepdogs and cattledogs (except Swiss Cattledogs) | Medium | Dolichocephalic |
| Dutch Smoushond | N | NA | NA | NA | Pinscher and Schnauzer - Molossoid and Swiss Mountain and Cattledogs | Small | Mesocephalic |
| East Siberia Laika | N | NA | NA | NA | Spitz and primitive types | Medium | Mesocephalic |
| Entlebucher Mountain Dog | N | NA | NA | Working | Pinscher and Schnauzer - Molossoid and Swiss Mountain and Cattledogs | Medium | Mesocephalic |
| Estrela Mountain Dog | N | NA | NA | Pastoral | Pinscher and Schnauzer - Molossoid and Swiss Mountain and Cattledogs | Large | Mesocephalic |
| Eurasier | N | NA | EURA | Utility | Spitz and primitive types | Medium | Mesocephalic |
| Fila Brasileiro | N | NA | NA | NA | Pinscher and Schnauzer - Molossoid and Swiss Mountain and Cattledogs | Large | Brachycephalic |
| Finnish Hound | N | NA | NA | NA | Scent hounds and related breeds | Medium | Mesocephalic |
| Finnish Spitz | N | NA | FNSP | Hound | Spitz and primitive types | Small | Mesocephalic |
| French Pointing Dog (Gascogne Type) | N | NA | NA | NA | Pointing Dogs | Medium | Mesocephalic |
| French Pointing Dog (Pyrenean Type) | N | NA | NA | NA | Pointing Dogs | Medium | Mesocephalic |
| French Spaniel | N | NA | NA | NA | Pointing Dogs | Medium | Mesocephalic |
| French Tricolour Hound | N | NA | NA | NA | Scent hounds and related breeds | Medium | Mesocephalic |
| French White & Black Hound | N | NA | NA | NA | Scent hounds and related breeds | Medium | Mesocephalic |
| French White & Orange Hound | N | NA | NA | NA | Scent hounds and related breeds | Medium | Mesocephalic |
| Frisian Water Dog | N | NA | NA | NA | Retrievers - Flushing Dogs - Water Dogs | Medium | Mesocephalic |
| Gascon Saintongeois | N | NA | NA | NA | Scent hounds and related breeds | Large | Dolichocephalic |
| German Hound | N | NA | NA | NA | Scent hounds and related breeds | Medium | Mesocephalic |
| German Hunting Terrier | N | NA | NA | NA | Terriers | Medium | Mesocephalic |
| German Spaniel | N | NA | NA | NA | Retrievers - Flushing Dogs - Water Dogs | Medium | Mesocephalic |
| German Spitz (Giant) | N | NA | NA | Utility | Spitz and primitive types | Medium | Mesocephalic |
| German Spitz (Klein) | N | NA | NA | Utility | Spitz and primitive types | Small | Mesocephalic |
| German Spitz (Medium) | N | NA | NA | Utility | Spitz and primitive types | Medium | Mesocephalic |
| German Spitz (Miniature) | N | NA | NA | Utility | Spitz and primitive types | Small | Mesocephalic |
| Grand Bleu Gascogne | N | NA | NA | Hound | Scent hounds and related breeds | Large | Dolichocephalic |
| Great Anglo-French Hound | N | NA | NA | NA | Scent hounds and related breeds | Medium | Mesocephalic |
| Great Anglo-French Tricolour Hound | N | NA | NA | NA | Scent hounds and related breeds | Large | Mesocephalic |
| Great Anglo-French White & Black Hound | N | NA | NA | NA | Scent hounds and related breeds | Large | Mesocephalic |
| Great Anglo-French White & Orange Hound | N | NA | NA | NA | Scent hounds and related breeds | Large | Mesocephalic |
| Greater Swiss Mountain Dog | N | NA | GSMD | Working | Pinscher and Schnauzer - Molossoid and Swiss Mountain and Cattledogs | Large | Mesocephalic |
| Greenland Sledge Dog | N | Greenland Dog | GREN | Working | Spitz and primitive types | Large | Mesocephalic |
| Griffon Belge | N | NA | NA | NA | Companion and Toy Dogs | Small | Brachycephalic |
| Griffon Bleu de Gascogne (Blue Gascony Griffon) | N | NA | NA | NA | Scent hounds and related breeds | Medium | Dolichocephalic |
| Griffon Fauve De Bretagne (Fawn Brittany Griffon) | N | NA | NA | Hound | Scent hounds and related breeds | Medium | Mesocephalic |
| Griffon Nivernais | N | NA | NA | NA | Scent hounds and related breeds | Medium | Mesocephalic |
| Halden Hound | N | NA | NA | NA | Scent hounds and related breeds | Medium | Mesocephalic |
| Hamiltonstovare | N | NA | NA | Hound | Scent hounds and related breeds | Medium | Mesocephalic |
| Hanoverian Scent Hound | N | NA | NA | NA | Scent hounds and related breeds | Large | Mesocephalic |
| Harrier | N | NA | NA | NA | Scent hounds and related breeds | Medium | Mesocephalic |
| Hellenic Hound | N | NA | NA | NA | Scent hounds and related breeds | Medium | Mesocephalic |
| Hokkaido | N | NA | NA | NA | Spitz and primitive types | Medium | Mesocephalic |
| Hovawart | N | NA | NA | Working | Pinscher and Schnauzer - Molossoid and Swiss Mountain and Cattledogs | Large | Mesocephalic |
| Hungarian Greyhound (Magyar Agar) | N | NA | NA | NA | Sighthounds | Medium | Dolichocephalic |
| Hungarian Hound - Transylvanian Scent Hound | N | NA | NA | NA | Scent hounds and related breeds | Medium | Dolichocephalic |
| Hygen Hound | N | NA | NA | NA | Scent hounds and related breeds | Medium | Mesocephalic |
| Ibizan Hound | N | NA | IBZA | Hound | Spitz and primitive types | Medium | Dolichocephalic |
| Icelandic Sheepdog | N | NA | ICEL | NA | Spitz and primitive types | Medium | Mesocephalic |
| Istrian Short-Haired Hound | N | NA | NA | NA | Scent hounds and related breeds | Medium | Mesocephalic |
| Istrian Wire-Haired Hound | N | NA | NA | NA | Scent hounds and related breeds | Medium | Mesocephalic |
| Jamthund | N | NA | NA | NA | Spitz and primitive types | Medium | Mesocephalic |
| Japenese Terrier | N | NA | NA | NA | Terriers | Small | Mesocephalic |
| Kai | N | NA | NA | NA | Spitz and primitive types | Medium | Mesocephalic |
| Karelian Bear Dog | N | NA | NA | NA | Spitz and primitive types | Medium | Mesocephalic |
| Karst Shepherd Dog | N | NA | NA | NA | Pinscher and Schnauzer - Molossoid and Swiss Mountain and Cattledogs | Medium | Mesocephalic |
| Kishu | N | NA | NA | NA | Spitz and primitive types | Medium | Mesocephalic |
| Komondor | N | NA | KOMO | Pastoral | Sheepdogs and cattledogs (except Swiss Cattledogs) | Large | Mesocephalic |
| Kooikerhondje | N | NA | NA | Utility | Retrievers - Flushing Dogs - Water Dogs | Small | Mesocephalic |
| Korean Jindo | N | NA | NA | Utility | Spitz and primitive types | Medium | Mesocephalic |
| Korthals Griffon | N | NA | NA | Gundog | Pointing Dogs | Medium | Mesocephalic |
| Kromfohrlander | N | NA | NA | NA | Companion and Toy Dogs | Medium | Mesocephalic |
| Kuvasz | N | NA | KUVA | Pastoral | Sheepdogs and cattledogs (except Swiss Cattledogs) | Large | Mesocephalic |
| Lagotto Romagnolo | N | NA | LAGO | Gundog | Retrievers - Flushing Dogs - Water Dogs | Small | Mesocephalic |
| Landseer (European Continental Type) | N | NA | NA | NA | Pinscher and Schnauzer - Molossoid and Swiss Mountain and Cattledogs | Large | Mesocephalic |
| Lapponian Herder | N | NA | NA | NA | Spitz and primitive types | Medium | Mesocephalic |
| Majorca Mastiff | N | NA | NA | NA | Pinscher and Schnauzer - Molossoid and Swiss Mountain and Cattledogs | Large | Brachycephalic |
| Majorca Shepherd Dog | N | NA | NA | NA | Sheepdogs and cattledogs (except Swiss Cattledogs) | Medium | Mesocephalic |
| Maremma Shepherd | N | NA | MRM | Pastoral | Sheepdogs and cattledogs (except Swiss Cattledogs) | Large | Mesocephalic |
| Medium-Sized Anglo-French Hound | N | NA | NA | NA | Scent hounds and related breeds | Medium | Dolichocephalic |
| Miniature & Standard Xoloitzcuintle | N | Mexican Hairless | XOLO | Utility | Spitz and primitive types | Small | Dolichocephalic |
| Montenegrin Mountain Hound | N | NA | NA | NA | Scent hounds and related breeds | Medium | Mesocephalic |
| Mudi | N | NA | NA | NA | Sheepdogs and cattledogs (except Swiss Cattledogs) | Medium | Mesocephalic |
| Norrbottenspitz | N | NA | NA | NA | Spitz and primitive types | Medium | Mesocephalic |
| Norwegian Buhund | N | NA | NA | Pastoral | Spitz and primitive types | Medium | Mesocephalic |
| Norwegian Hound | N | NA | NA | NA | Scent hounds and related breeds | Medium | Mesocephalic |
| Norwegian Lundehund | N | NA | NA | NA | Spitz and primitive types | Small | Mesocephalic |
| Old Danish Pointing Dog | N | NA | NA | NA | Pointing Dogs | Medium | Mesocephalic |
| Otterhound | N | NA | OTTE | Hound | Scent hounds and related breeds | Large | Mesocephalic |
| Pastor della Sila | N | NA | SILA | NA | NA | NA | NA |
| Pastore della Lessinia e del Lagorai | N | NA | DELLA | NA | NA | NA | NA |
| Peruvian Inca Orchid | N | Peruvian Hairless Dog | PERU | NA | Spitz and primitive types | NA | Dolichocephalic |
| Petit Bleu de Gascogne (Small Blue Gascony) | N | NA | NA | Hound | Scent hounds and related breeds | Medium | Dolichocephalic |
| Petit Brabancon | N | NA | NA | NA | Companion and Toy Dogs | Small | Brachycephalic |
| Phalene | N | NA | NA | NA | Companion and Toy Dogs | Small | Mesocephalic |
| Picardy Spaniel | N | NA | NA | NA | Pointing Dogs | Medium | Mesocephalic |
| Poitevin | N | NA | NA | NA | Scent hounds and related breeds | Medium | Dolichocephalic |
| Polish Greyhound | N | NA | NA | NA | Sighthounds | Medium | Dolichocephalic |
| Polish Hound | N | NA | NA | NA | Scent hounds and related breeds | Medium | Mesocephalic |
| Polish Hunting Dog | N | NA | NA | NA | Scent hounds and related breeds | Large | Mesocephalic |
| Pont-Audemer Spaniel | N | NA | NA | NA | Pointing Dogs | Medium | Mesocephalic |
| Porcelaine | N | NA | NA | NA | Scent hounds and related breeds | Medium | Dolichocephalic |
| Portugese Pointing Dog | N | NA | NA | Gundog | Pointing Dogs | Medium | Mesocephalic |
| Portuguese Podengo (Warren Hound) | N | NA | NA | Hound | Spitz and primitive types | Small | Dolichocephalic |
| Portuguese Sheepdog | N | NA | NA | NA | Sheepdogs and cattledogs (except Swiss Cattledogs) | Medium | Mesocephalic |
| Posavatz Hound | N | NA | NA | NA | Scent hounds and related breeds | Medium | Mesocephalic |
| Pudelpointer | N | NA | NA | NA | Pointing Dogs | Medium | Mesocephalic |
| Pumi | N | NA | PUM | Pastoral | Sheepdogs and cattledogs (except Swiss Cattledogs) | Small | Mesocephalic |
| Pyrenean Mastiff | N | NA | NA | Working | Pinscher and Schnauzer - Molossoid and Swiss Mountain and Cattledogs | Large | Brachycephalic |
| Pyrenean Sheepdog (Long-Haired) | N | NA | NA | Pastoral | Sheepdogs and cattledogs (except Swiss Cattledogs) | Medium | Mesocephalic |
| Pyrenean Sheepdog (Smooth-Faced) | N | NA | NA | NA | Sheepdogs and cattledogs (except Swiss Cattledogs) | Medium | Mesocephalic |
| Rafeiro of Alentejo | N | NA | NA | NA | Pinscher and Schnauzer - Molossoid and Swiss Mountain and Cattledogs | Large | Mesocephalic |
| Romanian Bucovina Shepherd | N | NA | NA | NA | Pinscher and Schnauzer - Molossoid and Swiss Mountain and Cattledogs | Large | Mesocephalic |
| Romanian Carpathian Shepherd Dog | N | NA | NA | NA | Sheepdogs and cattledogs (except Swiss Cattledogs) | Large | Dolichocephalic |
| Romanian Mioritic Shepherd Dog | N | NA | NA | NA | Sheepdogs and cattledogs (except Swiss Cattledogs) | Large | Mesocephalic |
| Russian Toy | N | NA | NA | Toy | Companion and Toy Dogs | Small | Mesocephalic |
| Russian-European Laika | N | NA | NA | NA | Spitz and primitive types | Medium | Mesocephalic |
| Saarloos Wolfhound | N | NA | NA | NA | Sheepdogs and cattledogs (except Swiss Cattledogs) | Large | Dolichocephalic |
| Saint Germain Pointer | N | NA | NA | NA | Pointing Dogs | Medium | Mesocephalic |
| Saint Miguel Cattle Dog | N | NA | NA | NA | Pinscher and Schnauzer - Molossoid and Swiss Mountain and Cattledogs | Large | Mesocephalic |
| Schillerstovare | N | NA | NA | NA | Scent hounds and related breeds | Medium | Mesocephalic |
| Segugio Italiano Pelo Raso & Pelo Forte | N | Italian Rough-Haired Segugio  Italian Short-Haired Segugio | SEGU | Hound | Scent hounds and related breeds | Large | Dolichocephalic |
| Serbian Hound | N | NA | NA | NA | Scent hounds and related breeds | Medium | Mesocephalic |
| Serbian Tricolour Hound | N | NA | NA | NA | Scent hounds and related breeds | Medium | Mesocephalic |
| Shikoku | N | NA | NA | NA | Spitz and primitive types | Medium | Mesocephalic |
| Sloughi | N | NA | SLOU | Hound | Sighthounds | Medium | Dolichocephalic |
| Slovakian Chuvach | N | NA | NA | NA | Sheepdogs and cattledogs (except Swiss Cattledogs) | Large | Mesocephalic |
| Slovakian Hound | N | NA | NA | NA | Scent hounds and related breeds | Medium | Mesocephalic |
| Slovakian Rough Haired Pointer | N | NA | NA | Gundog | Pointing Dogs | Large | Mesocephalic |
| Smalandsstovare | N | NA | NA | NA | Scent hounds and related breeds | Medium | Mesocephalic |
| Small Munsterlander | N | NA | NA | Gundog | Pointing Dogs | Medium | Mesocephalic |
| South Russian Shepherd Dog | N | NA | NA | NA | Sheepdogs and cattledogs (except Swiss Cattledogs) | Large | Mesocephalic |
| Spanish Greyhound | N | NA | NA | NA | Sighthounds | Large | Dolichocephalic |
| Spanish Hound | N | NA | NA | NA | Scent hounds and related breeds | Medium | Dolichocephalic |
| Spanish Mastiff | N | NA | SPMA | NA | Pinscher and Schnauzer - Molossoid and Swiss Mountain and Cattledogs | Large | Brachycephalic |
| Stabijhoun | N | NA | NA | NA | Pointing Dogs | Medium | Mesocephalic |
| Swedish Lapphund | N | NA | NA | Pastoral | Spitz and primitive types | Medium | Mesocephalic |
| Swiss Hound | N | NA | NA | NA | Scent hounds and related breeds | Medium | Dolichocephalic |
| Taiwan Dog | N | NA | NA | NA | Spitz and primitive types | Medium | Mesocephalic |
| Tatra Shepherd Dog | N | NA | NA | NA | Sheepdogs and cattledogs (except Swiss Cattledogs) | Large | Mesocephalic |
| Thai Ridgeback Dog | N | NA | NA | NA | Spitz and primitive types | Medium | Mesocephalic |
| Tosa | N | NA | NA | NA | Pinscher and Schnauzer - Molossoid and Swiss Mountain and Cattledogs | Large | Mesocephalic |
| Turkish Kangal Dog | N | NA | NA | Pastoral | Pinscher and Schnauzer - Molossoid and Swiss Mountain and Cattledogs | Large | Dolichocephalic |
| Tyrolean Hound | N | NA | NA | NA | Scent hounds and related breeds | Medium | Mesocephalic |
| Volpino Italiano | N | NA | VOLP | NA | Spitz and primitive types | Small | Mesocephalic |
| West Siberian Laika | N | NA | NA | NA | Spitz and primitive types | Medium | Mesocephalic |
| Westphalian Dachsbracke | N | NA | NA | NA | Scent hounds and related breeds | Small | Mesocephalic |
| White Swiss Shepherd Dog | N | NA | NA | Pastoral | Sheepdogs and cattledogs (except Swiss Cattledogs) | Large | Dolichocephalic |
| Wirehaired Pointing Griffon | N | Bohemian Wire-Haired Pointing Griffon | WHPG | NA | Pointing Dogs | Medium | Mesocephalic |
| Wolf & Golden Jackal | N | NA | WGJ | NA | NA | NA | NA |
| Yugoslavian Shepherd Dog (Sharplanina) | N | NA | NA | NA | Pinscher and Schnauzer - Molossoid and Swiss Mountain and Cattledogs | Large | Mesocephalic |
