# Supplementary material for: Longevity of companion dog breeds: those at risk from early death
Source: Sci Rep. 2024 Feb 1;14:531. doi: 10.1038/s41598-023-50458-w (PMC10834484; doi:10.1038/s41598-023-50458-w)
Supplement: Supplementary file 11 — Supplementary Note S1. [file 41598_2023_50458_MOESM11_ESM.docx]

***Note S1:*** *Deduplication Methodology*

**Phase 1:** *Remove exact duplicates*

- Calculate percentage data available for each variable.
- Calculate column completeness for each row (number of NAs per row).
- Set missing threshold (= 10/18 variables), and match duplicates as long as missingness is less than or equal to threshold. Otherwise, keep rows (with more missing data).

**Phase 2:**

- Match DOB (month/year), breed, name, sex, and microchip - status excluded.
- Then, match DOB (month/year), breed, name, sex, and microchip - status included i.e., should be left with one status now.
- Check status of matches and keep 'dead'.

**Phase 3:**

- Find rows which have multiple microchips (split string for those with multiple microchips).
- Find all candidate rows that correspond to the alternative microchips.
- Merge candidate rows if DOB (month/year), breed, name, sex, microchip, and status are all equivalent.
- If matching records are all ‘alive’ or ‘dead’, deduplicate down to a single record.
- If matching records has both ‘alive’ and ‘dead’ records, keep one ‘alive’ row and one ‘dead row.
- We then match again, merging candidate rows if DOB (month/year), breed, name, sex, and microchip are all equivalent (i.e., excludes status).
- This pairs the dead + alive row, and we set the ‘alive’ in the pair to ‘dead’. Thus, all alive + dead pairs are now dead + dead.
- Finally, merge candidate rows if DOB (month/year), breed, name, sex, microchip, and status are all equivalent, thus deduplicating dead + dead pairs.

**Phase 4:**

- Subset data to those with no microchip numbers.
- Merge candidate rows if DOB (month/year), breed, name, sex, postcode, and status are all equivalent.
- If matching records are all ‘alive’ or ‘dead’, deduplicate down to a single record.
- If matching records has both ‘alive’ and ‘dead’ records, keep one ‘alive’ row and one ‘dead row.
- We then match again, merging candidate rows DOB (month/year), breed, name, sex, and postcode are all equivalent (i.e., excludes status).
- This pairs the dead + alive row, and we set the ‘alive’ in the pair to ‘dead’. Thus, all alive + dead pairs are now dead + dead.
- Finally, merge candidate rows if DOB (month/year), breed, name, sex, postcode, and status are all equivalent, thus deduplicating dead + dead pairs.
- Only duplicates left at this stage, are 1 copy with chip and one without chip (copies might have different STATUS).
- Deduplicate, based on DOB (month/year), breed, name, sex, postcode, and status. This will merge rows with and without chip that have a matching STATUS.
